# MMP28 recruits M2-type tumor-associated macrophages through MAPK/JNK signaling pathway-dependent cytokine secretion to promote the malignant progression of pancreatic cancer

**DOI:** 10.1186/s13046-025-03321-x

**Published:** 2025-02-19

**Authors:** Shi Dong, Xin Li, Zhou Chen, Huaqing Shi, Zhengfeng Wang, Wence Zhou

**Affiliations:** 1 The Second Hospital & Clinical Medical School, Lanzhou University, Lanzhou, 730000 China; 2 Department of General Surgery, The Second Hospital & Clinical Medical School, Lanzhou University, Lanzhou, 730000 China; 3 Department of General Surgery, The First Hospital of Lanzhou University, Lanzhou, 730000 China; 4 Department of Thoracic Surgery, The First Hospital of Lanzhou University, Lanzhou, 730000 China

**Keywords:** Pancreatic cancer, Tumor-associated macrophages, MMP28, Cytokines, MAPK pathway

## Abstract

**Background:**

Crosstalk between pancreatic cancer cells and tumor-associated macrophages (TAMs) is a critical driver of malignant progression, and plays an important role in the low response rate to immunotherapy in patients with for pancreatic cancer. Although it is known that cancer cells induce TAM infiltration and M2 polarization, the underlying mechanisms remain elusive. Herein, we identified matrix metalloproteinase 28 (MMP28), a highly expressed protein, as a key regulator of this process.

**Methods:**

Immunohistochemical staining and qRT-PCR were used to validate MMP28 as a potential marker for the prognosis of patients with pancreatic cancer. We evaluated the tumor-promoting effect of MMP28 in vitro with CCK-8, Transwell, and EdU assay and Western blotting and explored the potential mechanism of MMP28-induced M2 polarization of TAMs with a coculture system, immunofluorescence staining and flow cytometry. A subcutaneous graft tumor model was constructed to assess the tumor-promoting effect of MMP28 and its ability to induce M2 TAM infiltration.

**Results:**

The relevant results of this study revealed a strong correlation between MMP28 expression and TAM infiltration, with a predominance of M2-polarized TAMs in pancreatic cancer tissues. Mechanistic investigations demonstrated that MMP28 promotes the secretion of multiple cytokines, including IL-8 and VEGFA through the activation of the MAPK/JNK signaling pathway. These cytokines act as potent chemoattractants and polarizing factors for TAMs. Additionally, we discovered an interaction between MMP28 and ANXA2, which contributes to the regulation of TAM recruitment and polarization. In vivo studies confirmed the critical role of MMP28 in tumor growth and TAM infiltration. Depletion of macrophages, inhibition of JNK, or neutralization of IL-8 and VEGFA significantly suppressed tumor progression. Transcriptomic analysis suggested that IL-8 and VEGFA induce M2 TAM polarization by modulating TAM amino acid metabolism.

**Conclusions:**

Collectively, our findings elucidate a novel mechanism by which pancreatic cancer cells manipulate the tumor microenvironment through MMP28-dependent cytokine secretion, promoting TAM infiltration and M2 polarization. These results highlight MMP28 as a promising therapeutic target for pancreatic cancer.

**Graphical Abstract:**

Schematic overview of the mechanisms by which MMP28 promotes the migration and polarization of TAMs. High levels of MMP28 promote the secretion of IL-8 and VEGFA by cancer cells by mediating the phosphorylation of the MAPK/JNK signalling pathway and then recruiting TAMs. IL-8 and VEGFA subsequently induce amino acid metabolism alterations in TAMs by binding to relevant receptors on TAMs, which ultimately promote the polarization of TAMs to the M2 phenotype. In addition, ANXA2 increases MMP28-mediated M2 TAM infiltration by interacting with MMP28.

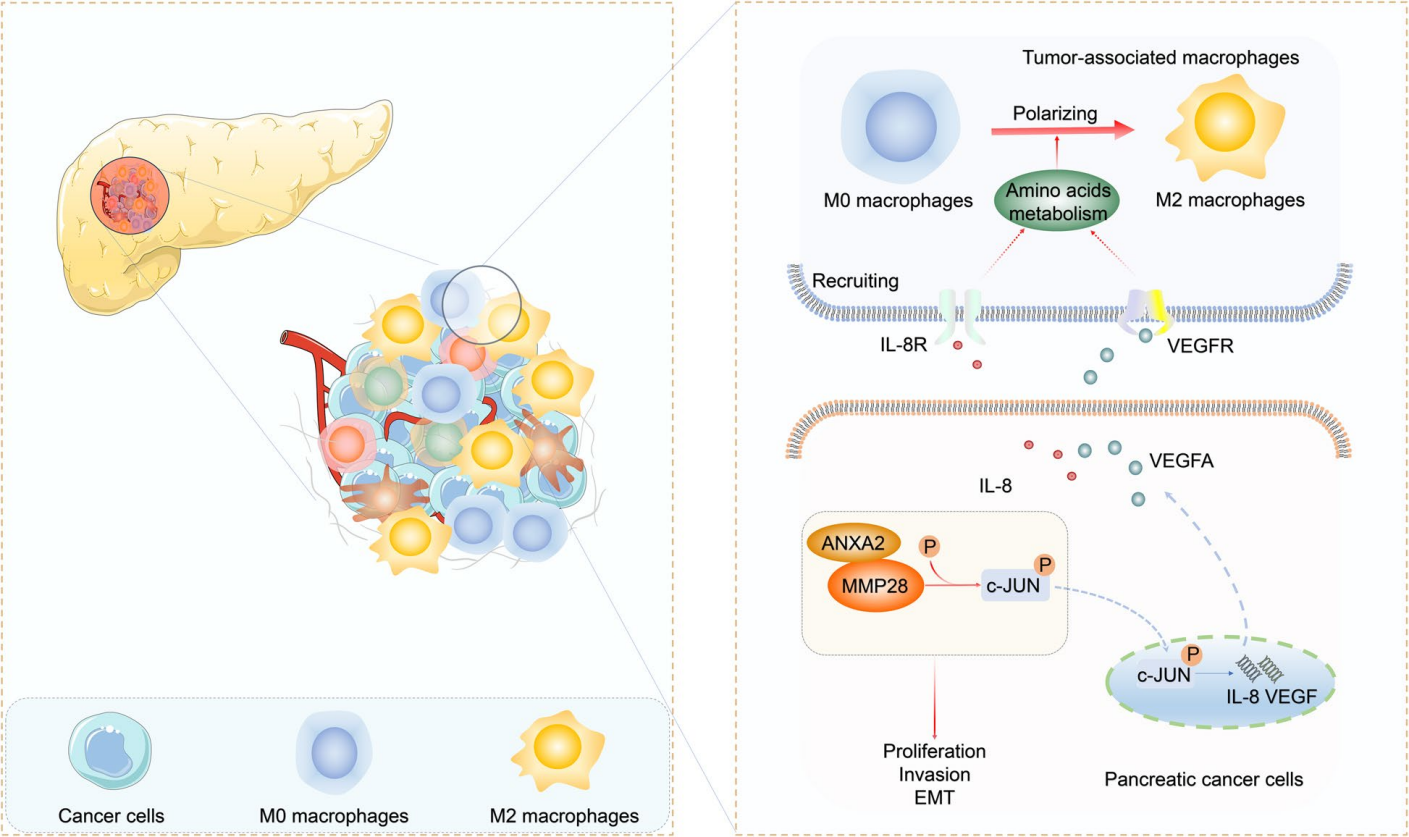

**Supplementary Information:**

The online version contains supplementary material available at 10.1186/s13046-025-03321-x.

## Introduction

Pancreatic cancer is one of the most malignant digestive tract tumours [1]. Owing to its delayed diagnosis and highly aggressive nature, more than 80% of pancreatic cancer patients present with advanced disease, resulting in a 5-year survival rate of less than 12% [2]. Moreover, pancreatic cancer will soon become the second leading cause of cancer-related death worldwide because of its increasing morbidity and mortality rates [3]. Despite advancements in standard care, existing therapies have limited efficacy in improving patient outcomes, underscoring the urgent need for novel therapeutic strategies.

The tumor microenvironment constitutes the immediate ecological niche essential for cancer survival and proliferation [4]. Tumor cells actively remodel this microenvironment by recruiting and reprogramming nonmalignant host cells and extracellular components to evade external pressures [5]. As immune cells extensively infiltrate the pancreatic cancer microenvironment, tumor-associated macrophages (TAMs) engage in complex interactions with various microenvironmental components, including cancer cells, to facilitate tumor progression [6]. TAMs exhibit phenotypic plasticity, and undergo polarization into distinct functional subsets on the basis of environmental cues. Classically activated (M1) macrophages, which are characterized by the expression of markers such as CD86 and iNOS, primarily contribute to inflammatory responses. Conversely, alternatively activated (M2) macrophages identified by markers such as CD163 and CD206, frequently infiltrate malignant tumors, driving cancer progression [7]. Numerous studies have demonstrated that cancer cells secrete a variety of chemokines and cytokines, including IL-10, CCL2, CCL18, and CXCL4, to recruit TAMs and establish an immunosuppressive microenvironment [8–10]. However, the precise mechanism underlying cancer cell-mediated TAM recruitment and M2 polarization within the intricate pancreatic cancer microenvironment remains largely unexplored.

Matrix metalloproteinases (MMPs) are critical regulators of the tumor microenvironment. These enzymes contribute to extracellular matrix degradation, angiogenesis, immune cell recruitment, and epithelial-mesenchymal transition (EMT), collectively promoting tumor invasion and metastasis [11]. MMP28, the most commonly identified MMP, possesses a prototypic MMP domain and is implicated in tumor progression. For example, Kruppel-like factor 9 (KLF9) suppresses gastric cancer cell invasion and metastasis by inhibiting MMP28 expression [12]. In liver cancer, MMP28 upregulation drives cell migration and invasion, which are correlated with tumor size, vascular invasion, TNM stage, and overall survival. MMP28 enhances EMT via the Notch signaling pathway [13]. While the role of MMP28 in pancreatic cancer remains incompletely understood, previous research has indicated its potential importance. Manicone et al. demonstrated that MMP28 promotes emphysema development by recruiting and activating macrophages through the regulation of immunoinflammatory genes in a mouse knockout model [14]. Our preliminary findings revealed that high MMP28 expression in pancreatic cancer tissues was associated with poor prognosis (unpublished). However, the precise molecular mechanisms underlying the impact of MMP28 on pancreatic cancer and its microenvironment require further elucidation.

This study investigated the role of MMP28 in pancreatic cancer progression. We demonstrated that modulating MMP28 expression altered the secretion of IL-8 and VEGFA by cancer cells, subsequently affecting TAM recruitment and M2 polarization. Mechanistic studies revealed that MMP28 promotes MAPK/JNK signaling pathway phosphorylation, thereby reshaping the tumor microenvironment. Additionally, the knockdown of annex protein A2 (ANXA2) attenuated MMP28 induced M2 TAM polarization. Our findings suggest that MMP28 enhances TAM recruitment and M2 polarization by stimulating cytokine secretion through the MAPK/JNK pathway, providing novel insights into potential therapeutic targets for pancreatic cancer.

## Materials and methods

### Bioinformatics tools

In this study, mRNA expression profiling data and associated clinical annotations were retrieved from publicly available resources: the Gene Expression Omnibus (GEO, http://www.ncbi.nlm.nih.gov/geo/), The Cancer Genome Atlas (TCGA, http://cancergenome.nih.gov/), and the UCSC Xena platform (https://xenabrowser.net/). The R software package "limma" (version 3.6.1) was used to identify differentially expressed genes (|LogFC|>1 and adjusted *P*-value < 0.05). The "survminer" and "survival" packages were subsequently used to analyse the impact of these differentially expressed genes on clinical prognosis.

### Clinical specimens

Pancreatic cancer and adjacent noncancerous tissues were obtained from 68 patients who underwent surgical resection at the General Surgery Departments of the First and Second Hospitals of Lanzhou University. Quantitative real-time PCR (qRT-PCR) was performed on all the tissues, while immunohistochemical staining analysis was conducted on a subset of 36 samples. All procedures were approved by the Ethical Review Committee (LDYYLL2021-108, 2023A-307), and informed consent was obtained from all participants.

### Cell culture

Human-derived pancreatic cancer cell lines (AsPC-1, BxPC-3, SW1990, and PANC-1) were obtained from the Cell Bank of the Chinese Academy of Sciences (Shanghai, China). The human normal pancreatic ductal epithelial cell line HPDE6-C7 was procured from the BeNa Culture Collection (Beijing, China), and the THP-1 cell line was purchased from Pricella (Wuhan, China). AsPC-1, BxPC-3, and THP-1 cells were cultured in RPMI-1640 medium (Gibco, Grand Island, USA), while SW1990, PANC-1, and HPDE6-C7 cells were maintained in DMEM medium. All culture media were supplemented with 10% foetal bovine serum (FBS, ABW, Uruguay) and 1% penicillin–streptomycin mixture (G4003-100 ML, Servicebio, Wuhan, China). The cells were incubated at 37 °C in a humidified atmosphere containing 5% CO_2_.

### Animal models

Male BALB/c nude mice (4–6 weeks old) were obtained from Cavens Laboratory Animals Ltd. (Changzhou, China) and maintained under strict specific pathogen-free (SPF) conditions. All animal experiments were approved by the Institutional Animal Care and Use Committee of the Second Hospital of Lanzhou University (Ethics Certificate number: D2024-053). For the xenograft tumor model, the mice were randomly assigned to groups (*n* = 5). The cells (1 × 10^6^) were subcutaneously injected into the right flank of each mouse. Tumor growth was monitored by bioluminescence imaging after intraderitoneal injection of D-(-)-luciferin potassium (150 mg/kg, HY-12591B, MCE, USA). When the tumors reached a volume of at least 80 mm^3^ one week post-injection, the treatment groups received the following treatments: PBS, Clodronate Liposomes (200 μL/mouse, YEASEN), the JNK inhibitor SP600125 (15 mg/kg/mouse, MCE), the interleukin-8 (IL-8)-neutralizing antibody MAB208-SP (10 μg/mouse, R&D Systems), or vascular endothelial growth factor A (VEGFA)-neutralizing antibody MAB293-SP (1 μg/mouse, R&D Systems). Four weeks post-injection, mice were euthanized, and the tumors were excised. Tumor tissues were subjected to immunohistochemical staining or stored at -80 °C for subsequent analysis.

### Lentiviral infection and transient cell transfection

To generate stable MMP28 knockout pancreatic cancer cell lines, cells were stably transfected with a lentiviral vector encoding GFP-Puro-MMP28 short hairpin RNA (shRNA) (sh-MMP28). An empty vector virus (sh-NC) served as a control. Conversely, stable MMP28 overexpression was achieved by transfecting cells with a lentiviral vector containing GFP-Puro-MMP28 cDNA. HitransG P (REVG005, Genechem, Shanghai, China) was used to enhance viral transfection efficiency in both cases. Following 48 h of lentiviral transfection, the medium was replaced with fresh medium containing 2 μg/mL puromycin (P8230, Solarbio) for one week to select stably transfected cells. For transient knockdown of ANXA2, cells were transiently transfected with ANXA2-targeting siRNA with the GP-transfect-Mate transfection reagent. The cells were harvested 48 h post-transfection to assess the knockdown efficiency. The specific target sequences are listed in Table S1.

### Cell Counting Kit-8 (CCK-8) assay

Cell proliferation was assessed with the CCK-8 assay. Pancreatic cancer cells in the logarithmic growth phase were trypsinized (G4001, Servicebio) and seeded into 96-well plates at a density of 1000 cells per well. Cells were incubated for 4 h. Five replicate wells were established for each experimental group. Subsequently, 10 μL of CCK-8 reagent (K1018, APExBIO) was added to each well, followed by a 2-h incubation period. The optical density (OD) at 450 nm was measured with a microplate reader (BioTek, Synergy H1). The cell proliferation levels among the groups were analysed with GraphPad software.

### 5-Ethynyl-2' -deoxyuridine (EdU) assay

Pancreatic cancer cells were seeded at an appropriate density into 24-well plates. Following cell attachment, EdU reagent (C0078S, Beyotime) was added, and the mixture was incubated for 2 h according to the manufacturer's protocol. The culture medium was subsequently removed, and the cells were fixed with 4% paraformaldehyde (P0099, Beyotime) for 15 min at room temperature. After three washes with phosphate buffered saline (PBS), the cells were permeabilized with Enhanced Immunostaining Permeabilization Buffer (P0097, Beyotime) for 10 min. Nuclear staining was performed with Hoechst 33,342. The cells were visualized and imaged under a microscope, with three random regions selected for counting. The cancer cell proliferation rate was determined as the percentage of EdU-positive cells relative to the total cell count.

### Transwell migration and matrix gel invasion assays

Pancreatic cancer cells were trypsinized, resuspended in serum-free medium, and counted. The cell density was adjusted to 1 × 10^5^ cells/mL. A 200 μL aliquot of the cell suspension was seeded into the upper chamber of a Transwell insert. For invasion assays, a layer of matrix gel (#356,234, Corning) was precoated on the upper chamber. The lower chamber was filled with 700 μL of medium containing 20% FBS. The Transwell chambers were incubated for 48 h. The chambers were subsequently washed twice with PBS, fixed in 4% paraformaldehyde for 10 min, and stained with 1% crystal violet (G1062, Solarbio) for 2–3 min. Nonmigrating or noninvading cells were removed from the upper chamber membrane with a medical swab. The cells on the lower membrane were visualized under an inverted microscope (Olympus, IX73). The number of migrated or invaded cells in five randomly selected fields of view was quantified.

### Flow cytometry

To assess apoptosis, cancer cells from distinct groups were collected and subjected to centrifugation at 800 rpm for 5 min. After two washes with PBS, the cells were incubated with apoptosis detection reagents according to the flow cytometry kit protocol (G1514, Servicebio) for 10 min in the dark. Apoptosis analysis was subsequently performed with flow cytometry (Agilent, NovoCyte Advanteon Dx VBR). For macrophage surface marker detection, the cells were incubated with anti-CD11b, anti-CD86, and anti-CD163 antibodies (301,306, 305,412, and 333,618 respectively; Biolegend) for 30 min in the dark, followed by two washes with cold PBS. Flow cytometry (Agilent, NovoCyte Advanteon Dx VBR) was used to quantify different TAM subpopulations.

#### RNA extraction, reverse transcription and real-time fluorescence quantitative PCR

Total RNA was isolated from cells with TRIzol reagent (9108, Takara) according to the manufacturer's protocol. cDNA was subsequently synthesized with the PrimeScript™ RT Reagent Kit (RR047A, Takara). Quantitative real-time PCR was performed to detect target gene expression with TB Green Premix Ex Taq (RR820A, Takara) on a Bio-Rad CFX96 system. The primer and probe sequences are provided in Table S2.

#### Protein extraction and gel electrophoresis

Total proteins were extracted from human tissue and cells with enhanced RIPA lysis buffer (AR0102-10, BOSTER) supplemented with protease inhibitors (AR1182-1, BOSTER) and phosphatase inhibitors (AR1183, BOSTER). The protein concentration was determined with a BCA protein assay kit (AR1189, BOSTER). Proteins were subsequently denatured by boiling and separated on 10% or 6% SDS-PAGE gels before being transferred to PVDF membranes. The membranes were blocked with 5% bovine serum albumin (GC305010-5 g, Servicebio) for one hour and incubated overnight at 4℃ with primary antibodies against MMP28 (1:500, sc-515010, Santa Cruz Biotechnology), GAPDH (1:1000, GB12002, Servicebio), β-actin (1:1000, GB15003-100, Servicebio), JNK (1:500, BM4329, BOSTER), p-JNK (1:500 BM4380, BOSTER), P38-MAPK (1:500, BM4439, BOSTER), p-P38-MAPK (1:500, M00176T180, BOSTER), ERK (1:500, BM3426, BOSTER), p-ERK (1:500, BM4156, BOSTER), ANXA2 (1:1000, 11256-1-AP, Proteintech), Vimentin (1:500, PB9359, BOSTER), CDH1 (1:500, BM4166, BOSTER), CDH2 (1:500, BA0673, BOSTER), BAX (1:500, BA0315-2, BOSTER), and BCL-2 (1:500, A00040-2, BOSTER). After three washes with TBST, the membranes were incubated with the appropriate concentration of secondary antibody for one hour at room temperature. Protein bands were visualized with a chemiScope imager (Clinx, chemiScope S6) after development with a supersensitive ECL luminescent solution (G2014, Servicebio).

#### Immunohistochemical staining

Tissues samples were fixed in 4% paraformaldehyde, embedded in paraffin, sectioned at a thickness of 5 μm, deparaffinized, and hydrated. Antigen retrieval was performed through two rounds of heat induced epitope retrieval with sodium citrate buffer. Endogenous peroxidase activity was quenched with 3% hydrogen peroxide, and nonspecific binding sites were blocked with 5% bovine serum albumin. Immunohistochemical staining was conducted by incubating the slides with primary antibodies (against MMP28 [1:100, sc-515010, Santa Cruz Biotechnology], CD86 [1:200, CQA1883, Cohesion Biosciences], CD163 [1:1000, 68,218–1-IG, Proteintech], and Ki67 [1:200, GB111141-100, Servicebio]) overnight at 4 °C, followed by incubation with the corresponding secondary antibody for 60 min at room temperature. Immunoreactivity was visualized with DAB chromogen, and the nuclei were counterstained with haematoxylin. Images were captured with a pathological section panoramic scanner (Science, Winmedic) and independently assessed by two researchers to determine the immunostaining intensity on the basis of the proportion of positively stained cells.

#### Preparation of conditioned medium (CM)

As previously described [15], the method of preparation of CM from AsPC-1 and BxPC-3 cancer cells was modified. Briefly, 1 × 10^6^ cells were seeded per well in a 6-well plate and cultured with serum-free medium for 48 h. The supernatant was subsequently collected by centrifugation at 3000 rpm for 5 min, followed by a final filtration step in which a 0.22 μm filter was used to obtain the CM.

#### In vitro migration of TAMs and polarization of M2 TAMs

To generate macrophages, THP-1 cells were differentiated into TAMs by treatment with 100 ng/mL phorbol 12-myristate 13-acetate (PMA) (CAS: 16,561–29-8, Sigma-Aldrich, St. Louis, MO, USA) for 48 h. TAM migration was assessed with a 24-well plate Transwell system equipped with 8 μm polycarbonate nucleopore filters (3422, Corning, NY, USA). Cancer cell conditioned medium supplemented with 10% FBS was added to the lower chamber, with or without neutralizing antibodies against IL-8 (MAB208-SP, R&D Systems, Minneapolis, MN, USA) and VEGFA (MAB293-SP, R&D Systems, Minneapolis, MN, USA). Macrophages (2 × 10^5^ cells/well) were seeded in the upper chamber. After 48 h, migrated TAMs were stained with 1% crystal violet, and images of five representative regions on each membrane were captured and quantified with ImageJ software. For M2 TAM polarization, a coculture system was used with a 6-well 0.4 μm polycarbonate membrane cell embedding dish (TCS001006, Biofil, Ann Arbor, MI, USA). Macrophages were seeded in the lower chamber, and cancer cells subjected to various treatments were added to the upper chamber (5 × 10^5^/well). Following 48 h of coculture, the cells from the lower chamber were collected for subsequent analysis.

#### Cytokine antibody array

A human cytokine antibody array (QAH-CYT-1, RayBiotech) was used in accordance with the manufacturer’s protocol to characterize the cytokine secretion profiles of AsPC-1 and BxPC-3 cells to identify cytokines exhibiting significantly divergent expression levels.

#### Enzyme-linked immunosorbent assay (ELISA)

Pancreatic cancer cell culture medium was subjected to contrifugation at 3000 rpm for 5 min to eliminate cellular debris. The levels of and then IL-8 and VEGFA secreted by the pancreatic cancer cells were subsequently quantified with ELISA kits procured from Elabscience (catalog numbers E-EL-H6008 and E-EL-H0111c), with strict adherence to the manufacturer's protocols. Data normalization was executed with Origin software.

#### RNA sequencing

Tumor cells subjected to different treatment regimens were lysed with TRIzol reagent (9108, Takara) and subsequently preserved in liquid nitrogen with triplicate samples per experimental group. RNA extraction was performed, followed by quality assessment with an Agilent 2100 bioanalyzer. cDNA library preparation and subsequent Illumina sequencing were outsourced to Genechem. Differential gene expression analysis was conducted with a significance threshold of *P* < 0.05 utilizing the 'limma' package within the R 3.6.1 statistical environment. Hierarchical clustering and functional enrichment analyses were implemented with the 'ClusterProfiler' package.

#### Assessment of interacting proteins

To investigate proteins that interacted with MMP28 in AsPC-1 and BxPC-3 cells, whole-cell lysates were prepared with enhanced RIPA lysis buffer supplemented with protease and phosphatase inhibitors. The protein concentration was determined via the BCA assay. Subsequently, 500 μL of lysate was incubated with either a primary MMP28 antibody or an IgG control under gentle agitation at 4 °C overnight for immunoprecipitation. Protein A/G agarose beads were then added to capture the antibody-protein complexes, followed by incubation under the same conditions for 3 h. Immunoprecipitates were collected by centrifugation at 2500 rpm for 5 min, and washed five times with RIPA buffer prior to downstream protein analysis.

#### Liquid Chromatograph Mass Spectrometer/Mass Spectrometer (LC–MS/MS)

Samples were subjected to analysis with an Easy-nLC1200 system coupled with a Q Exactive™ Hybrid Quadrupole-Orbitrap™ mass spectrometer. Prior to analysis, the samples were resuspended in 100 μL of 50 mM ammonium bicarbonate solution. Dithiothreitol (DDT) was subsequently added to a final concentration of 10 mM, and the mixture was reduced to 56 °C for one hour in a water bath. Subsequently, iodoacetamide was introduced to a final concentration of 50 mM, and the reaction was conducted in a lightproof environment for 40 min. Trypsin was then added for overnight digestion. Following digestion, the peptides were desalted with a self-filling desalting column, and the solvent was evaporated at 45℃ under vacuum. Liquid chromatography was performed on an Easy-nLC1200 system equipped with a C18 nanometre column (75 μm × 150 mm, 3 μm particle size, 100 Å pore size) at a flow rate of 600 nL/min. Mobile phase A consisted of 0.1% formic acid in water, and mobile phase B consisted of 0.1% formic acid in acetonitrile. A linear gradient was applied as follows: 4% B for 0–2 min, 4–28% B for 2–45 min, 28–40% B for 45–55 min, and 40–95% B for 55–66 min. Mass spectra were acquired in the Orbitrap analyser with a resolution of 70,000 for full MS scans (m/z 300–1800) and 17,500 for MS/MS scans. The automatic gain control was set to 3 × 10^6^ for full MS and 1 × 10^5^ for MS/MS. Database searching was conducted on intact tryptic peptides allowing for up to two missed cleavage sites. Carbamidomethylation of cysteine was specified as a fixed modification, whereas oxidation of methionine and protein N-terminal acetylation were considered variable modifications. The mass tolerance for peptide and fragment ions was set to 20 ppm. Peptide identification was validated at a false discovery rate of ≦1%.

#### Immunofluorescence staining assay

TAMs within the coculture system were fixed in 4% paraformaldehyde for 15 min, followed by permeabilization with Enhanced Immunostaining Permeabilization Buffer (P0097-100, Beyotime) for 10 min. After two washes with PBS, the cells were incubated with immunostaining blocking solution (P0102, Beyotime) for 30 min to reduce nonspecific antibody binding. Primary antibodies targeting CD86 and CD163 were subsequently applied and incubated overnight at 4℃. The cells were then incubated with fluorescent secondary antibodies (GB22303, GB21301, Servicebio) for 1 h to visualize protein expression. Nuclei were counterstained with DAPI (G1012-10ML, Servicebio), and the cellular samples were examined and imaged by fluorescence microscopy.

#### Coimmunoprecipitation (Co-IP)

Lysates of AsPC-1 and BxPC-3 cells (500 μg each) were coincubated with either anti-MMP28 or anti-ANXA2 antibodies (2 μg) for immunoprecipitation. Protein A/G agarose was added to the lysate-antibody mixture and incubated with gentle agitation at 4℃ for 3 h. The immunocomplexes were subsequently washed five times with RIPA lysis buffer, followed by precipitation with 20-40µL of 1 × SDS loading buffer. Proteins were denatured by heating and resolved by SDS-PAGE electrophoresis. Following transfer to a PVDF membrane, the proteins were probed with the corresponding primary antibody, and immunoreactive bands were visualized by chemiluminescence.

#### Statistics and analysis

Statistical analyses were performed with SPSS Statistics 26 and GraphPad Prism 8 software. The quantitative data are presented as the mean ± standard deviations. In vitro cell experiments were independently performed at least three times. Pairwise comparisons between groups were conducted with t tests whereas ANOVA was used for multigroup comparisons. The relationship between the expression of MMP28, CD86, and CD163 and clinicopathological features was assessed with the chi-square test. A *P*-value less than 0.05 was considered statistically significant.

## Results

### High expression of MMP28 in human pancreatic cancer is associated with the distribution of TAMs in clinical samples and with pancreatic cancer progression

**Fig. 1:**
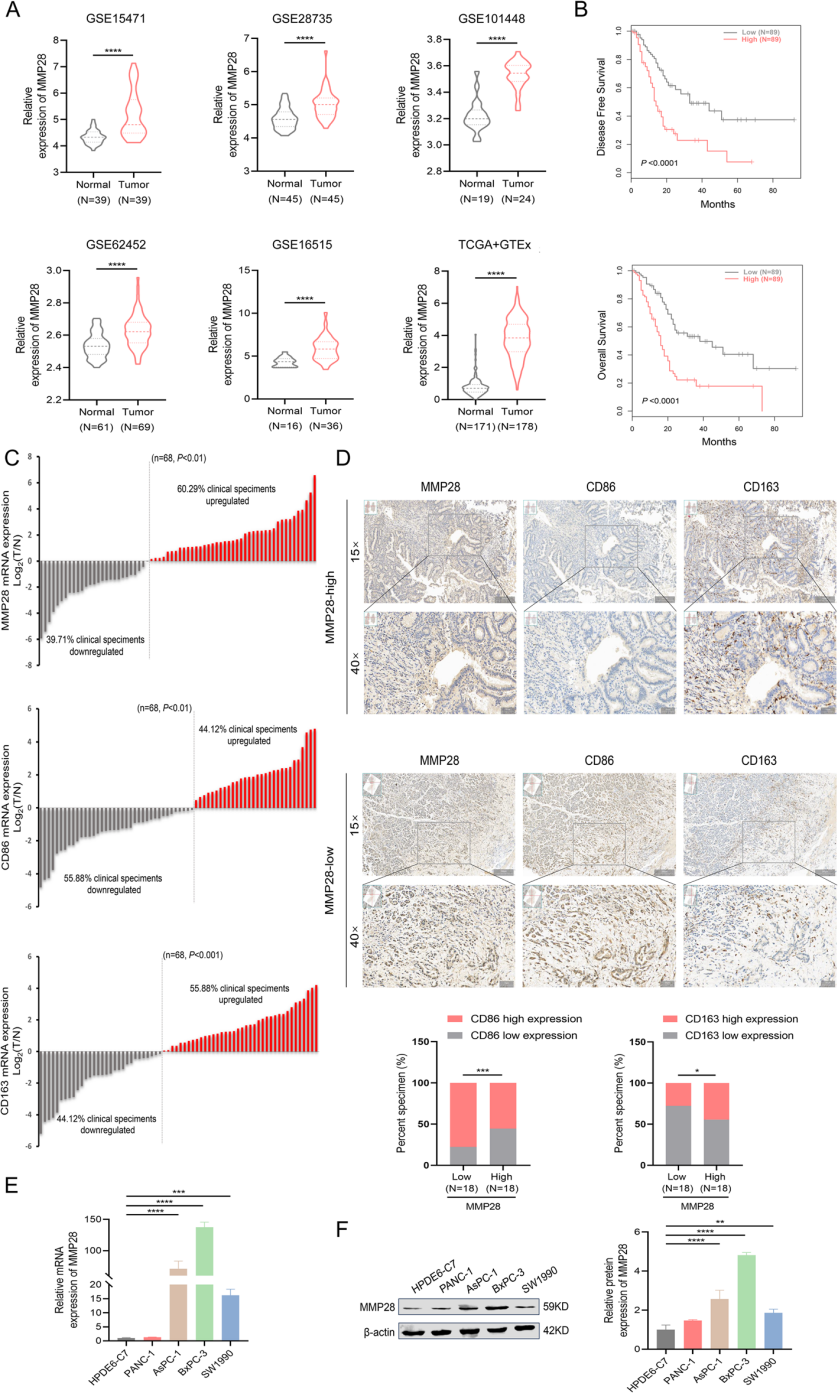
High expression of MMP28 in human pancreatic cancer is correlated with the distribution of TAMs in clinical samples and the progression of pancreatic cancer. **A** The expression levels of MMP28 in GSE15471, GSE28735, GSE101448, GSE62452, GSE16515, TCGA and UCSC in tumor tissues and normal nontumor tissues. **B** TCGA combined with GTEx was analysed for OS and DFS associated with MMP28 in pancreatic cancer patients. **C** The expression of MMP28 in 68 pairs of pancreatic cancer tissues and adjacent nontumor tissues was determined by qRT-PCR. **D** The protein expression of MMP28, CD86 and CD163 in human pancreatic cancer tissues was determined by IHC staining. Representative images of cancers with different MMP28 expression levels are shown (low expression; *N* = 18, high expression; *N* = 18). Scale bar, 50 μm. **E**–**F** The expression level of MMP28 in four pancreatic cancer cell lines was determined by qRT-PCR and Western blotting (WB). *P < 0.05, **P < 0.01, ***P < 0.001, and ****P < 0.0001

To elucidate the interplay between pancreatic cancer and the tumor microenvironment, pancreatic cancer samples and corresponding clinical data were procured from three publicly accessible databases (TCGA, UCSC, and GEO). Bioinformatics analysis revealed substantial upregulation of MMP28 in pancreatic cancer tissues compared with that in their noncancerous counterparts, which was concomitantly associated with an unfavorable prognosis (Fig. 1A-B). Subsequent qRT-PCR analysis of 68 pancreatic cancer and adjacent noncancerous tissues samples confirmed elevated MMP28 and CD163 mRNA levels, whereas CD86 mRNA expression was lower in cancer tissues than in noncancerous controls (Fig. 1C). To evaluate their clinical significance, the correlations between MMP28, CD86, and CD163 expression and patient clinicopathological characteristics were investigated. As depicted in Table 1, increased MMP28 expression, elevated CD163 + TAMs, and reduced CD86 + TAMs were significantly correlated with the pancreatic cancer site (*P* < 0.05). Moreover, MMP28 upregulation was associated with the advanced American Joint Committee on Cancer (AJCC) stage and lymph node metastasis (*P* < 0.05). Immunohistochemical staining of 36 randomly selected pancreatic cancer clinical samples revealed a positive correlation between MMP28 and CD163 (*P* < 0.05), but a negative correlation between MMP28 and CD86 (*P* < 0.001), suggesting a close association between MMP28 and CD163 + TAM infiltration (Fig. 1D). To further explore MMP28 expression, four pancreatic cancer cell lines were examined, revealing significant overexpression of MMP28 at both the mRNA and protein levels (*P* < 0.05) (Fig. 1E-F). Collectively, these findings indicate that MMP28 plays a critical role in pancreatic cancer progression and is intimately linked to tumor microenvironment remodeling.

**Table 1:** Association between clinicopathologic features with the expression levels of MMP28, CD86 and CD163

**Clinicopathological features**	**All cases** **N (%)**	**MMP28** **expression**			**CD86** **expression**			**CD163** **expression**		
		**Low ** **N (%)**	**High ** **N (%)**	***P*-value**	**Low ** **N (%)**	**High ** **N (%)**	***P*-value**	**Low ** **N (%)**	**High ** **N (%)**	***P*-value**
Age (years)				0.657			0.502			0.748
<60	28(41.2)	12(44.4)	16(39.0)		17(44.7)	11(36.7)		13(43.3)	15(39.5)	
≥60	40(58.8)	15(55.6)	25(61.0)		21(55.3)	19(63.3)		17(56.7)	23(60.5)	
Gender				0.784			0.833			0.417
Male	44(64.7)	18(66.7)	26(63.4)		25(65.8)	19(63.3)		21(70.0)	23(60.5)	
Female	24(35.3)	9(33.3)	15(36.6)		13(34.2)	11(36.7)		9(30.0)	15(39.5)	
Primary site				0.025*			0.035*			0.018*
Pancreatic head	56(82.4)	19(70.4)	37(90.2)		28(73.7)	28(93.3)		21(70.0)	35(92.1)	
Pancreatic body tail	12(17.6)	8(29.6)	4(9.8)		10(26.3)	2(6.7)		9(30.0)	3(7.9)	
AJCC stage				0.038*			0.273			0.776
I-II	58(85.3)	26(96.3)	32(78.0)		34(89.5)	24(80.0)		26(86.7)	32(84.2)	
III-IV	10(14.7)	1(3.7)	9(22.0)		4(10.5)	6(20.0)		4(13.3)	6(15.8)	
Tumor size (cm)				0.914			0.742			0.620
<2	9(13.2)	3(11.1)	6(14.6)		4(10.5)	5(16.7)		3(10.0)	6(15.8)	
2-4	44(64.7)	18(66.7)	26(63.4)		25(65.8)	19(63.3)		19(63.3)	25(65.8)	
>4	15(22.1)	6(22.2)	9(22.0)		9(23.7)	6(20.0)		8(26.7)	7(18.4)	
Vascular invasion				0.147			0.499			0.492
Yes	22(32.4)	6(22.2)	16(39.0)		11(28.9)	11(36.7)		12(40.0)	10(26.3)	
No	46(67.6)	21(77.8)	25(61.0)		27(71.1)	19(63.3)		21(60.0)	25(73.7)	
Nerve invasion				0.474			0.496			0.398
Yes	51(75.0)	19(70.4)	32(78.0)		28(73.7)	23(76.7)		24(80.0)	27(71.2)	
No	17(25.0)	8(29.6)	9(22.0)		10(26.3)	7(23.3)		6(20.0)	11(28.8)	
LN metastasis				0.026*			0.329			0.625
Positive	34(50.0)	9(33.3)	25(61.0)		17(44.7)	17(56.7)		14(46.7)	20(52.6)	
Negative	34(50.0)	18(66.7)	16(39.0)		21(55.3)	13(43.3)		16(53.3)	18(47.4)

### MMP28 promotes the proliferation and invasion of pancreatic cancer cells in vitro

**Fig. 2:**
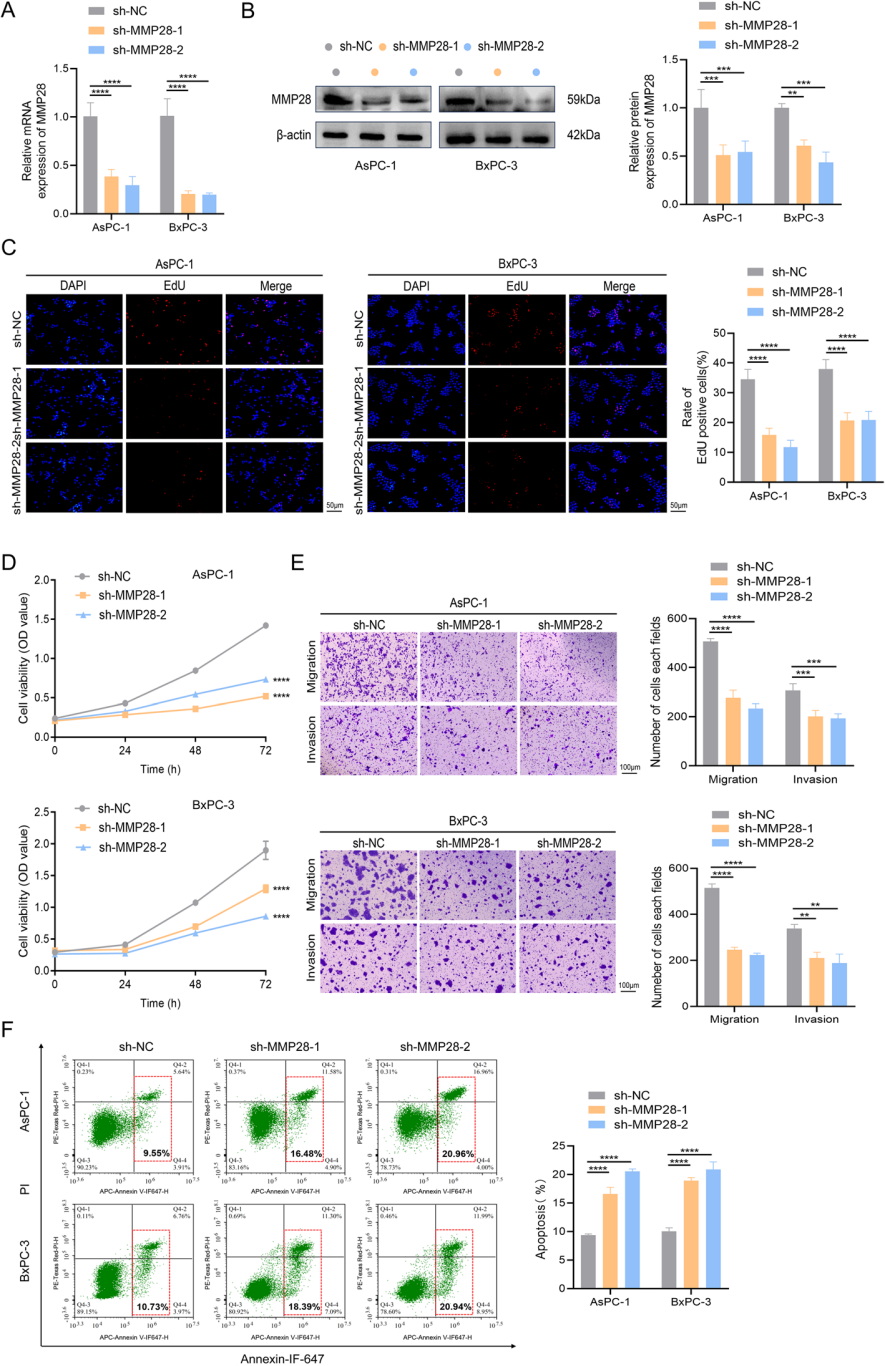
Effect of the inhibition of MMP28 expression on the proliferation, migration and invasion of pancreatic cancer cells. **A**-**B** Empty lentivirus (sh-NC) and MMP28 knockdown lentivirus (sh-MMP28) were transfected into AsPC-1 and BxPC-3 cells, and the knockdown efficiency of MMP28 was verified by qRT-PCR and WB. **C** The proliferation ability of the indicated cancer cells was determined with an EdU kit. Scale bar, 50 μm. **D** CCK-8 reagent was used to assess the proliferation ability of the above cancer cells. **E** Transwell assays were used to assess the migration and invasion ability of cancer cells. Scale bar, 100 μm. **F** Flow cytometry was used to analyse the degree of cancer cell apoptosis in the two cell types. **P < 0.01, ***P < 0.001, and ****P < 0.0001

To further elucidate the role and function of MMP28 in pancreatic cancer, AsPC-1 and BxPC-3 cell lines were selected for in-depth investigation on the basis of the preceding findings. Lentiviral transfection was used to modulate the MMP28 expression levels in both cell lines, resulting in alterations in the MMP28 mRNA and protein levels (Fig. 2A-B, Fig. S1A-B). EdU staining and CCK-8 assays demonstrated a decrease in cell proliferation following MMP28 knockdown and a significant increase in proliferation upon MMP28 overexpression in both pancreatic cancer cell lines (Fig. 2C-D, Fig. S1C-D). Transwell assays revealed that MMP28 overexpression increased, whereas MMP28 inhibition suppressed, the migratory and invasive capacities of AsPC-1 and BxPC-3 cells (Fig. 2E, Fig. S1E). Additionally, assessment of cell apoptosis revealed an increase in apoptosis upon MMP28 knockdown and a significant decrease upon MMP28 overexpression in both cancer cell lines (Fig. 2F, Fig. S1F). Western blot analysis was conducted to investigate the effects of MMP28 on EMT and apoptosis. Compared with the control, MMP28 knockdown upregulated BAX (apoptosis) and CDH1 (EMT) expression, whereas MMP28 overexpression promoted BCL2 (apoptosis), CDH2, and Vimentin expression (EMT) (Fig. S2A-2B). In conclusion, these findings collectively suggest that MMP28 promotes pancreatic cancer cell proliferation, migration, and invasion and inhibits apoptosis, thereby contributing to promoting malignant progression.

### Knockdown of MMP28 inhibits the growth of pancreatic cancer cells and the infiltration of M2 TAMs in vivo

**Fig. 3:**
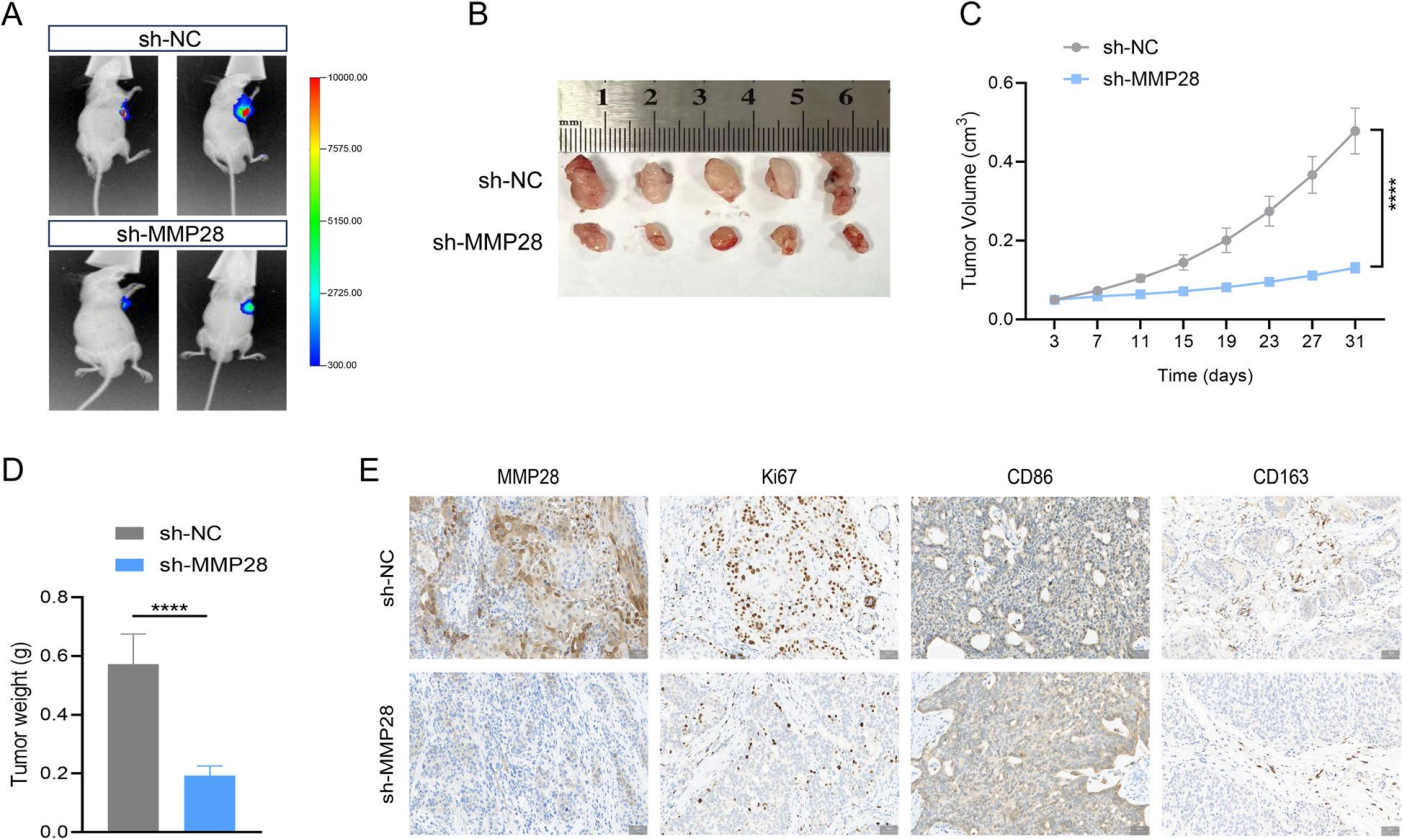
Knockdown of MMP28 inhibits the growth of pancreatic cancer growth and infiltration of M2 TAMs in vivo. **A** Representative images of subcutaneous tumors detected by fluorescein potassium salt in nude mice. BxPC-3 cells were stably transfected with the MMP28 knockdown construct (sh-MMP28) or control vector (sh-NC) and injected subcutaneously into nude mice. **B** Representative images of subcutaneous xenograft tumors (5 mice per group). **C** The volume of the tumor was measured every four days. **D** Body weights of the tumors in the two groups. **E** Representative images of the expression of MMP28 and Ki67 and the infiltration of TAMs in the xenograft tumors of the two groups determined by immunohistochemical staining. Scale bar, 50 μm

To further investigate the in vivo impact of MMP28 on pancreatic cancer progression and TAM infiltration, BxPC-3 cells with MMP28 knockdown (sh-MMP28) and control BxPC-3 cells (sh-NC) were subcutaneously injected into BALB/c nude mice to establish pancreatic cancer xenograft models. Consistent with expectations, in vivo bioluminescence experiments revealed significant inhibition of tumor growth in the MMP28 knockdown group compared with the control group (Fig. 3A-B). Moreover, regular measurements revealed a marked reduction in tumor volume and body weight in the knockdown group relative to those in the control group (Fig. 3C-D). Immunohistochemical staining of tumor tissues from both groups revealed significantly lower MMP28 and Ki67 expression levels in the knockdown group than in the control group. Notably, the infiltration of CD163 + TAMs was significantly decreased, whereas the infiltration of CD86 + TAMs was significantly increased in the knockdown group compared with the control group (Fig. 3E). These findings collectively suggest that MMP28 promotes pancreatic cancer growth and M2 TAM infiltration in vivo, further corroborating its role in pancreatic cancer pathogenesis.

### In vitro high expression of MMP28 in cancer cells promotes the migration of TAMs and the polarization of M2 TAMs

**Fig. 4:**
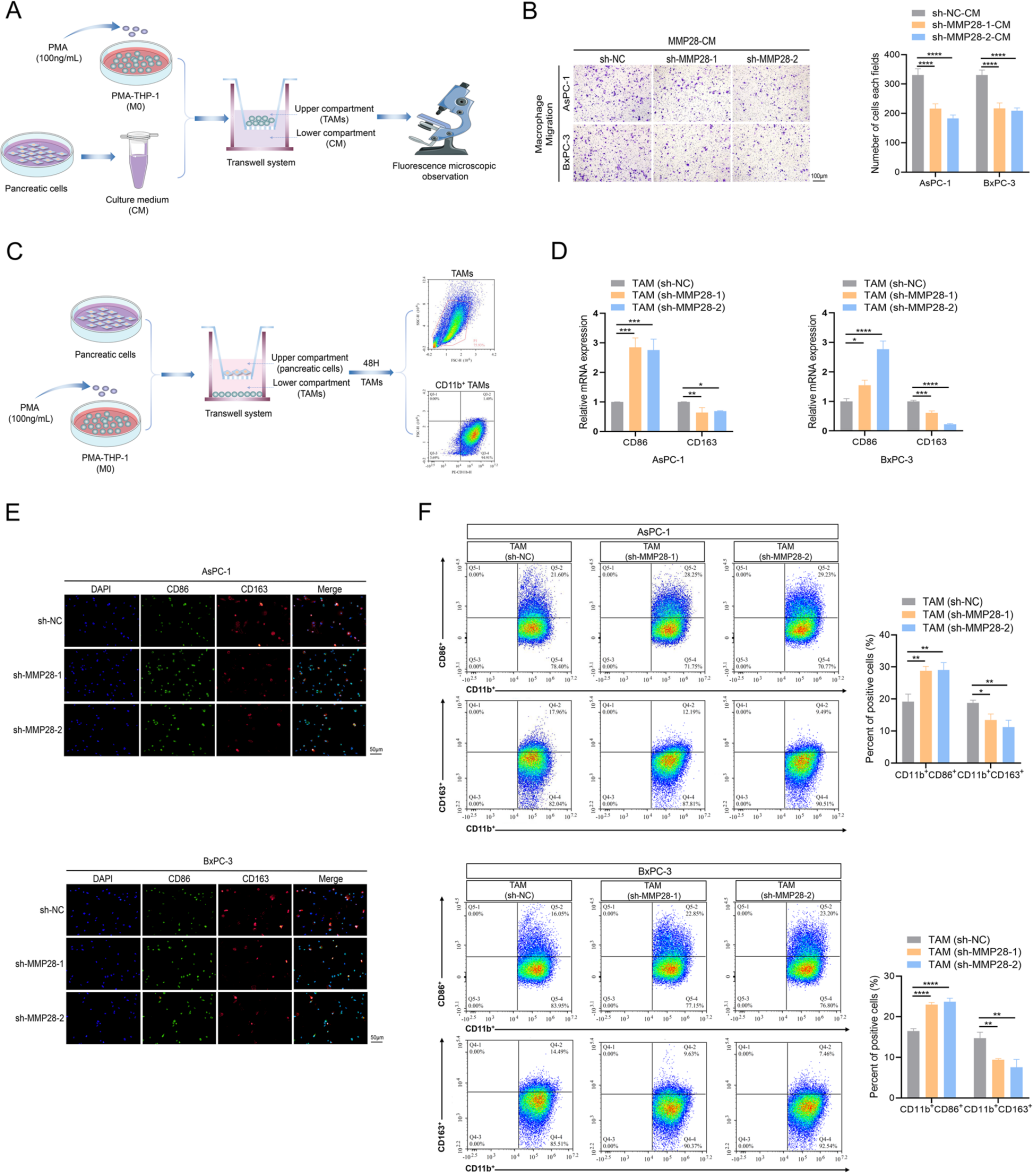
Knockdown of MMP28 inhibits the migration of TAMs and the polarization of M2 TAMs. **A** Schematic diagram of TAM migration induced by pancreatic cancer cells. The lower chamber contained CM from cancer cells in different treatment groups, and the upper chamber contained TAMs. **B** The ability of the sh-NC group and the sh-MMP28 group to attract TAMs was determined in 8 μm Transwell chambers. Scale bar, 100 μm. **C** Schematic diagram of TAM polarization induced by pancreatic cancer cells. The lower chamber contained TAMs, and the upper chamber contained cancer cells from different treatment groups (0.4 μm Transwell chambers). CD11b + TAMs were isolated by flow cytometry for subsequent assessment. **D** The expression levels of the TAM markers CD86 and CD163 in different coculture groups were determined by qRT-PCR. **E** The expression levels of the TAM markers CD86 and CD163 in different coculture groups were determined by immunofluorescence staining assay. Scale bar, 50 μm. **F** The proportions of CD11b + CD86 + TAMs and CD11b + CD163 + TAMs in different coculture groups were analysed by flow cytometry. *P < 0.05, **P < 0.01, ***P < 0.001, and ****P < 0.0001

Our previous investigations demonstrated that MMP28 knockdown attenuates the influence of tumor tissue on M2 TAM infiltration in vivo. To further elucidate the underlying mechanisms of the cancer cell-TAM interaction, an in vitro coculture system was established. As shown in Fig. 4A, CM from cancer cells was added to the lower chamber of the coculture system, while PMA-induced TAMs were placed in the upper chamber. After a 48-h incubation period, cancer cell CM (from AsPC-1 and BxPC-3 cell lines) with MMP28 knockdown exhibited a reduced capacity to attract TAMs compared with that of the control group. Conversely, CM derived from cancer cells overexpressing MMP28 demonstrated a significantly increased ability to recruit TAMs (Fig. 4B, Fig. S3A). To assess the impact of cancer cells on TAM polarization, the coculture system was refined. Induced TAMs were positioned in the lower chamber, while cancer cells from different groups were cultured in the upper chamber for 48 h (Fig. 4C). qRT-PCR analysis revealed that TAMs cocultured with MMP28-knockdown cancer cells presented upregulated CD86 expression and downregulated CD163 expression. In contrast, TAMs cocultured with MMP28-overexpressing cancer cells displayed the opposite expression pattern (Fig. 4D, Fig. S3B). Immunofluorescence staining analysis of TAMs from the aforementioned groups corroborated these findings, demonstrating a propensity for CD86 expression in TAMs cocultured with the knockdown group and CD163 expression in TAMs cocultured with the overexpression group (Fig. 4E, Fig.S3C). Flow cytometry was used to further validate these results. TAMs were initially identified within the coculture system with the CD11b marker, followed by evaluation of CD86 and CD163 expression in CD11b + TAMs (Fig. 4C). Consistent with previous observations, MMP28 overexpression significantly promoted CD163 expression in CD11b + TAMs, whereas reduced MMP28 expression facilitated CD86 expression in CD11b + TAMs (Fig. 4F, SFig. 3D). Collectively, these findings unequivocally support the pivotal role of MMP28 in inducing TAM migration and promoting M2 TAM polarization in pancreatic cancer cells.

### MMP28 promotes the migration of TAMs and the polarization of M2 TAMs by the secretion of multiple cytokines

**Fig. 5:**
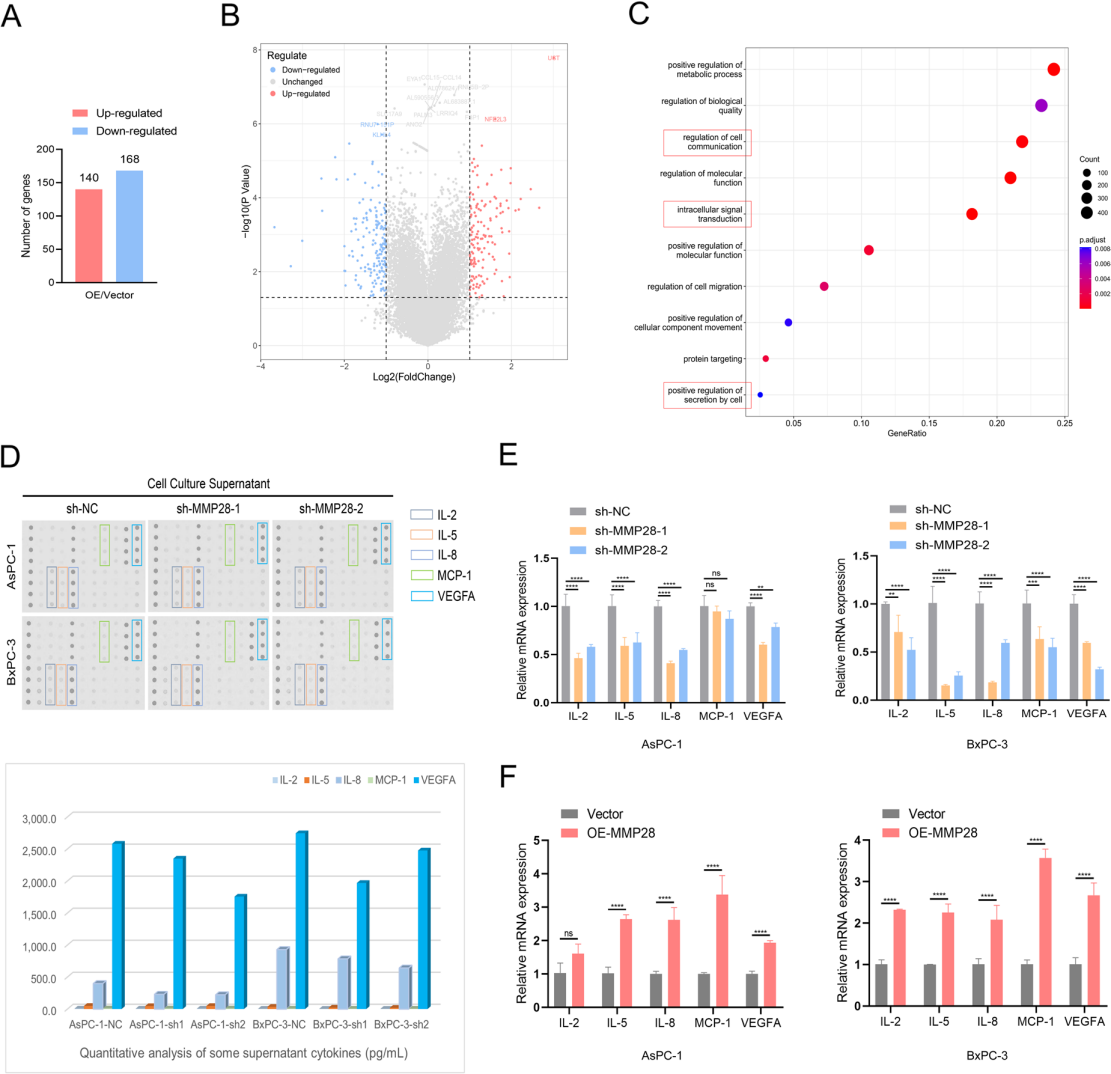
MMP28 promotes the secretion of IL-8 and VEGFA by cancer cells. **A** RNA sequencing was performed to analyse the differentially expressed genes, including 140 upregulated genes and 168 downregulated genes, in cancer cells overexpressing MMP28 and those in the control group. These differentially expressed genes were subsequently mapped in a volcano plot (**B**) and subjected to GO enrichment analysis (**C**). **D** Cytokine antibody array of the supernatants of sh-NC- and sh-MMP28-transfected cancer cells, in which IL-2, IL-5, IL-8, MCP-1, and VEGFA were downregulated. **E-F** qRT-PCR was used to analyse the effects of MMP28 on the expression of these cytokines in the two cancer cell lines. **P < 0.01, ***P < 0.001, and ****P < 0.0001

To comprehensively elucidate the functional roles of MMP28 in pancreatic cancer and the tumor microenvironment, RNA sequencing was performed to identify genes that were differentially expressed between MMP28 overexpressing and control AsPC-1 cells. When the selection criteria of |LogFC|> 1 and *P* < 0.05 were applied, a total of 140 upregulated and 168 downregulated genes were identified (Fig. 5A). Volcano plot and Gene Ontology (GO) functional enrichment analyses revealed that these differentially expressed genes were involved primarily in cell communication regulation, intracellular signal transduction, and positive regulation of cellular secretion (Fig. 5B-C). To further investigate the impact of MMP28 on cytokine secretion by pancreatic cancer cells, a Raybio human cytokine antibody array was used. As depicted in Fig. 5D, the secretion of IL-2, IL-5, IL-8, MCP-1, and VEGFA was significantly reduced in both the sh-MMP28-1 and sh-MMP28-2 AsPC-1 and BxPC-3 cancer cell lines. qRT-PCR analysis confirmed these findings, revealing decreased expression levels of IL-2, IL-5, IL-8, MCP-1, and VEGFA in MMP28-knockdown AsPC-1 and BxPC-3 cells, whereas the overexpression of MMP28 resulted in increased expression of these cytokines (Fig. 5E-F). Among these cytokines, IL-8 and VEGFA exhibited the most pronounced effects on extracellular secretion mediated by MMP28. Therefore, the effects of IL-8 and VEGFA on TAM recruitment and polarization were the primary focus of subsequent investigations.

**Fig. 6:**
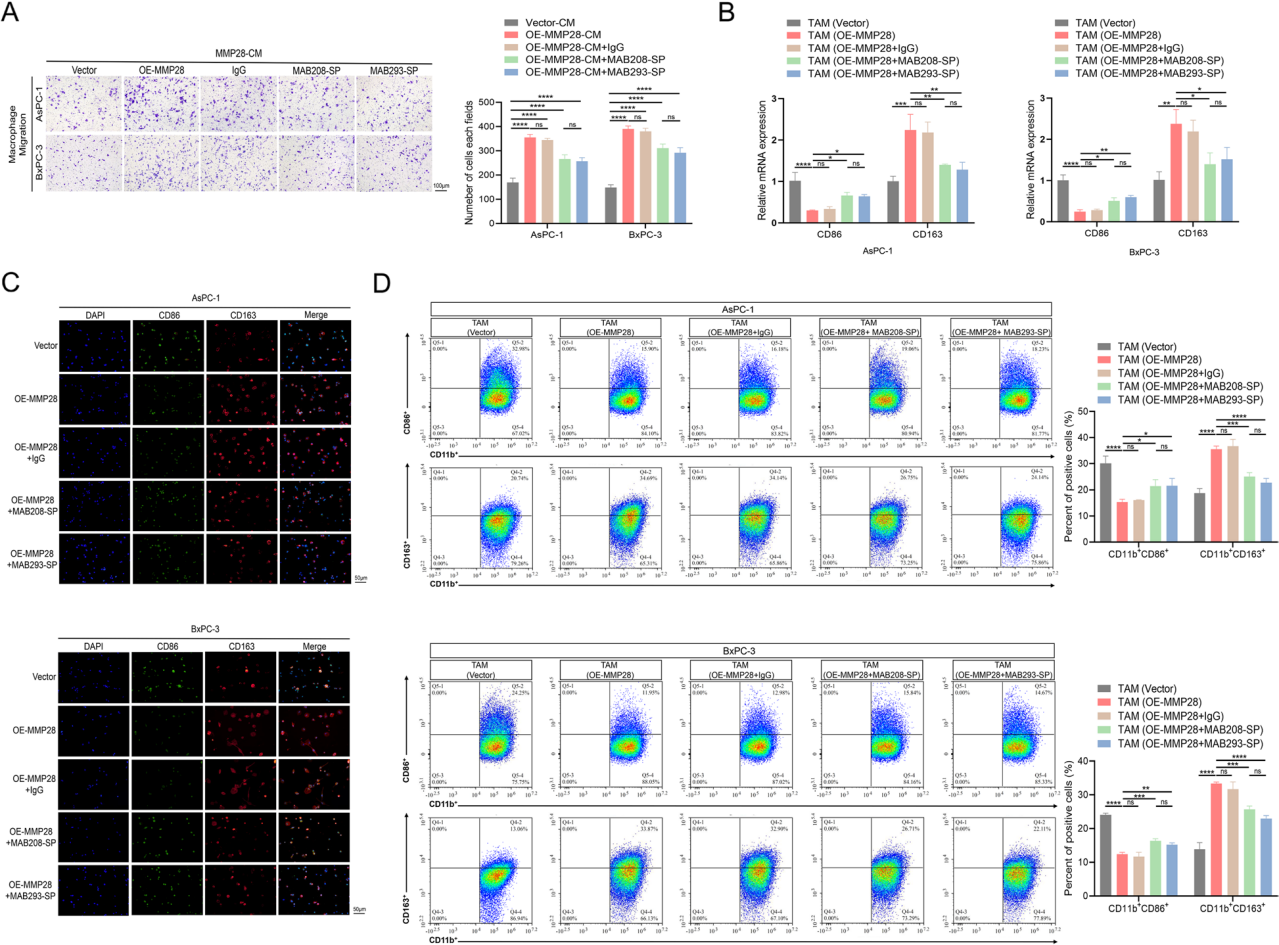
Anti-IL-8 and anti-VEGFA antibodies partially reverse the ability of MMP28 to promote the migration of TAMs and the polarization of M2 TAMs. **A** CM from MMP28-overexpressing pancreatic cancer cells was treated with an anti-IL-8 neutralizing antibody (5 μg/mL) or an anti-VEGFA neutralizing antibody (1 μg/mL). The migration level of TAMs was determined by the Transwell migration experiment. Scale bar, 100 μm. **B** qRT-PCR determination of CD86 and CD163 mRNA expression levels in TAMs cocultured with Vector group of pancreatic cancer cells transfected with empty vector virus, TAMs cocultured with OE-MMP28 cell group, TAMs cocultured with the OE-MMP28 + IgG cell group, TAMs cocultured with OE-MMP28 + Anti-IL-8 cell group, and TAMs cocultured with OE-MMP28 + Anti-VEGFA cell group. **C** The expression levels of the TAM markers CD86 and CD163 in different coculture groups were determined by immunofluorescence staining. Scale bar, 50 μm. **D** The proportions of CD11b + CD86 + TAMs and CD11b + CD163 + TAMs in different coculture groups were analysed by flow cytometry. *P < 0.05, **P < 0.01, ***P < 0.001, and ****P < 0.0001

Next, IL-8 neutralizing antibody (5 μg/mL) and VEGFA neutralizing antibody (1 μg/mL) were added to the CM of AsPC-1 and BxPC-3 cells. The results indicated that the effect of neutralization of IL-8 or VEGFA partially attenuated the MMP28-mediated recruitment of TAMs (Fig. 6A). To further investigate the potential involvement of IL-8 and VEGFA in MMP28-induced M2 TAM polarization, the effects of IL-8 and VEGFA neutralization on CD86 and CD163 expression levels in TAMs were assessed after 48 h of coculture. As depicted in Fig. 6B, neutralization of IL-8 or VEGFA partially reversed the MMP28-induced downregulation of CD86 and upregulation of CD163 in TAMs. Consistent with these findings, both immunofluorescence staining and flow cytometry analyses demonstrated that the addition of IL-8 and VEGFA-neutralizing antibodies to the CM of AsPC-1 and BxPC-3 cells partially mitigated the MMP28-induced alterations in CD86 and CD163 expression levels in TAMs (Fig. 6C-D). Collectively, these data suggest that MMP28 promotes TAM recruitment and M2 TAM polarization by influencing the secretion of IL-8 and VEGFA by AsPC-1 and BxPC-3 cells.

### MMP28 promotes IL-8 and VEGFA secretion by inducing phosphorylation of the MAPK/JNK signaling pathway

**Fig. 7:**
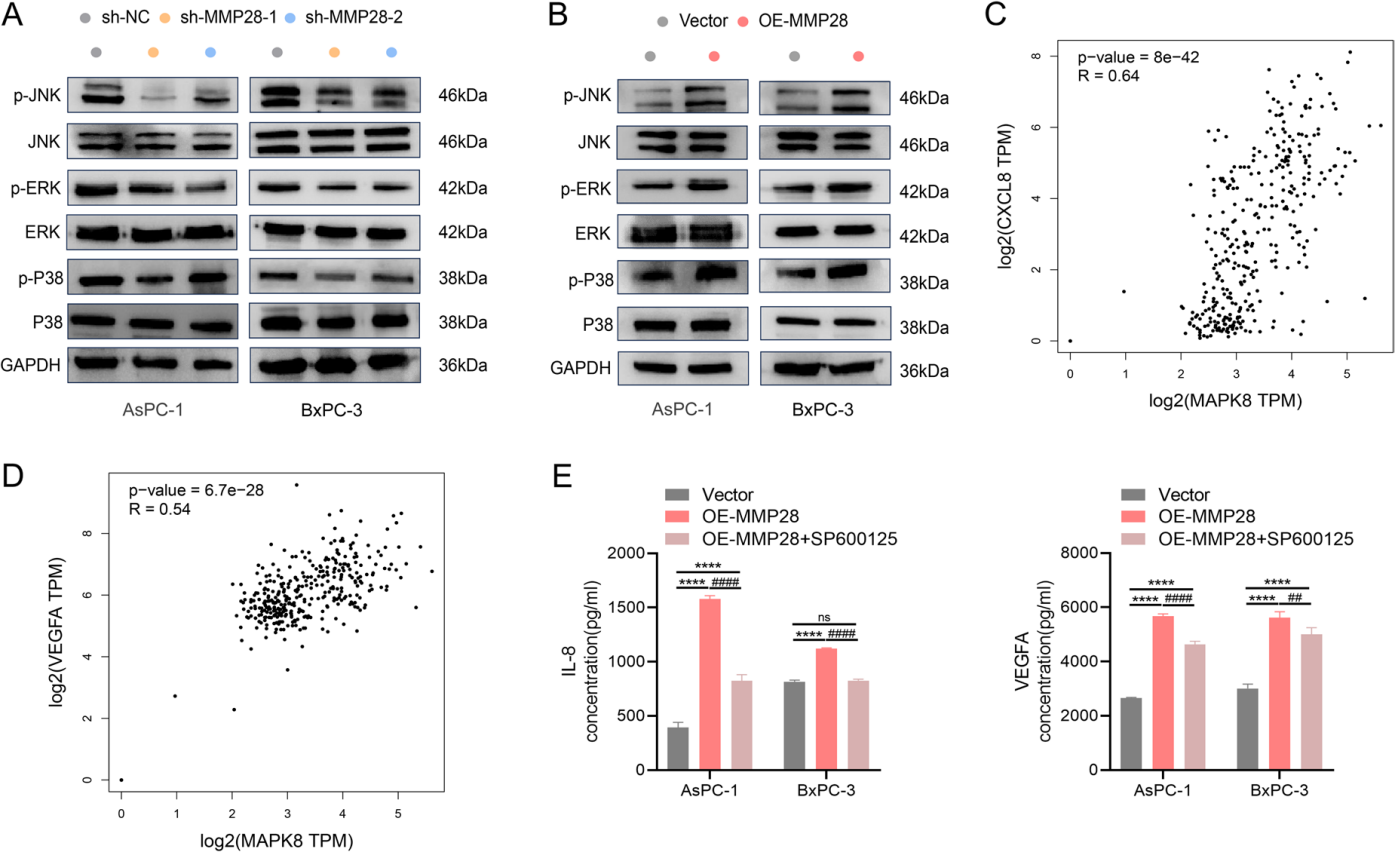
MMP28 promotes the secretion of IL-8 and VEGFA from cancer cells by regulating the MAPK/JNK signaling pathway. **A**-**B** WB analysis of JNK, p-JNK, ERK, p-ERK, P38, and p-P38 in pancreatic cancer cells with MMP28 knockdown or overexpression compared with the control. **C**-**D** GEPIA database analysis of the relationships between JNK expression and the expression of IL-8 and VEGFA in pancreatic cancer. **E** ELISAs were used to measure IL-8 and VEGFA protein secretion in empty vector-treated pancreatic cancer cells, MMP28-overexpressing pancreatic cancer cells, and pancreatic cancer cells treated with the JNK inhibitor SP600125 (20 μM). ****P < 0.0001. ###*P* < 0.01, and ####*P* < 0.0001

To further explore the signaling pathways mediating the role of MMP28 in cytokine secretion by pancreatic cancer cells, we investigated MAPK pathways known to be involved in cell migration and cytokine regulation [16, 17]. As shown in Fig. 7A-B, the knockdown or overexpression of MMP28 altered the activity of the P38, ERK1/2, and JNK signaling pathways in the two cancer cell lines. Notably, the phosphorylation pattern of JNK mirrored that of MMP28. To investigate the potential link between JNK and IL-8/VEGFA expression in pancreatic cancer, we analysed the GEPIA database (http://gepia.cancer-pku.cn/). The results revealed a positive correlation between JNK expression and both IL-8 and VEGFA (Fig. 7C-D). To validate the role of the MAPK/JNK pathway in MMP28-mediated IL-8/VEGFA secretion, we used SP600125 (20 μM), a specific JNK inhibitor, known to suppress JNK phosphorylation [17]. Following JNK inhibition, we measured the secretion of IL-8 and VEGFA in AsPC-1 and BxPC-3 cell supernatants. Notably, JNK inhibition partially reversed the stimulatory effect of MMP28 on IL-8 and VEGFA secretion by these cancer cells (Fig. 7E).

To investigate the impact of JNK inhibition on TAM behavior, the MAPK/JNK signaling pathway was blocked. Subsequent analysis revealed a significant attenuation of TAM migratory capacity in the MMP28-overexpressing group treated with SP600125 (a JNK inhibitor) compared with the group overexpressing MMP28 alone (Fig. S3A). Furthermore, qRT-PCR, immunofluorescence staining, and flow cytometry analyses demonstrated that JNK inhibition partially reversed the M2 TAM polarization induced by MMP28 overexpression (Fig.S3B-D). Collectively, these findings suggest that MMP28 promotes IL-8 and VEGFA secretion through activation of the MAPK/JNK signaling pathway, ultimately influencing TAM recruitment and M2 TAM polarization.

### ANXA2 increases the ability of MMP28 to promote the migration of TAMs and the polarization of M2 TAMs

**Fig. 8:**
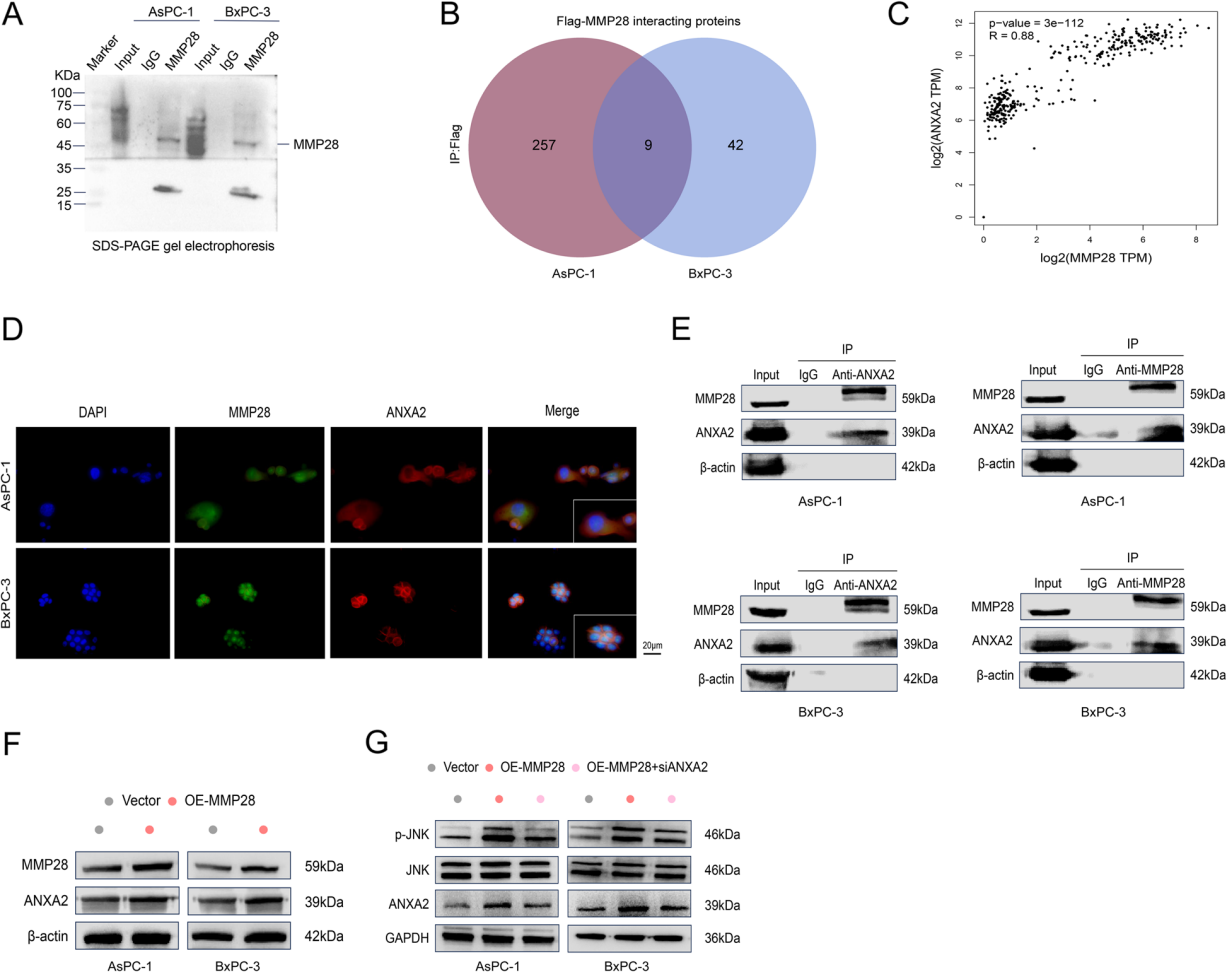
Physical interaction between MMP28 and ANXA2. **A** WB analysis of MMP28 interacting proteins pulled down using MMP28 antibody by co-IP in both AsPC-1 and BxPC-3 cells. **B** Analysis of proteins that interact with MMP28 and their overlap in AsPC-1 and BxPC-3 cells by a Q Exactive mass spectrometer. **C** A positive correlation between MMP28 expression and ANXA2 in pancreatic cancer was identified in the GEPIA database. **D** Immunofluorescence colocalization of MMP28 and ANXA2 in AsPC-1 and BxPC-3 cells was determined by microscopy to validate the physical interactions between the two proteins. Scale bar, 20 μm. **E** Interactions between endogenous MMP28 and ANXA2 in AsPC-1 and BxPC-3 cells. The cell lysates were collected and Co-IP and Western blot analyses were performed with antibodies against MMP28 and ANXA2, respectively. **F** Changes in ANXA2 expression levels in pancreatic cancer cell lines overexpressing MMP28. **G** Effect of ANXA2 knockdown on JNK phosphorylation in pancreatic cancer cell lines overexpressing MMP28

To elucidate the mechanisms underlying MMP28-mediated TAM migration and M2 TAM polarization, Co-IP was performed to confirm the enrichment of MMP28 and its interacting proteins in AsPC-1 and BxPC-3 cells (Fig. 8A). Subsequent LC–MS/MS analysis identified the top 30 candidate MMP28-interacting proteins in each cell line (Tables 2 and 3). Overlapping interacting proteins between the two cell lines were identified (Fig. 8B). Given the findings of the previous functional studies and gene expression data, ANXA2 emerged as a potential candidate influencing TAM polarization [18]. The GEPIA database confirmed a strong association between MMP28 and ANXA2 expression in pancreatic cancer (Fig. 8C). To further characterize the relationship between MMP28 and ANXA2, immunofluorescence staining analysis was performed, which revealed the colocalization of MMP28 (green) and ANXA2 (red) in AsPC-1 and BxPC-3 cells (Fig. 8D). Moreover, Co-IP experiments validated the physical interaction between these two proteins (Fig. 8E). Furthermore, ANXA2 expression was also increased in MMP28-overexpressing pancreatic cancer cells, and ANXA2 knockdown partially inhibited the ability of MMP28 to promote JNK phosphorylation (Fig. 8F-G).

**Table 2:** Top 30 candidates of Flag-MMP28 interacting proteins in AsPC-1 cells

**Protein name**	**Accession**	**MW (KDa)**	**Peptides**	**Coverage (%)**	**Score**	**Subcellular localization**
ANXA2	P07355	39	9	31.3	199.81	Plasma membrane
ERH	P84090	12	1	10.6	102.39	Nucleus
HSPA8	P11142	71	4	12.2	100.35	Nucleus
TPI1	P60174	27	5	33.7	94.829	Nucleus
TKT	P29401	68	10	21.8	76.273	Nucleus/cytosol
IGKV4-1	P06312	13	1	7.4	74.826	Plasma membrane
ENO1	P06733	47	8	25.8	66.799	Nucleus/cytosol
PKM	P14618	58	8	21.3	59.25	Mitochondrion
YWHAZ	P63104	28	7	30.2	58.514	Cytosol/nucleus
TUBA1B	P68363	50	7	23.5	57.653	Cytoskeleton
PPIA	P62937	18	4	27.9	53.218	Nucleus/cytosol
FLNB	O75369	278	7	3.6	50.63	Plasma membrane
HSP90AB1	P08238	83	1	11.9	50.308	Nucleus
ACTN4	O43707	105	7	9.1	49.183	Cytosol
GPI	P06744	63	5	12.2	47.731	Nucleus/cytosol
LDHA	P00338	37	5	16.3	47.155	Nucleus/cytosol
VIM	P08670	54	6	13.1	41.732	Cytosol
MIF	P14174	12	1	7.8	38.606	Plasma membrane
ACTG1	P63261	42	5	16.8	34.65	Cytoskeleton
HIST1H4A	P62805	11	3	27.2	32.581	Nucleus
PEBP1	P30086	21	2	18.7	30.628	Nucleus/cytosol
YWHAE	P62258	29	3	16.5	29.508	Nucleus/cytosol
PGK1	P00558	45	2	8.2	29.374	Cytosol
GAPDH	P04406	36	3	11	28.868	Nucleus/cytosol
LMNA	P02545	74	4	6.9	28.177	Cytoskeleton/nucleus
YWHAQ	P27348	28	4	24.5	28.07	Cytosol
MDH2	P40926	36	4	18.3	27.996	Mitochondrion
PRDX6	P30041	25	4	20.1	27.907	Nucleus/cytosol
HSPD1	P10809	61	3	8.2	26.852	Mitochondrion
TUFM	P49411	50	3	8.4	25.318	Mitochondrion

**Table 3:** Top 30 candidates of Flag-MMP28 interacting proteins in BxPC-3 cells

**Protein name**	**Accession**	**MW (KDa)**	**Peptides**	**Coverage (%)**	**Score**	**Subcellular localization**
TUBA1C	Q9BQE3	50	3	9.8	38.289	Nucleus/cytoskeleton
PKM	P14618	58	2	4.9	32.324	Mitochondrion
GAPDH	P04406	36	1	4.2	16.256	Nucleus/cytosol
TKT	P29401	68	1	3	16.206	Nucleus/cytosol
PCLO	Q9Y6V0	561	1	0.2	13.666	Cytoskeleton
LDHA	P00338	37	1	4.5	11.303	Nucleus/cytosol
RPLP2	P05387	12	1	10.4	10.894	Cytosol
TLR7	Q9NYK1	121	1	0.8	9.7321	Lysosome/endosome
DMPK	Q09013	69	1	5.4	8.9338	Mitochondrion/cytosol
NDNL2	Q96MG7	34	1	10.5	8.7755	Nucleus
RAB11FIP3	O75154	82	1	5.3	8.493	Nucleus/cytoskeleton
GOT2	P00505	48	1	2.8	8.085	Mitochondrion
FLNC	Q14315	291	1	0.6	7.8628	Plasma membrane
HNRNPA2B1	P22626	37	1	2.8	7.6581	Nucleus
GSTP1	P09211	23	1	6.2	7.5387	Mitochondrion/cytosol
ENO1	P06733	47	1	4.4	7.0666	Nucleus/cytosol
C6orf15	Q6UXA7	34	1	7.1	6.4583	Nucleus
AP4S1	Q9Y587	17	1	14.6	6.45	Endosome/golgi
RGS22	Q8NE09	147	1	1.7	6.45	Nucleus
SLC12A2	P55011	131	1	1.3	6.3897	Plasma membrane
ANXA2	P07355	39	1	4.7	6.2771	Plasma membrane
TTN	Q8WZ42	3816	1	0.1	6.1883	Nucleus/cytoskeleton
CCDC129	Q6ZRS4	115	1	4.2	6.1668	Nucleus
CCBL1	Q16773	48	1	5	6.1651	Cytosol
FEM1A	Q9BSK4	74	1	3.4	6.1586	Mitochondrion
MUC19	Q7Z5P9	805	1	0.5	6.1545	Plasma membrane
NUPL1	Q9BVL2	61	1	5.2	6.151	Plasma membrane
TLN1	Q9Y490	270	1	0.8	6.1244	Cytoskeleton/cytosol
C2orf78	A6NCI8	100	1	2	6.0903	Nucleus
CRTAC1	Q9NQ79	71	1	6.8	6.077	Plasma membrane

Given the observed colocalization and interaction between MMP28 and ANXA2, the influence of ANXA2 expression on MMP28-mediated TAM migration and M2 TAM polarization was investigated. Knockdown of ANXA2 in AsPC-1 and BxPC-3 cell lines resulted in a significant reduction in TAM migration compared with that in the control group. Notably, further downregulation of ANXA2 in the context of MMP28 overexpression reversed the stimulatory effect of MMP28 on TAM migration (Fig.S4A). Subsequent analysis of CD86 and CD163 mRNA expression in TAMs cocultured with cancer cells revealed that ANXA2 knockdown increased CD86 expression but suppressed CD163 expression compared with those in the control group. Moreover, the CD163-promoting effect of MMP28 overexpression was attenuated by ANXA2 knockdown, with a concomitant increase in CD86 expression (Fig. S4B). These findings were corroborated by immunofluorescence staining and flow cytometry analyses (Fig. S4C-D), collectively supporting a critical role for ANXA2 in augmenting MMP28-induced TAM recruitment and promoting M2 TAM polarization.

### MMP28 enhances M2 TAM infiltration and promotes pancreatic cancer growth in vivo

**Fig. 9:**
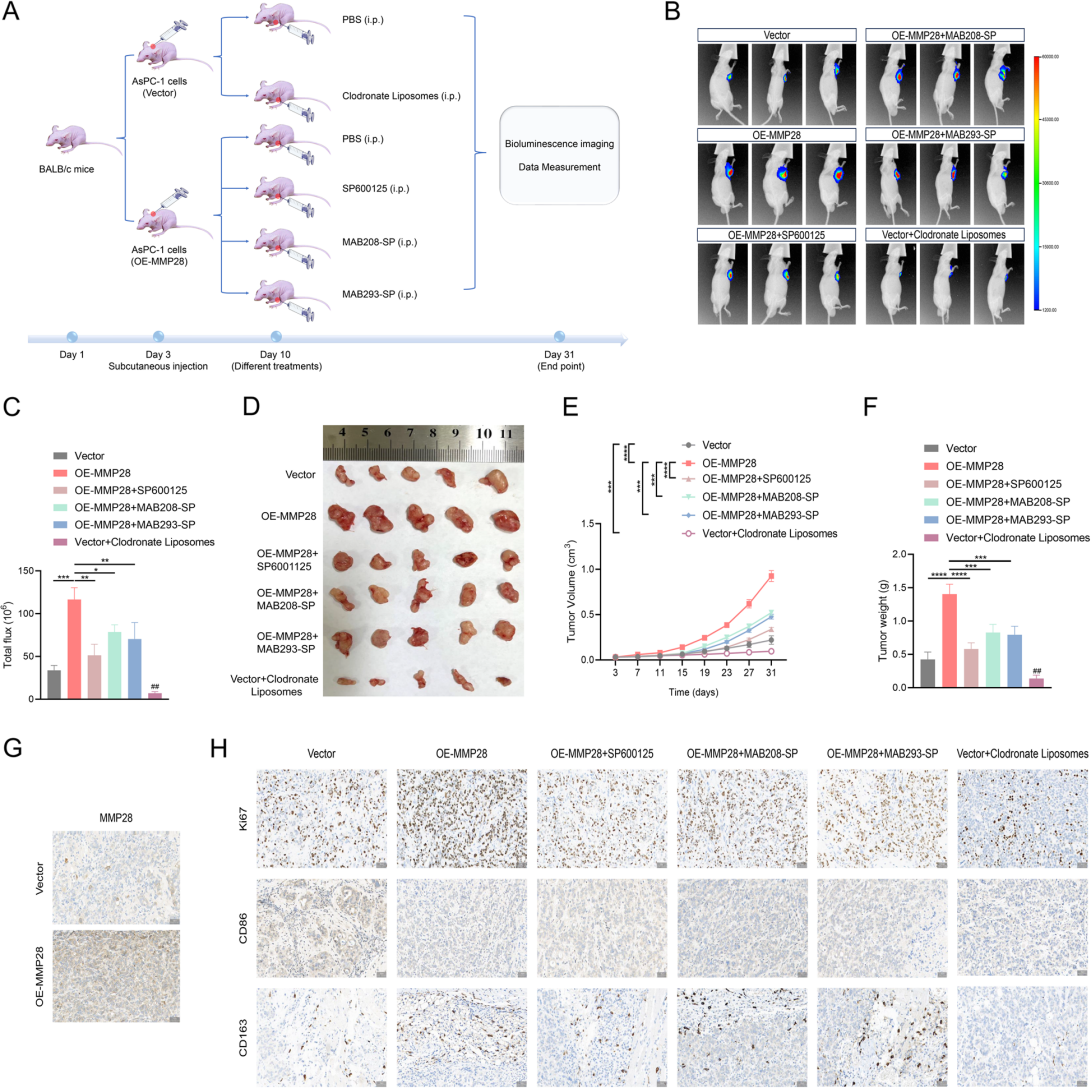
MMP28 enhances M2 TAM infiltration and promotes pancreatic cancer growth in vivo. **A** Effects of the administration of Clodronate Liposomes, the JNK inhibitor SP600125, the anti-IL-8 neutralizing antibody MAB208-SP, and the anti-VEGFA neutralizing antibody MAB293-SP on subcutaneous tumors in nude mice in the experimental group. **B**-**C** Representative images of subcutaneous tumors in nude mice detected with fluorescein potassium salt and the fluorescence quantitative analysis. After the tumor volume reached at least 80mm^3^, PBS, Clodronate Liposomes (200 μg), the JNK inhibitor SP600125 (15 mg/kg), the anti-IL-8 antibody MAB208-SP (10 μg), and the anti-VEGFA antibody MAB293-SP (1 μg) were injected intraperitoneally into the nude mice. **D** Representative images of subcutaneous xenograft tumors (5 mice per group). **E** The volume of the tumor was measured every four days. **F** Tumor weight in both groups. **G** Representative images of MMP28 expression levels in xenograft tumors from the Vector and OE-MMP28 groups as determined by immunohistochemical staining. Scale bar, 50 μm. **H** Immunohistochemical staining detection of expression of Ki67 and CD86 + and CD163 + TAM infiltration in xenograft tumors in the Vector group, OE-MMP28 group, OE-MMP28 + SP600125 group, OE-MMP28 + MAB208-SP group, OE-MMP28 + MAB293-SP group, and Vector + Clodronate Liposomes group

Having established the promigratory and M2-polarizing effects of MMP28 on TAMs in vitro, we sought to investigate the in vivo relevance of these findings. A pancreatic cancer xenograft mouse model was established using AsPC-1 cells, and the animals were subjected to various treatment regimens (Fig. 9A). Compared with the vector control, the overexpression of MMP28 significantly accelerated tumor growth, while the administration of the JNK inhibitor SP600125, the anti-IL-8 antibody MAB208-SP, or the anti-VEGFA antibody MAB293-SP inhibited tumor growth to varying degrees. Additionally, the depletion of macrophages using clodronate liposomes markedly suppressed tumor growth compared with the control (Fig. 9B-F). Immunohistochemical staining analysis confirmed elevated MMP28 expression and increased Ki67-positive cells in the MMP28-overexpressing (OE-MMP28) group relative to those in the vector control group (Fig. 9G-H). Whereas SP600125, MAB208-SP, and MAB293-SP reduced the number of Ki67-positive cells in the OE-MMP28 group, clodronate liposomes significantly decreased the number of Ki67-positive cells in the vector control group. Moreover, both CD86 + and CD163 + TAMs were significantly reduced in the vector control group treated with clodronate liposomes. In the OE-MMP28 group, SP600125, MAB208-SP, and MAB293-SP increased the number of CD86 + TAMs and decreased the number of CD163 + TAM (Fig. 9G-H).

Collectively, the presented data provide compelling evidence that MMP28 enhances pancreatic cancer growth and M2 TAM infiltration in vivo. This protumor effect was partially mitigated by the administration of the JNK inhibitor SP600125, the anti-IL-8 antibody MAB208-SP, the anti-VEGFA antibody MAB293-SP, or the macrophage depletion agent clodronate liposomes.

### MMP28 induces M2 TAM polarization by promoting amino acid metabolism in TAMs

To elucidate the specific mechanism underlying IL-8- and VEGFA-induced M2 TAM polarization, RNA sequencing was performed to identify differentially expressed genes in TAMs cocultured under various experimental conditions. Volcano plot analysis was conducted, and Kyoto Encyclopedia of Genes and Genomes (KEGG) pathway enrichment analysis was subsequently used to identify potential pathways involved in M2 TAM polarization. Compared with the control group, TAMs cocultured with MMP28 overexpressing (OE-MMP28) cancer cells presented 1833 differentially expressed genes (772 upregulated, 1061 downregulated), which are primarily involvedin TAM metabolic alterations, such as amino acid metabolism and glucose metabolism (Fig. S5A-B). The introduction of an anti-IL-8 antibody into TAMs cocultured with OE-MMP28 cancer cells resulted in the expression of 3048 differentially expressed genes, predominantly associated with fatty acid metabolism and protein synthesis (Fig. S5C-D). Similarly, anti-VEGFA antibody treatment led to 1707 differentially expression genes in TAMs (Fig. S5E-F). Comparative analysis of upregulated genes in TAMs cocultured with OE-MMP28 cells with downregulated genes in TAMs treated with anti-IL-8 (MAB208-SP) or anti-VEGFA (MAB293-SP) antibodies revealed significant enrichment in amino acid metabolism-related genes (Fig. S5G-J). We further assessed the overlap of upregulated differentially expressed genes in TAMs co-cultured with OE-MMP28 cells with downregulated genes in TAMs cocultured with OE-MMP28 + MAB208-SP or OE-MMP28 + MAB293-SP cells, respectively, and found that these differentially expressed genes were related mainly to the amino acid metabolism of TAMs (Fig. S5G-J). These findings collectively suggest that IL-8 and VEGFA may primarily promote M2 TAM polarization by modulating TAM amino acid metabolism.

## Discussion

Pancreatic cancer remains a formidable malignancy with limited therapeutic options, resulting in a dismal prognosis for many patients. While immunotherapy has revolutionized the treatment landscape for several solid tumor types, its efficacy in pancreatic cancer has been notably hindered by the unique immunoheterogeneous and immunologically suppressive microenvironment of the tumor. Consequently, immune checkpoint inhibitors have demonstrated limited clinical benefit, with fewer than 15% of patients experiencing positive responses [19]. TAMs, as predominant immune cells within the pancreatic cancer stroma, interact with cancer cells, perpetuating a malignant cycle and exacerbating therapeutic challenges [5]. However, the precise nature of the pancreatic cancer-TAM interaction within the tumor microenvironment remains largely elusive. Furthermore, the consistency with which cancer cells influence TAM behavior across diverse tumor tissues requires further investigation.

Previous studies have shown that TAMs, particularly the polarized M2 phenotype, contribute to pancreatic cancer progression by suppressing antitumor immune responses and facilitating microenvironmental remodeling and angiogenesis [20, 21]. Research has shown that pancreatic cancer cells recruit and reprogram macrophages through the CCL2/CCR2 signaling pathway, leading to the subsequent release of TWEAK via the CCL5/TRAF6/NF-κB pathway. The resulting accumulation of TWEAK activates MURF1-associated muscle atrophy, a key driver of pancreatic cancer cachexia [22]. Ye et al. comprehensively elucidated the crosstalk between macrophages and pancreatic cancer cells and reported that within a macrophage-rich pancreatic cancer microenvironment, cancer cell-derived lactate induces M2 polarization of TAMs, thereby enhancing the Warburg effect in a positive feedback loop. Additionally, M2 TAMs rely on glycolysis to promote cancer cell extravasation, inducing EMT and TGFβ-dependent distant metastasis [23–25]. Moreover, macrophages interact with other microenvironmental components to exacerbate pancreatic cancer malignancy. Macrophages regulate fibroblast activation and extracellular matrix deposition through granule protein secretion, influencing vascular and lymphatic angiogenesis and immunosuppression, whereas fibroblast sialylation induces monocyte differentiation into immunosuppressive TAMs, further promoting microenvironmental malignancy [26, 27]. Disrupting the tumor's ecological niche and enhancing pancreatic cancer treatment efficacy may be achieved by inhibiting TAM recruitment or reversing M2 TAM polarization.

MMPs significantly influence tumor growth, angiogenesis, and metastasis by regulating the release or activation of chemokines, cytokines, and growth factors [28]. Furthermore, MMPs are closely associated with the inflammatory microenvironment, particularly in controlling leukocyte trafficking and activation [29]. However, the interplay between the tumor microenvironment and MMPs remains largely unexplored. Previous single-cell RNA sequencing studies revealed that MMP28 is expressed predominantly within pancreatic ductal epithelial cells, highlighting the specific expression of MMP28 in pancreatic cancer [30]. The present study demonstrated that tumor-derived MMP28 promotes TAM recruitment and induces M2 TAM polarization, thereby enhancing pancreatic cancer growth and proliferation in vitro and in vivo. Consequently, our findings establish that MMP28, which is overexpressed in pancreatic cancer, is a key driver of tumor progression through its interaction with TAMs within the microenvironment, revealing a novel function for this protein. Given its relatively recent discovery within the MMP family, the regulatory significance of MMP28 in tumor progression is particularly noteworthy. Considering the diverse nature of tumors and the heterogeneous characteristics of the tumor microenvironment, further investigation is warranted to determine whether other MMPs exhibit similar functionalities.

Our previous investigations confirmed the capacity of MMP28 to induce TAM chemotaxis and M2 TAM polarization through immunohistochemical staining analyses and complementary in vivo and in vitro cell experiments. To elucidate the underlying mechanism by which MMP28 mediates TAM chemotaxis and M2 TAM polarization within cancer cells, we explored potential intermediary factors. Considering that TAM recruitment and polarization are influenced by exogenous factors, including tumor cell-secreted cytokines and chemokines, and given the established role of MMPs in regulating microenvironmental function, it is highly conceivable that MMP28 could modulate TAM behavior by influencing cytokine production and secretion. Cytokine antibody array analysis revealed that MMP28 knockdown suppressed the production and release of IL-2, IL-5, IL-8, MCP-1, and VEGFA by cancer cells. By correlating the cytokine secretion data with the qRT-PCR findings, we determined that MMP28 primarily impacts IL-8 and VEGFA production and secretion. Moreover, the inhibition of these cytokines effectively blocked the effect of MMP28 on TAMs. Consequently, targeting IL-8 and VEGFA production and secretion has emerged as a promising strategy for TAM-based pancreatic cancer treatment.

The MAPK signaling pathway constitutes a critical component of the eukaryotic signaling network, exerting a significant influence on cell proliferation, metabolic adaptation, survival, and differentiation [31]. Previous studies have implicated the MAPK signaling pathway in mediating the production and activation of cytokines, including IL-6 and IL-8, which subsequently impact tumor cell survival and immune cell recruitment [32, 33]. In the present study, we confirmed through Western blotting that MMP28 regulates MAPK signal transduction in AsPC-1 and BxPC-3 cells, with JNK phosphorylation levels mirroring those of MMP28, which corroborated previous findings. Inhibition of the MAPK/JNK signaling pathway resulted in suppressed TAM recruitment, M2 TAM polarization, and IL-8 and VEGFA production and secretion by cancer cells. These findings suggest that the MAPK/JNK pathway serves as a pivotal molecular signal through which MMP28 regulates IL-8 and VEGFA secretion in cancer cells. In vivo experiments were used to evaluate the effects of a JNK inhibitor, an IL-8 neutralizing antibody, and a VEGFA neutralizing antibody on tumor proliferation and the tumor microenvironment. The impact of MMP28 on pancreatic cancer growth and TAMs was partially reversed by inhibiting either the JNK signaling pathway or the effects of IL-8 and VEGFA. Furthermore, macrophage depletion in mice also suppressed pancreatic cancer proliferation, indicating that targeting cancer cell-TAM communication is a promising strategy for preventing pancreatic cancer progression.

As a calcium-dependent phospholipid plasma membrane-binding protein, ANXA2 is overexpressed in various tumor tissues, including lung cancer and cholangiocarcinoma tissues, and is closely associated with tumor cell proliferation, apoptosis, invasion, and metastasis [34, 35]. Within the breast cancer microenvironment, exosomes containing ANXA2 stimulate the activation of MAPK and NF-κB signaling pathways in macrophages, thereby mediating the secretion of the chemokines IL-6 and TNF-α to remodel the microenvironment to support breast cancer cell survival [36]. Immunoprecipitation and mass spectrometry confirmed a physical interaction between MMP28 and ANXA2. Moreover, inhibiting ANXA2 expression partially reversed the effects of MMP28 on TAMs, suggesting that the MMP28-ANXA2 interaction influences TAM infiltration and polarization, thereby representing a potential therapeutic target for TAM-based pancreatic cancer treatments.

The precise mechanisms underlying M2 TAM polarization within the pancreatic cancer microenvironment remain to be elucidated in the present study. To further investigate the impact of IL-8 and VEGFA on M2 TAM polarization, RNA sequencing was performed. The results indicated significant metabolic alterations in TAMs following the administration of IL-8- and VEGFA-neutralizing antibodies, particularly in amino acid metabolism, suggesting a potential role in M2 TAM polarization. These findings warrant further exploration of the underlying mechanism of these changes in subsequent studies.

## Conclusion

In summary, our study elucidated the specific mechanism by which pancreatic cancer cells recruit TAMs and induce their polarization towards the M2 phenotype. Briefly, MMP28, which is overexpressed in pancreatic cancer, mediates the recruitment of TAMs through phosphorylation of the MAPK/JNK signaling pathway, stimulating the secretion of IL-8 and VEGFA by cancer cells. IL-8 and VEGFA subsequently enter the TAM cytosol, where they bind to their respective receptors to influence amino acid metabolism and ultimately promote M2 TAM polarization. Furthermore, ANXA2 amplifies MMP28-mediated M2 TAM infiltration, collectively contributing to the malignant progression of pancreatic cancer.

## Supplementary Information

Supplementary Material 1. Figure S1. Effect of MMP28 overexpression on the proliferation, migration and invasion of pancreatic cancer cells. (A-B) Empty lentivirus (Vector) and MMP28-overexpressing lentivirus (OE-MMP28) were transfected into AsPC-1 and BxPC-3 cells, and the overexpression efficiency of MMP28 was verified by qRT-PCR and WB. (C) An EdU kit was used to assess the proliferation ability of the indicated cancer cells. Scale bar, 50 μm. (D) CCK8 reagent was used to assess the proliferation ability of the indicated cancer cells. (E) Migration and invasion ability of the indicated cells were analysed by a Transwell assay. Scale bar, 100 μm. (F) The levels of apoptosis in the two cancer cells were analysed by flow cytometry. **P<0.01,*** P <0.001, and ****P<0.0001.

Supplementary Material 2. Figure S2. Effect of MMP28 on apoptosis and EMT in pancreatic cancer cells. (A) Empty lentivirus (sh-NC) and MMP28 knockdown lentivirus (sh-MMP28) were transfected into AsPC-1 and BxPC-3 cells. The effects of MMP28 knockdown on the expression of apoptosis-related proteins (BAX and BCL2) and EMT-related proteins (CDH1, CDH2 and Vimentin) in pancreatic cancer were detected by WB. (B) Empty lentivirus (Vector) and MMP28-overexpressing lentivirus (OE-MMP28) were transfected in AsPC-1 and BxPC-3 cells, and the effects of MMP28 overexpression on the expression of apoptosis-related proteins (BAX and BCL2) and EMT-related proteins (CDH1, CDH2, and Vimentin) in pancreatic cancer cells were determined by WB. *P<0.05, **P<0.01, ***P<0.001, and ****P<0.0001.

Supplementary Material 3. Figure S3. MMP28 overexpression promotes the migration of TAMs and polarization of the M2 TAMs. (A) The ability of CM from cancer cells in the Vector group and the OE-MMP28 group to attract TAMs was assessed in 8 μm Transwell chambers. Compared with CM from the OE-MMP28 group, the effects of the addition of the JNK inhibitor SP600125 on the ability of cancer cells to promote TAM migration were determined. Scale bar, 100 μm. (B) qRT-PCR was used to determine the expression levels of CD86 and CD163 TAM markers, in different coculture groups. (C) The expression levels of the TAM markers CD86 and CD163 in different coculture groups were determined by immunofluorescence staining. Scale bar, 50 μm. (D) The proportions of CD11b+CD86+ TAMs and CD11b+CD163+ TAMs in different coculture groups were analysed by flow cytometry. *P<0.05, **P<0.01,***P<0.001, and ****P<0.0001. #*P*<0.05, ##*P*<0.01, and ####*P*<0.0001.

Supplementary Material 4. Figure S4. ANXA2 enhances the ability of MMP28 to promote the migration of TAMs and the polarization of M2 TAMs. (A) The ability of CM from cancer cells to attract TAMs in the Vector+Control group, siANXA2 group, OE-MMP28+Control group and OE-MMP28+siANXA2 group was determined with 8 μm Transwell chambers. Plasmids containing small interfering RNAs specific for ANXA2 and empty plasmids were added to Vector group and OE-MMP28 group, respectively. Scale bar, 100 μm. (B) The expression levels of the TAM markers CD86 and CD163 in cancer cells cocultured with the Vector+Control group, siANXA2 group, OE-MMP28+Control group, and OE-MMP28+siANXA2 group were determined by qRT-PCR. (C) The expression levels of TAMs markers CD86 and CD163 in different coculture groups were determined by immunofluorescence staining. Scale bar, 50 μm. (D) The proportions of CD11b+CD86+ TAMs and CD11b+CD163+ TAM in the above co-culture group were analyzed by flow cytometry. *P<0.05, **P<0.01, ***P<0.001, and ****P<0.0001. and #*P*< 0.05, ##*P*<0.01, ###*P*<0.001.

Supplementary Material 5. Figure S5. MMP28 induces M2 TAM polarization by promoting amino acid metabolism in TAMs. (A-B) RNA sequencing was performed on TAMs cocultured with control (Vector) and MMP28-overexpressing (OE-MMP28) AsPC-1 cells. Volcano mapping and KEGG pathway enrichment analysis were subsequently performed for differentially expressed genes in TAMs. (C-D) RNA sequencing was performed on TAMs cocultured with AsPC-1 cells of MMP28-overexpressing group (OE-MMP28) and MMP28-overexpressing+MAB208-SP group (OE-MMP28+MAB208-SP), and the differentially expressed genes in TAMs were mapped by volcano plot and analysed by KEGG pathway enrichment. (E-F) RNA sequencing was performed on TAMs cocultured with AsPC-1 cells of the MMP28-overexpressing group (OE-MMP28) and the MMP28-overexpressing+MAB293-SP group (OE-MMP28+MAB293-SP), and the differentially expressed genes were mapped by a volcano plot and analysed by KEGG pathway enrichment. (G-H) The overlap of upregulated differentially expressed in TAMs cocultured with OE-MMP28 cells with downregulated differentially expressed genes in TAMs cocultured with OE-MMP28+MAB208-SP cells identified by RNA sequencing was determined and analysed for KEGG pathway enrichment. (I-J) The overlap of upregulated differentially expressed genes in TAMs cocultured with OE-MMP28 cells with downregulated differentially expressed genes in TAMs cocultured with OE-MMP28+MAB293-SP cells identified by RNA sequencing were taken and analyzed for KEGG pathway enrichment.

Supplementary Material 6.

Supplementary Material 7.

## Data Availability

The datasets related to this article have been uploaded to the GEO database (GSE283101, GSE283259).
