# Supplementary figures and images for: MMP28 recruits M2-type tumor-associated macrophages through MAPK/JNK signaling pathway-dependent cytokine secretion to promote the malignant progression of pancreatic cancer
Source: J Exp Clin Cancer Res. 2025 Feb 19;44:60. doi: 10.1186/s13046-025-03321-x (PMC11837641; doi:10.1186/s13046-025-03321-x)

A

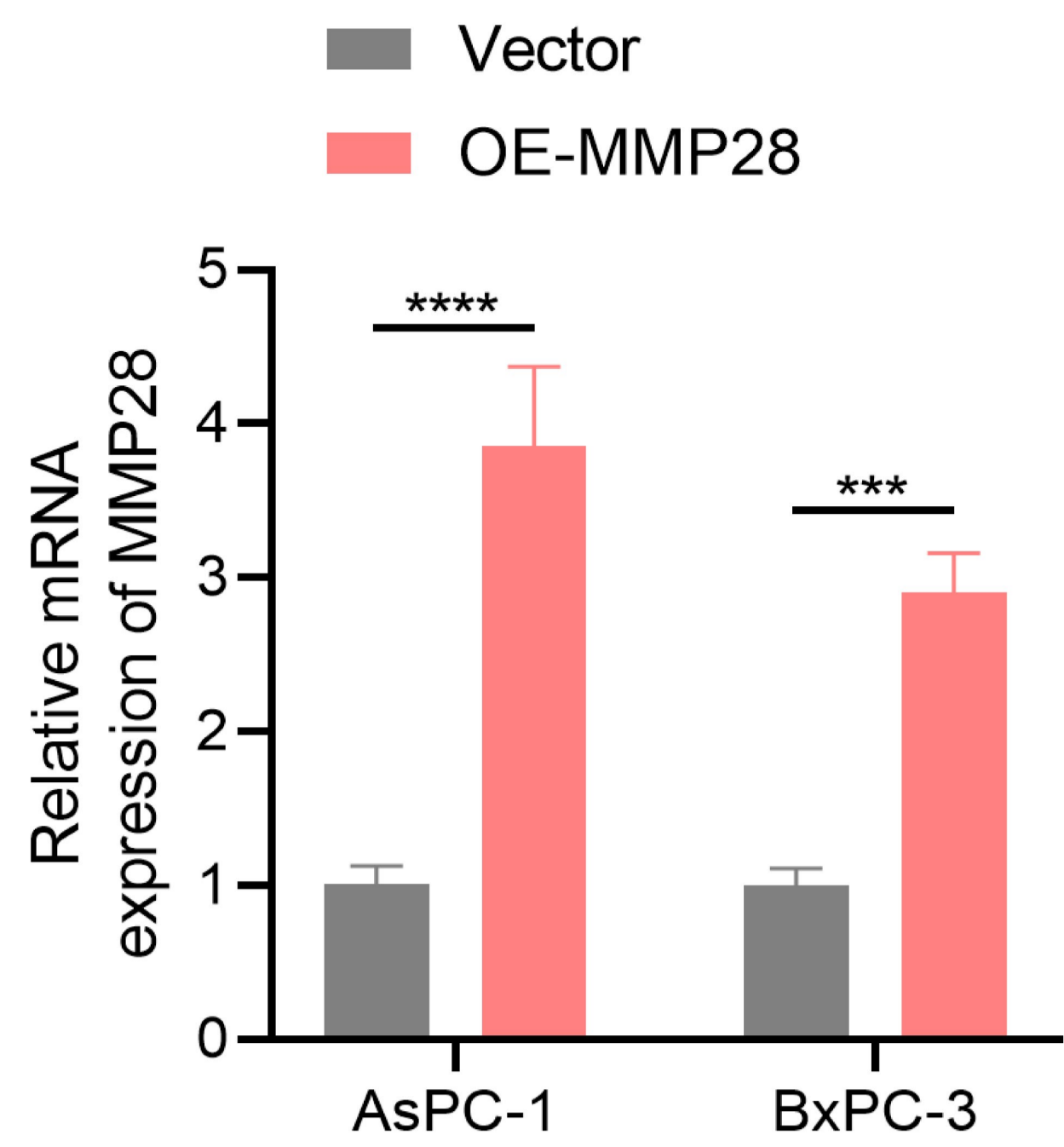

B

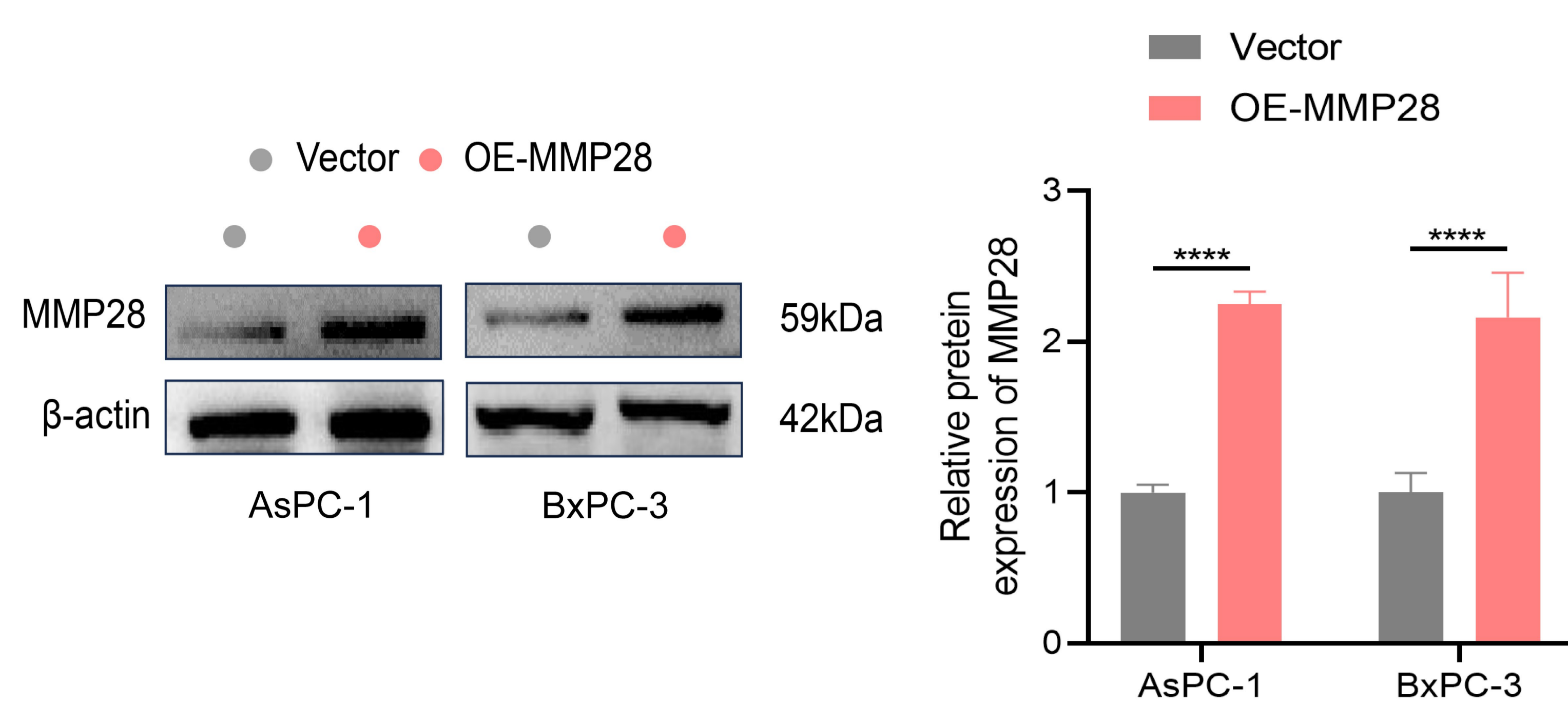

C

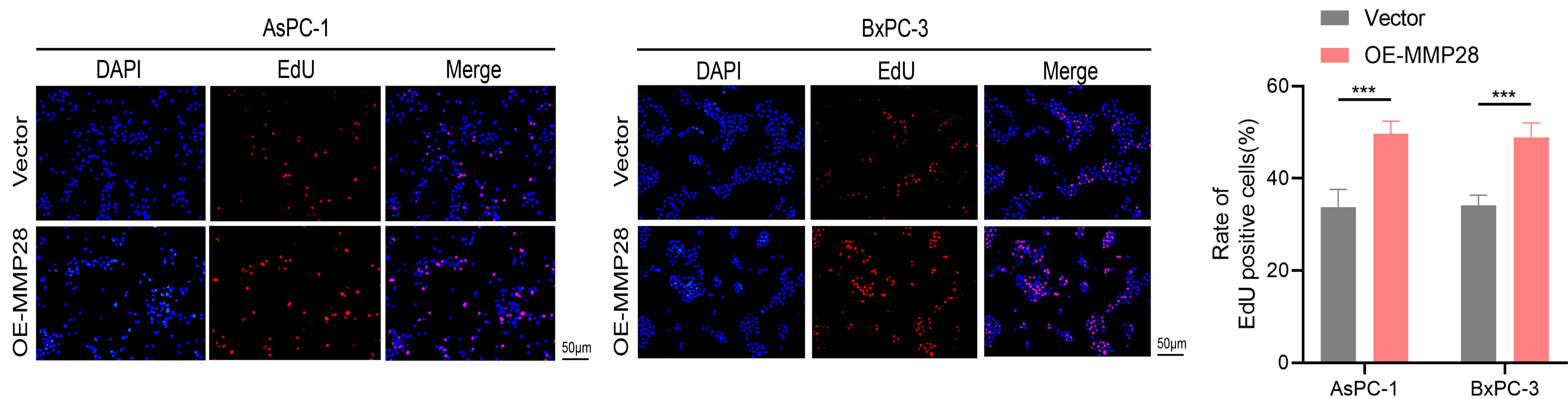

D

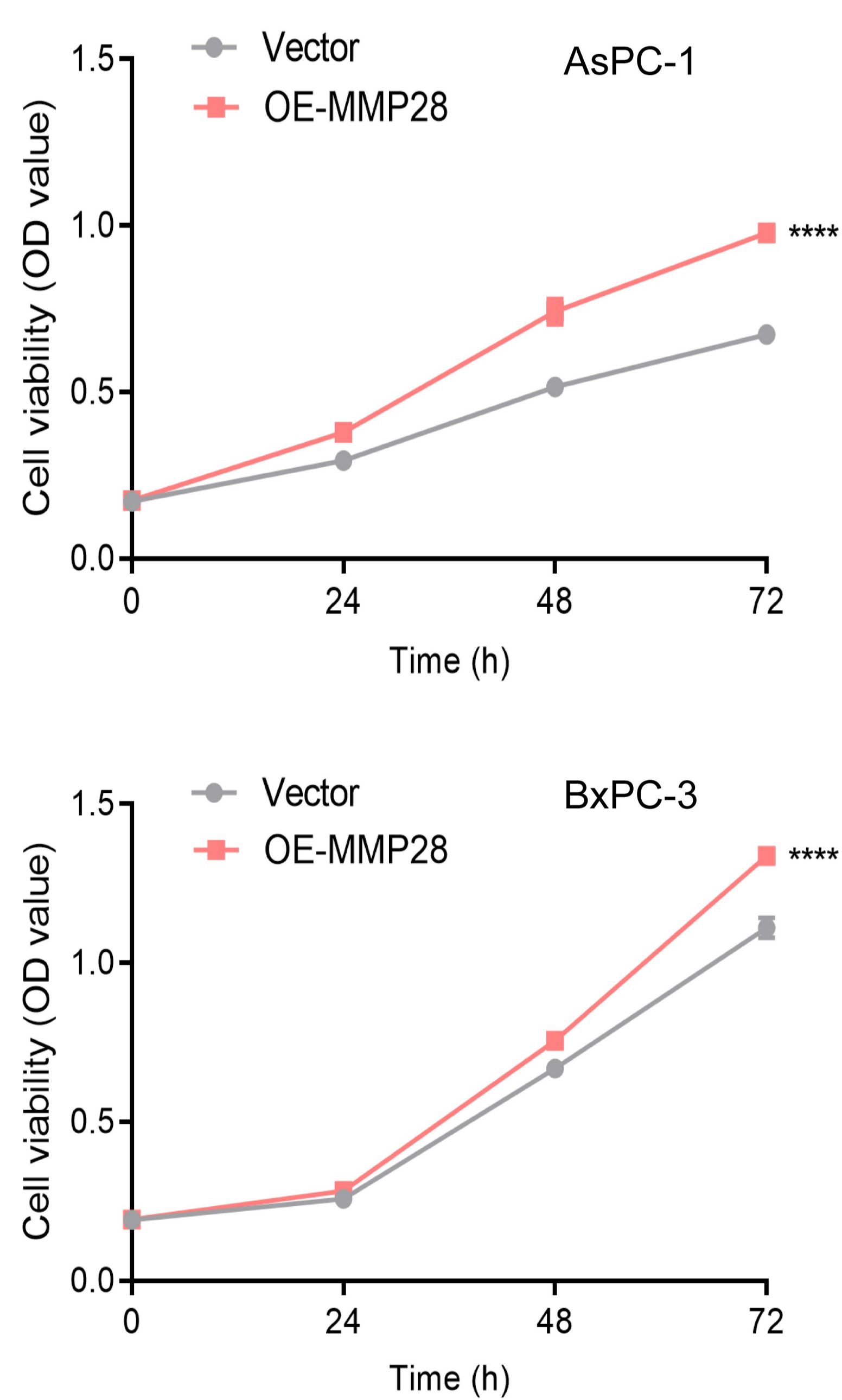

E

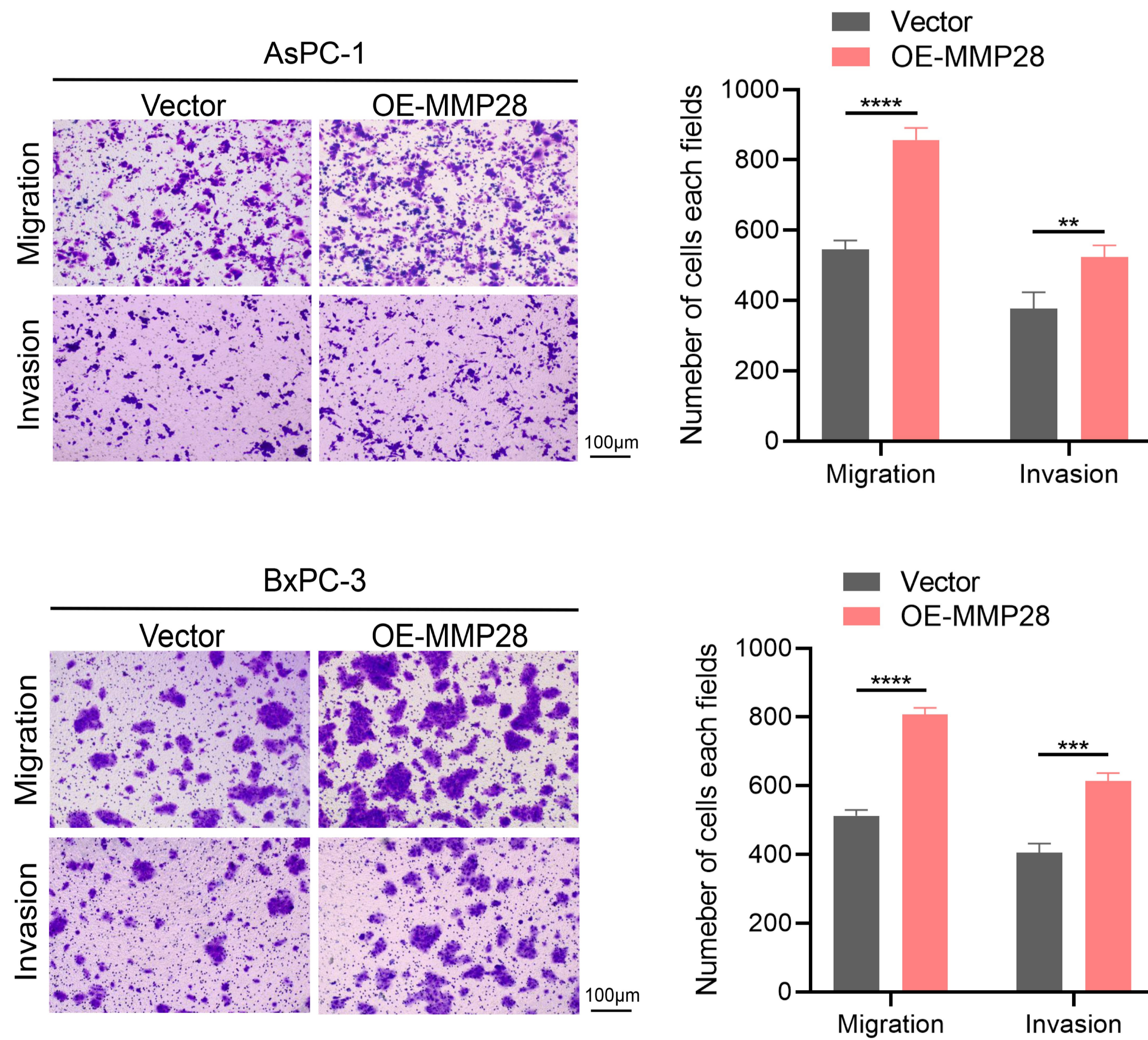

F

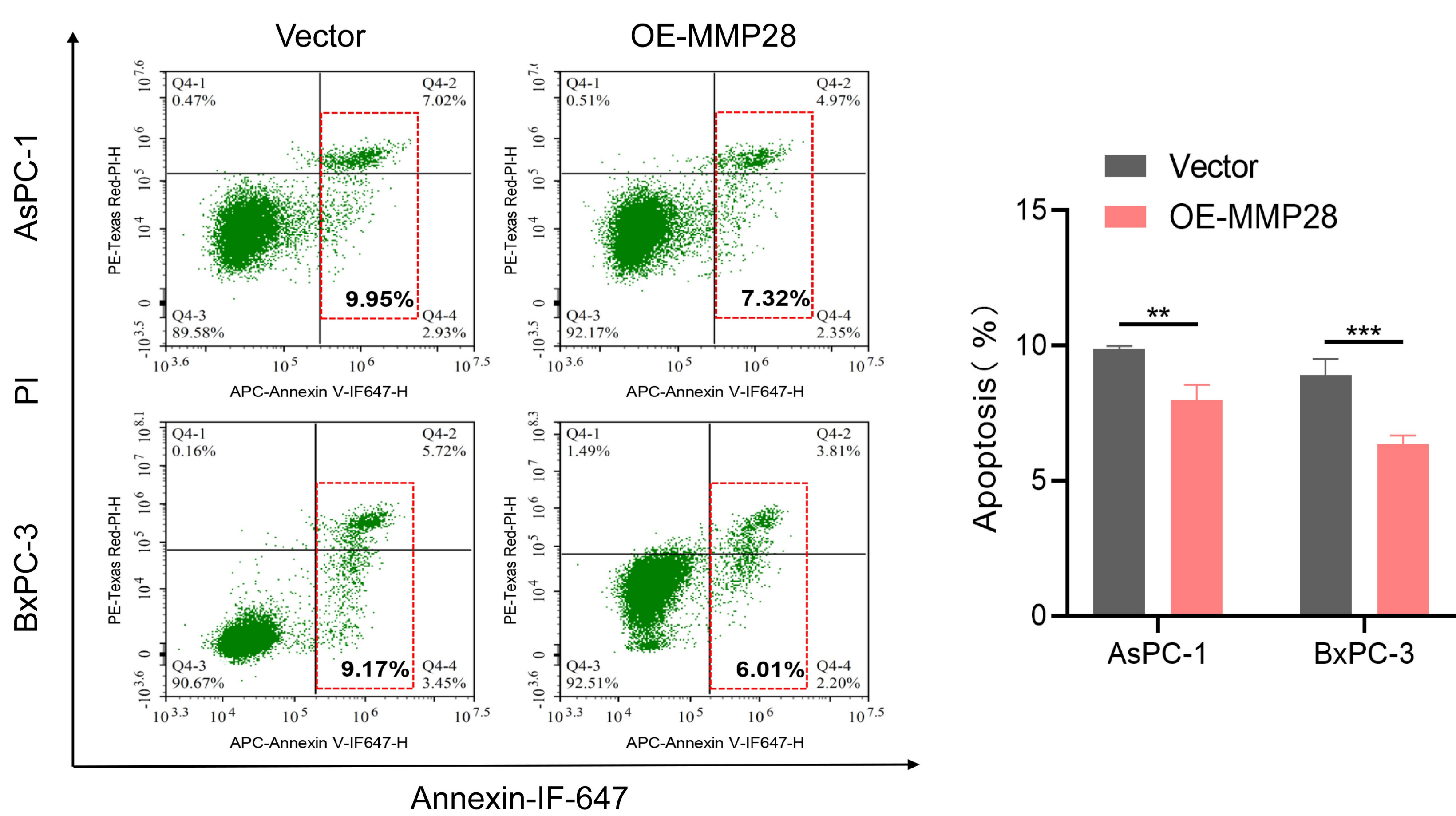

Supplement: Supplementary file 1 — Supplementary Material 1. Figure S1. Effect of MMP28 overexpression on the proliferation, migration and invasion of pancreatic cancer cells. (A-B) Empty lentivirus (Vector) and MMP28-overexpressing lentivirus (OE-MMP28) were transfected into AsPC-1 and BxPC-3 cells, and the overexpression efficiency of MMP28 was verified by qRT-PCR and WB. (C) An EdU kit was used to assess the proliferation ability of the indicated cancer cells. Scale bar, 50 μm. (D) CCK8 reagent was used to assess the proliferation ability of the indicated cancer cells. (E) Migration and invasion ability of the indicated cells were analysed by a Transwell assay. Scale bar, 100 μm. (F) The levels of apoptosis in the two cancer cells were analysed by flow cytometry. **P<0.01,*** P <0.001, and ****P<0.0001. [file 13046_2025_3321_MOESM1_ESM.pdf]

A

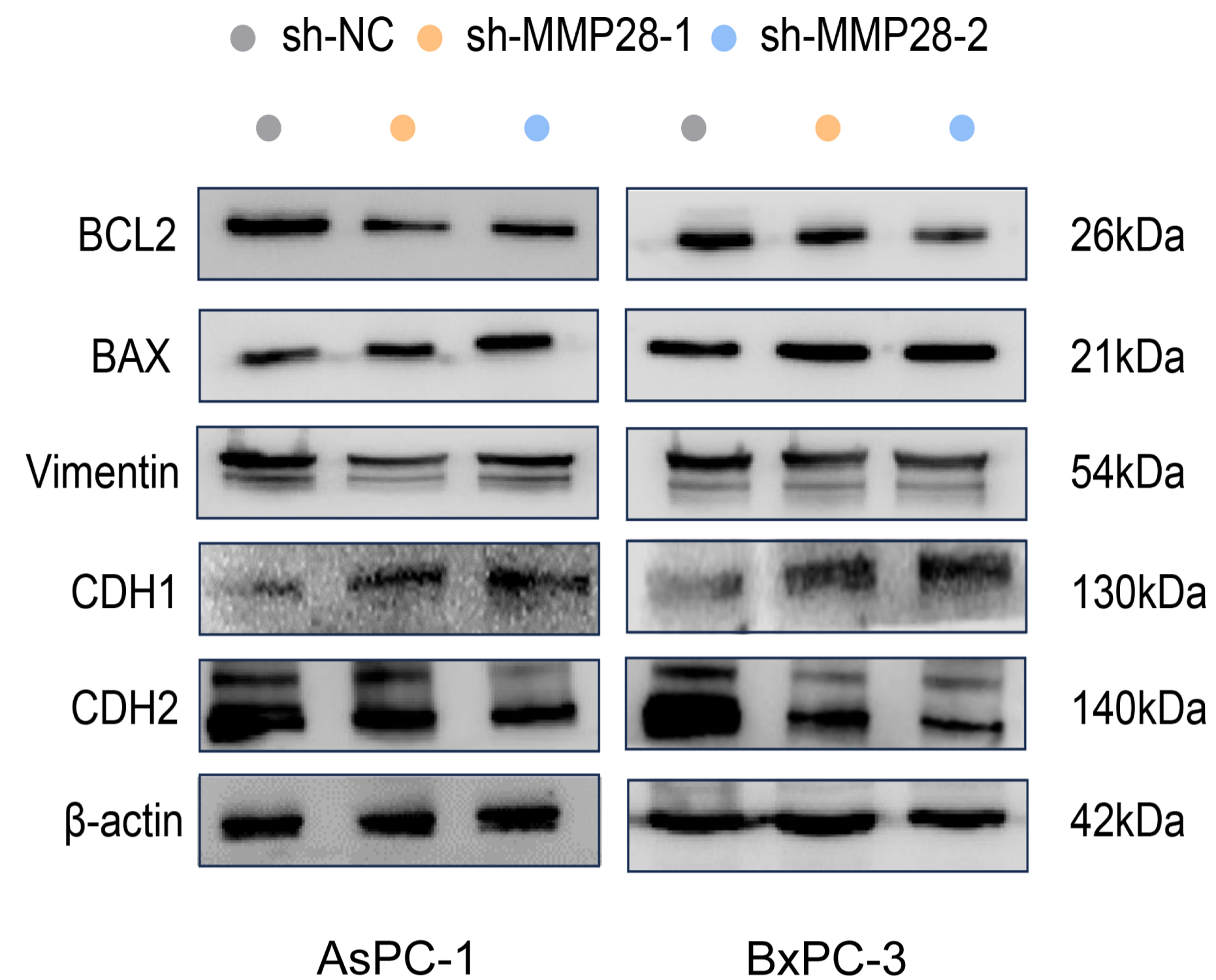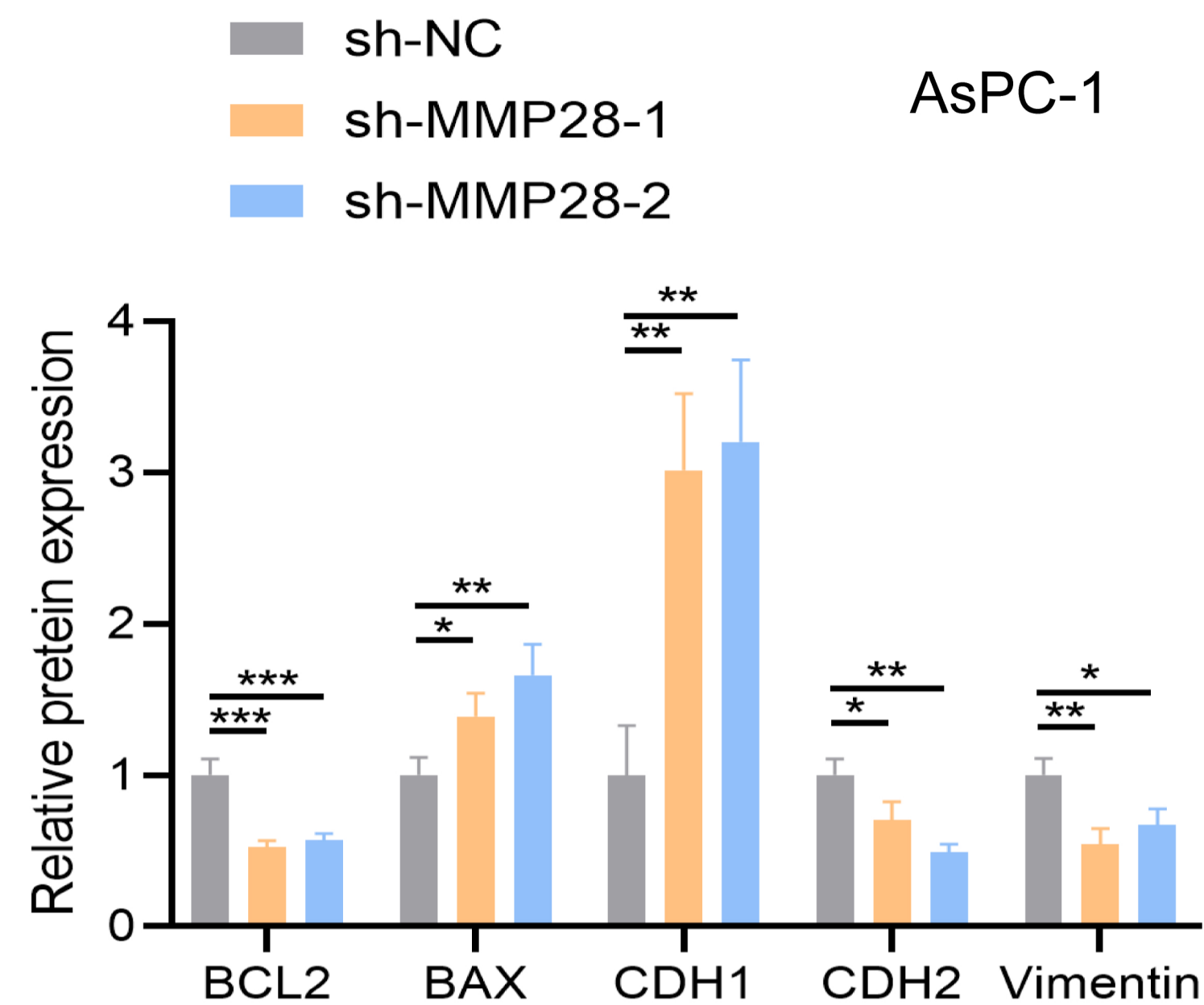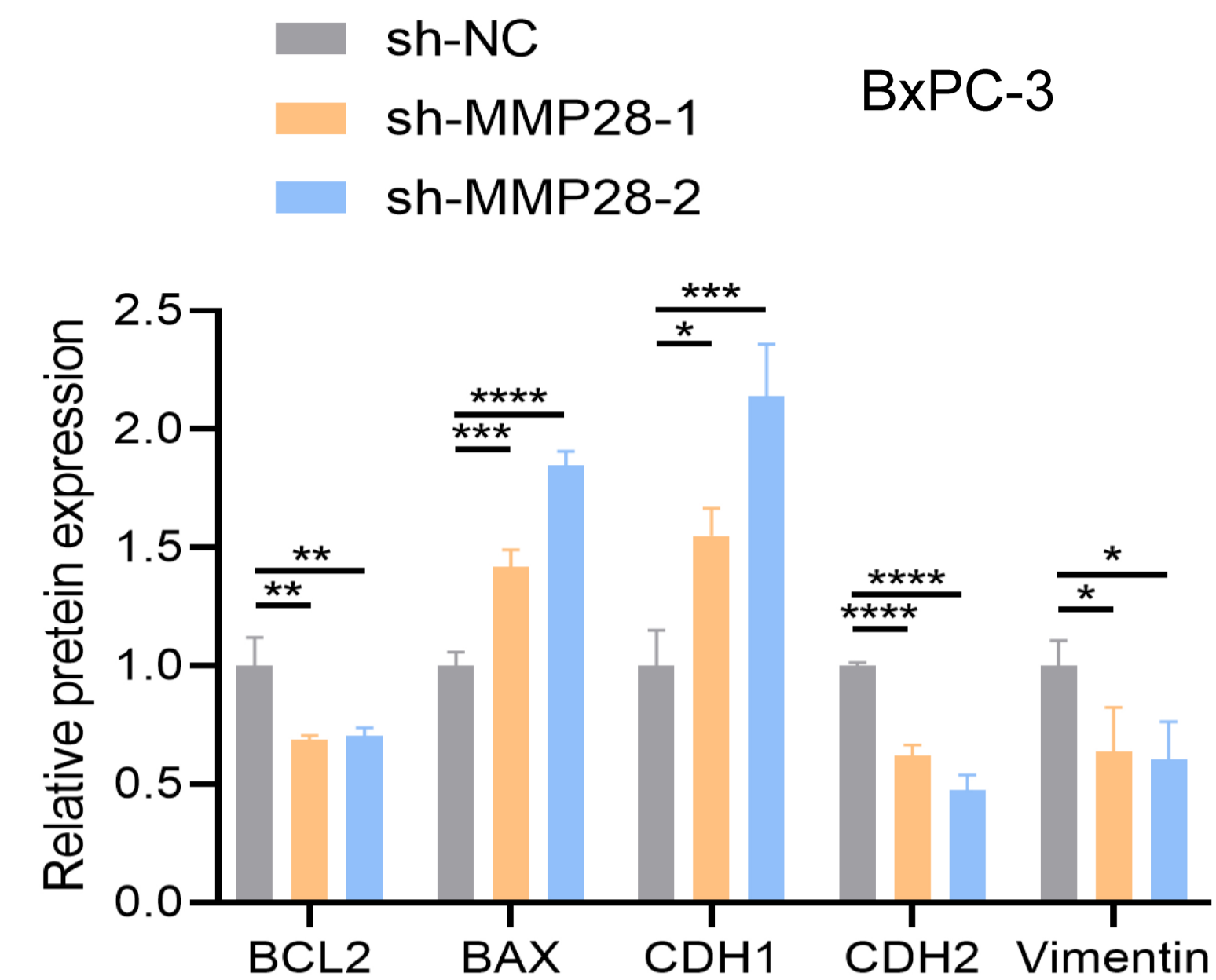

B

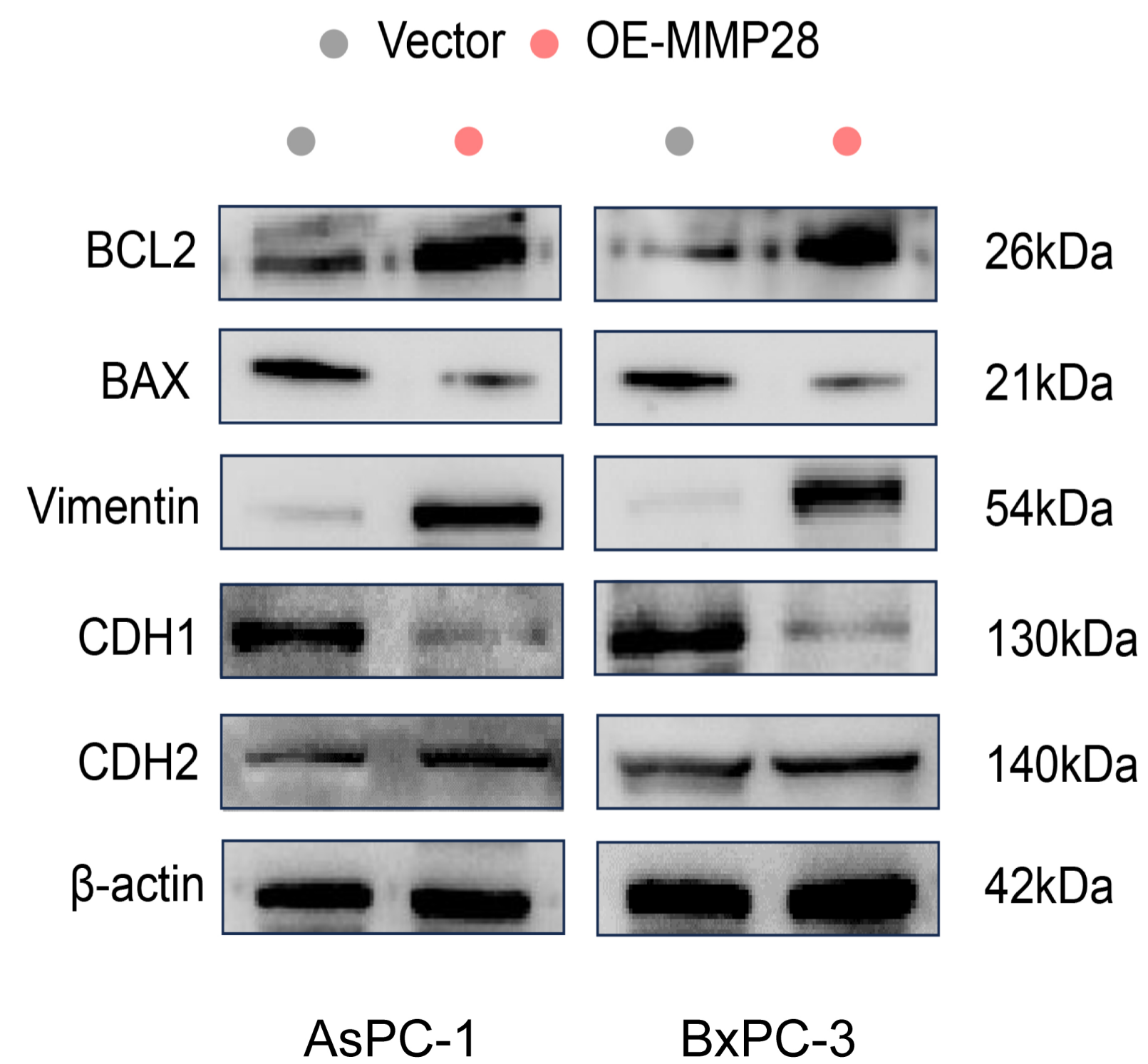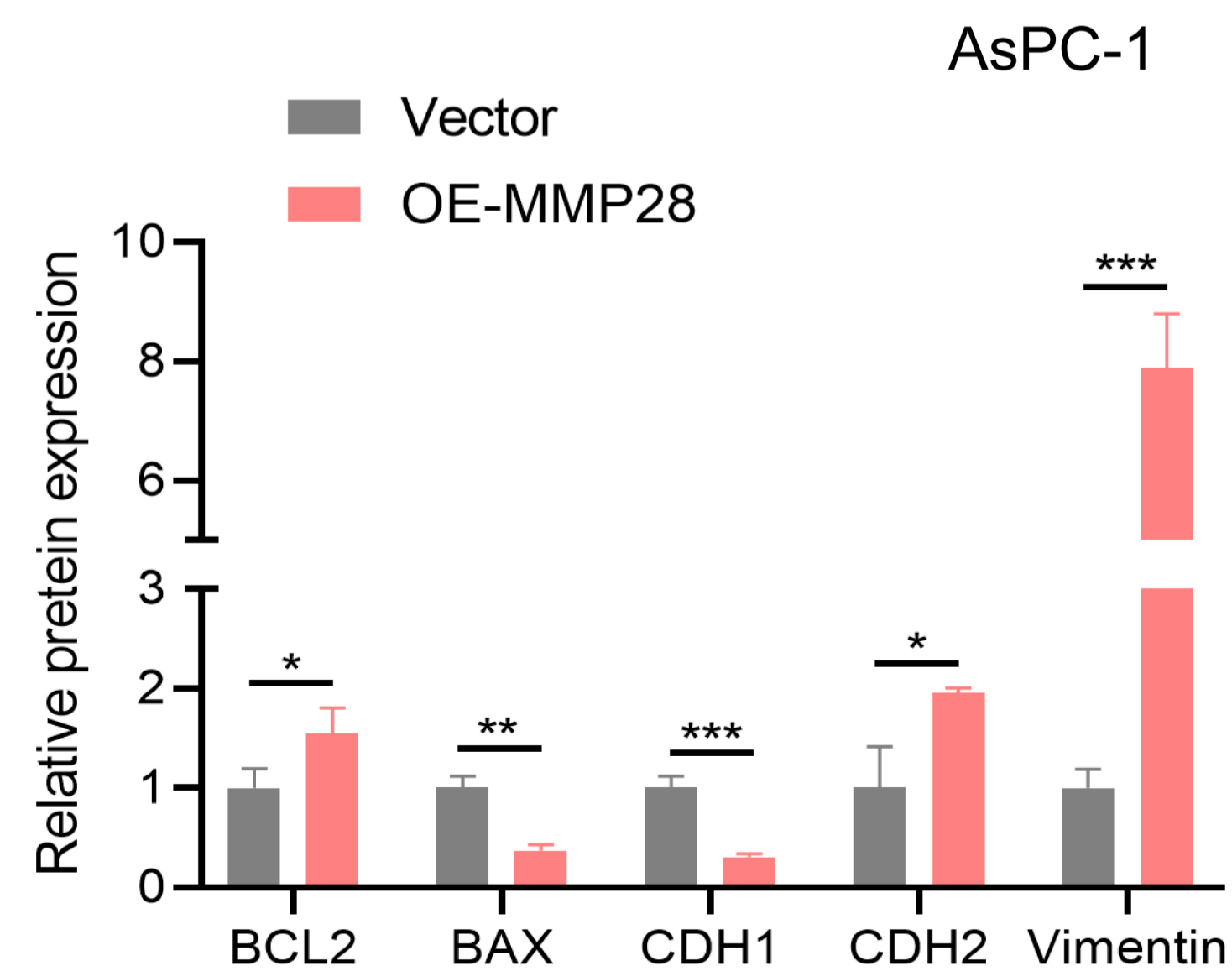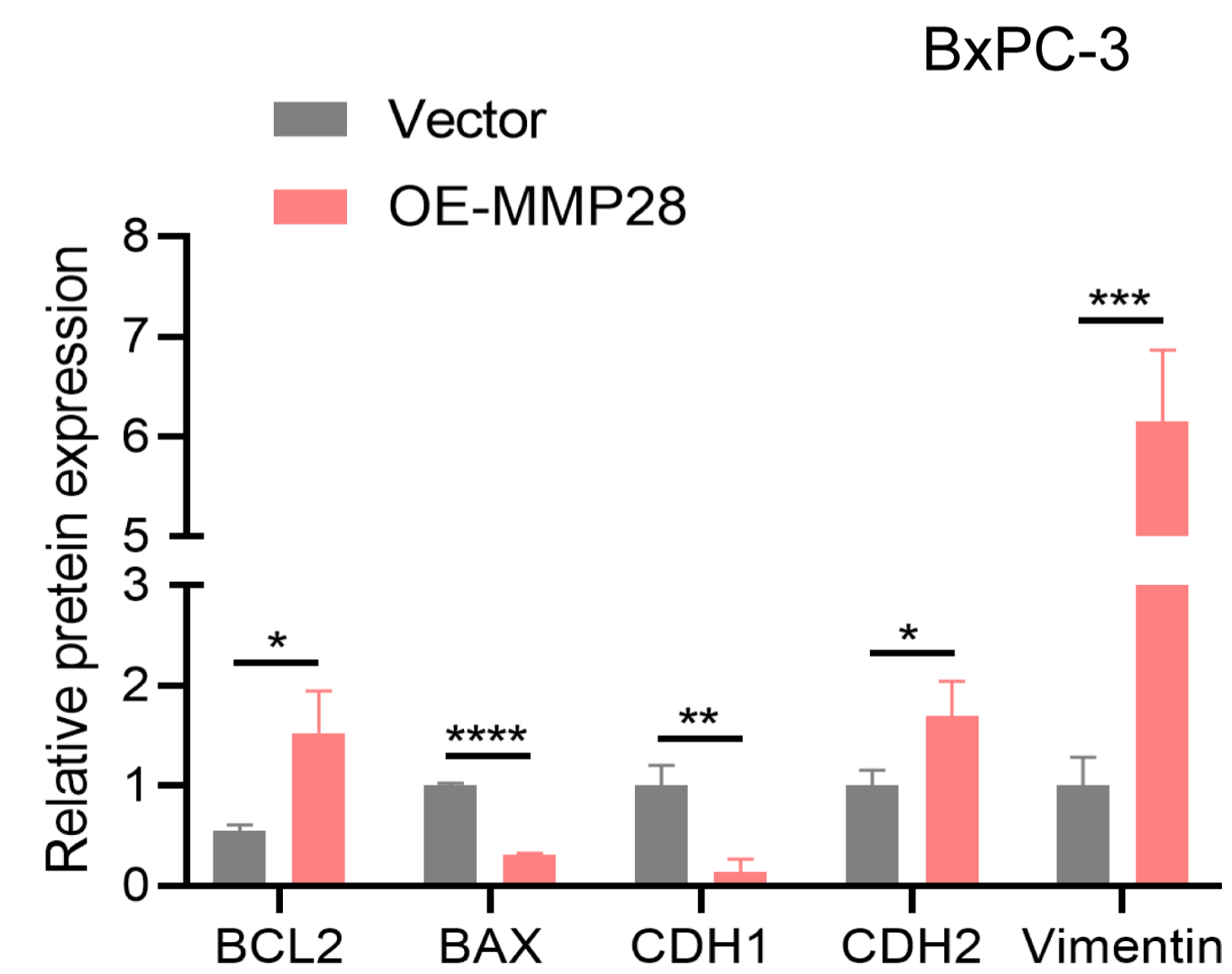

Supplement: Supplementary file 2 — Supplementary Material 2. Figure S2. Effect of MMP28 on apoptosis and EMT in pancreatic cancer cells. (A) Empty lentivirus (sh-NC) and MMP28 knockdown lentivirus (sh-MMP28) were transfected into AsPC-1 and BxPC-3 cells. The effects of MMP28 knockdown on the expression of apoptosis-related proteins (BAX and BCL2) and EMT-related proteins (CDH1, CDH2 and Vimentin) in pancreatic cancer were detected by WB. (B) Empty lentivirus (Vector) and MMP28-overexpressing lentivirus (OE-MMP28) were transfected in AsPC-1 and BxPC-3 cells, and the effects of MMP28 overexpression on the expression of apoptosis-related proteins (BAX and BCL2) and EMT-related proteins (CDH1, CDH2, and Vimentin) in pancreatic cancer cells were determined by WB. *P<0.05, **P<0.01, ***P<0.001, and ****P<0.0001. [file 13046_2025_3321_MOESM2_ESM.pdf]

A

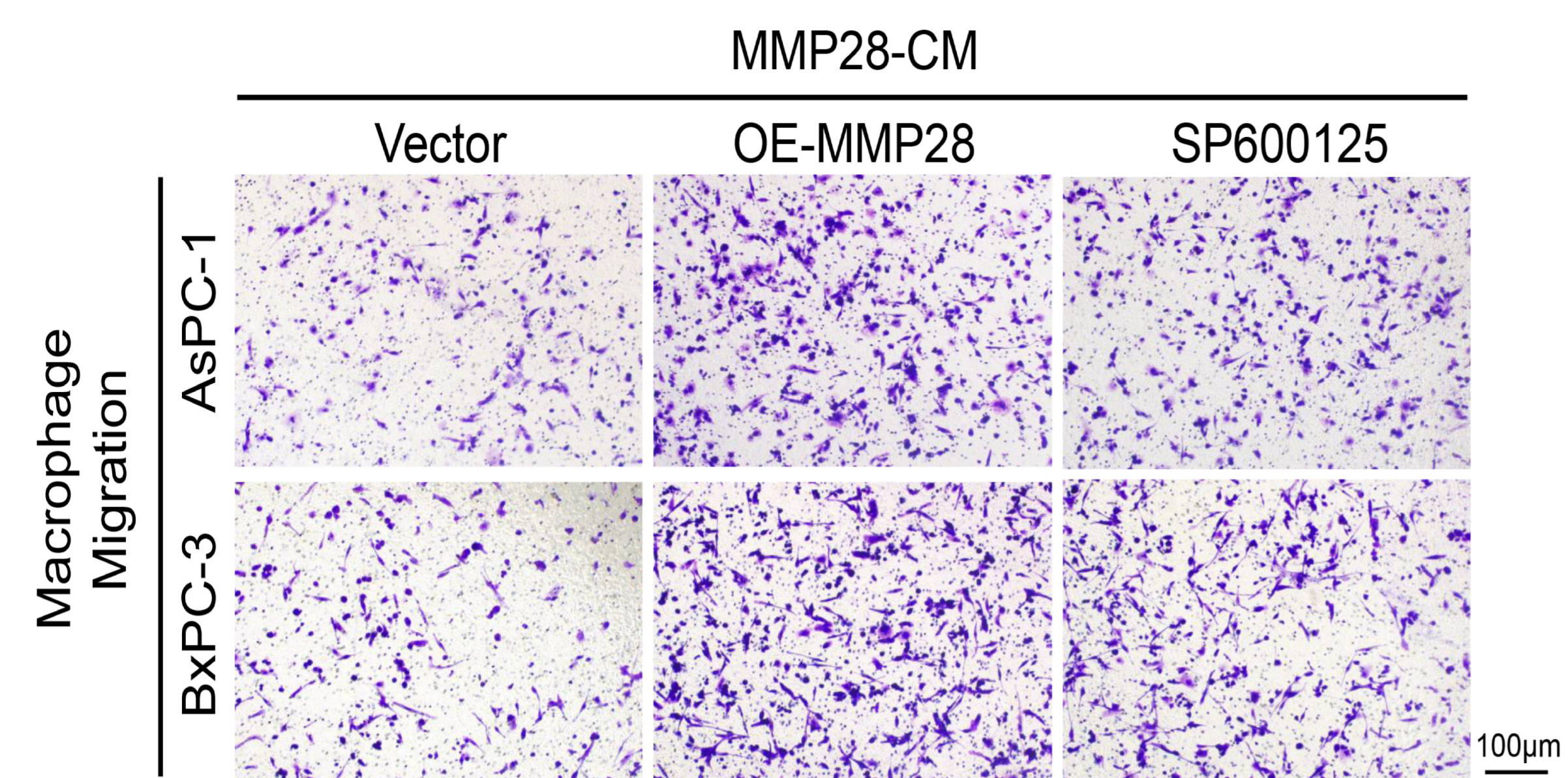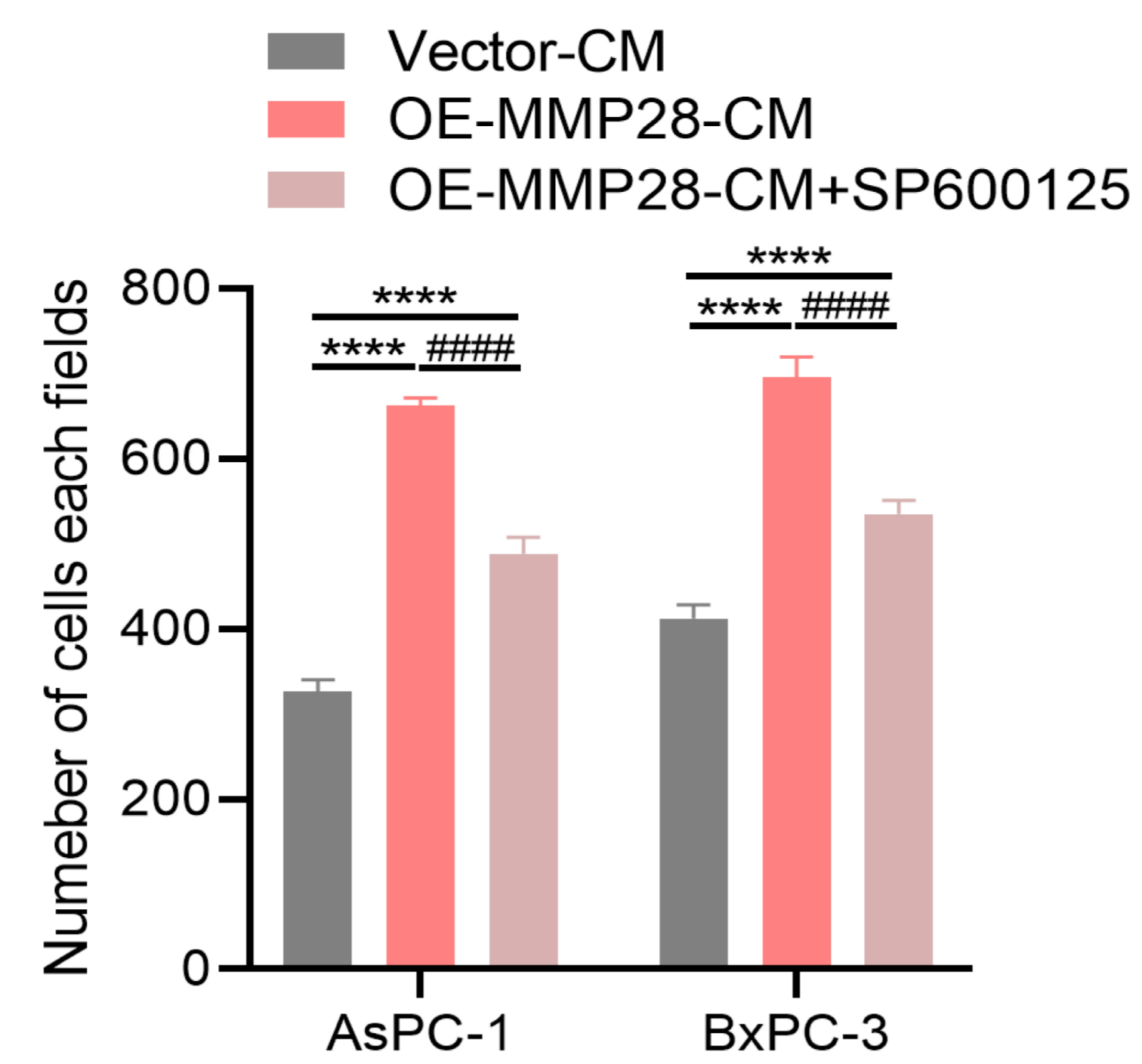

B

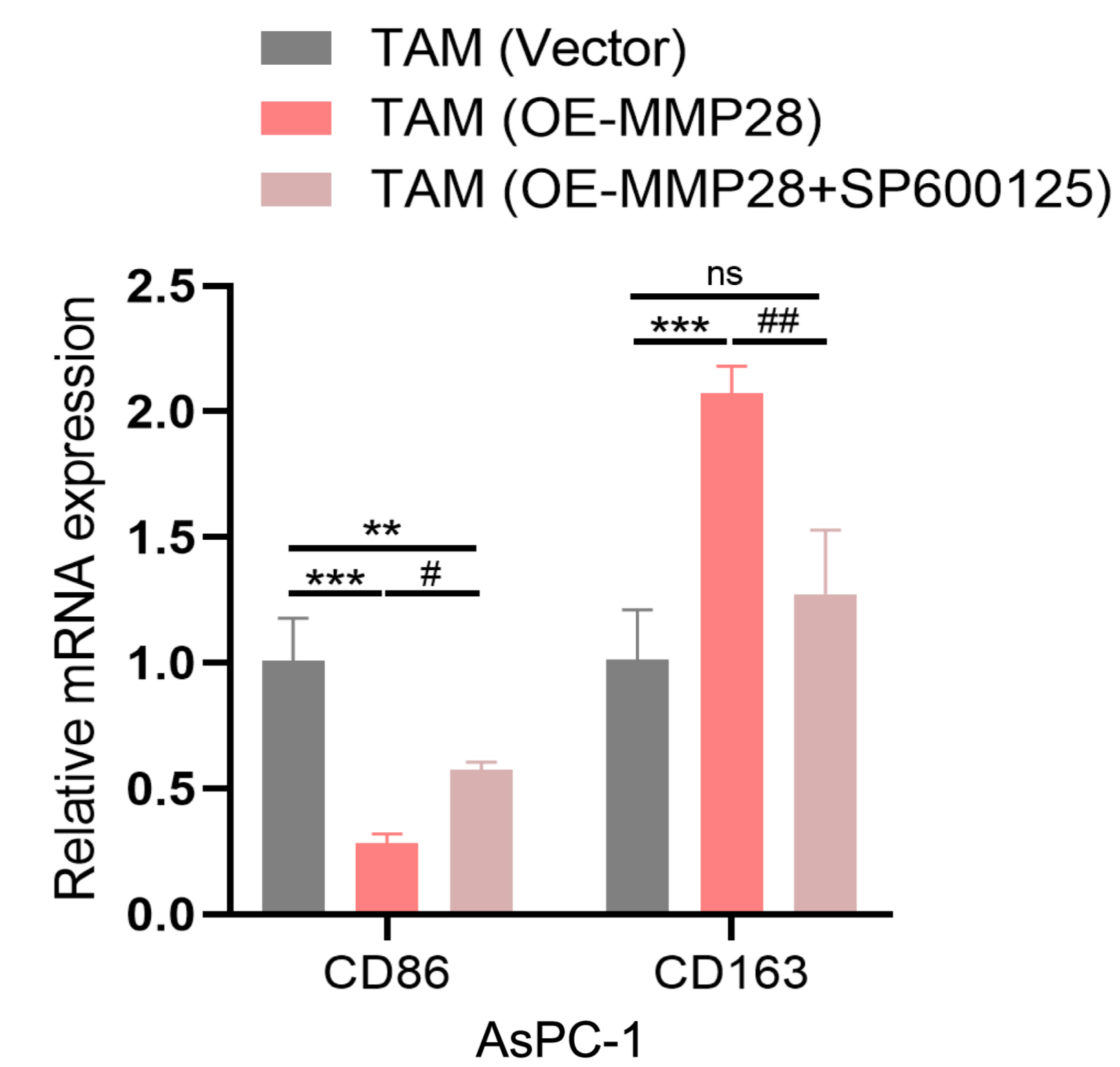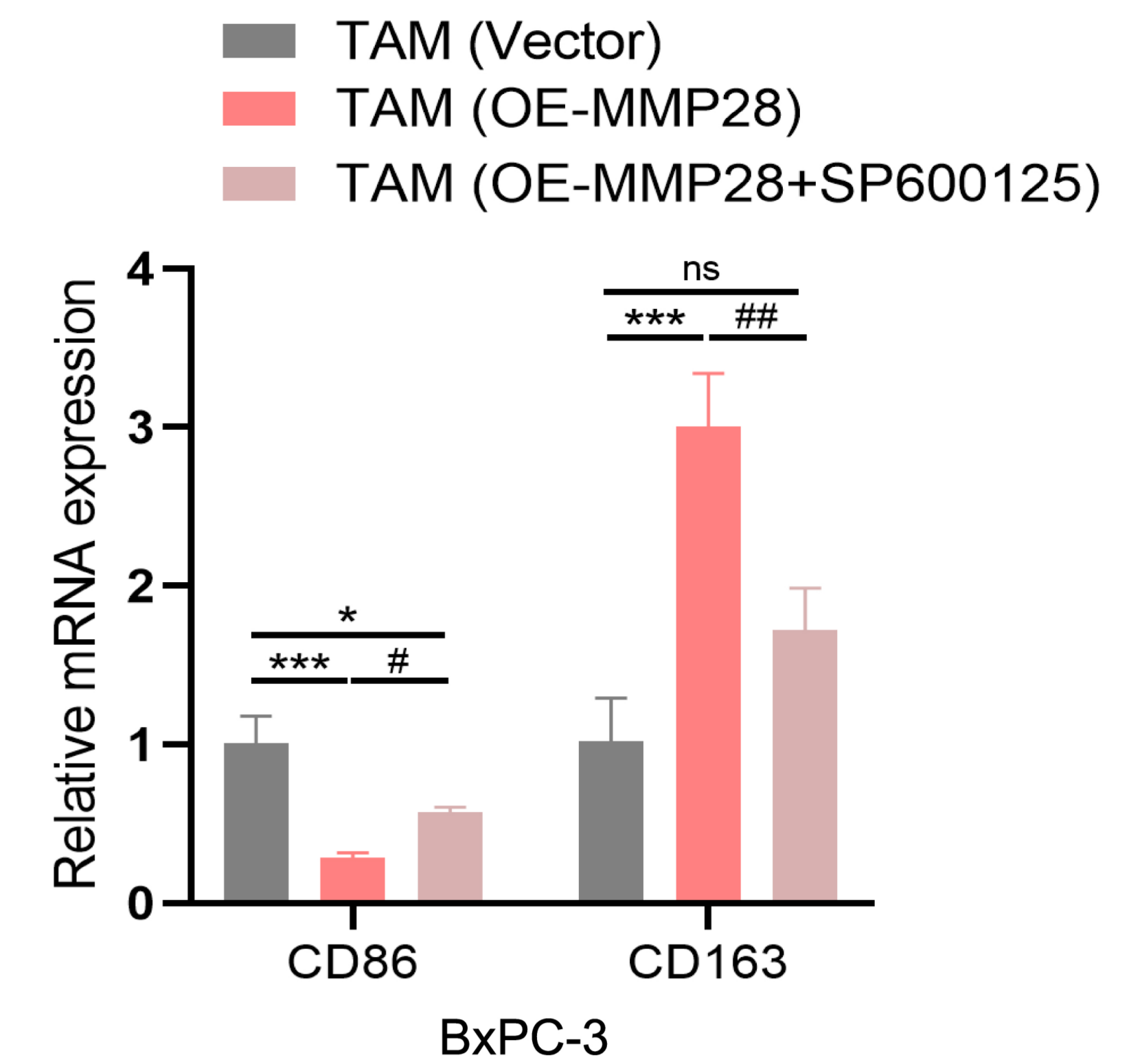

C

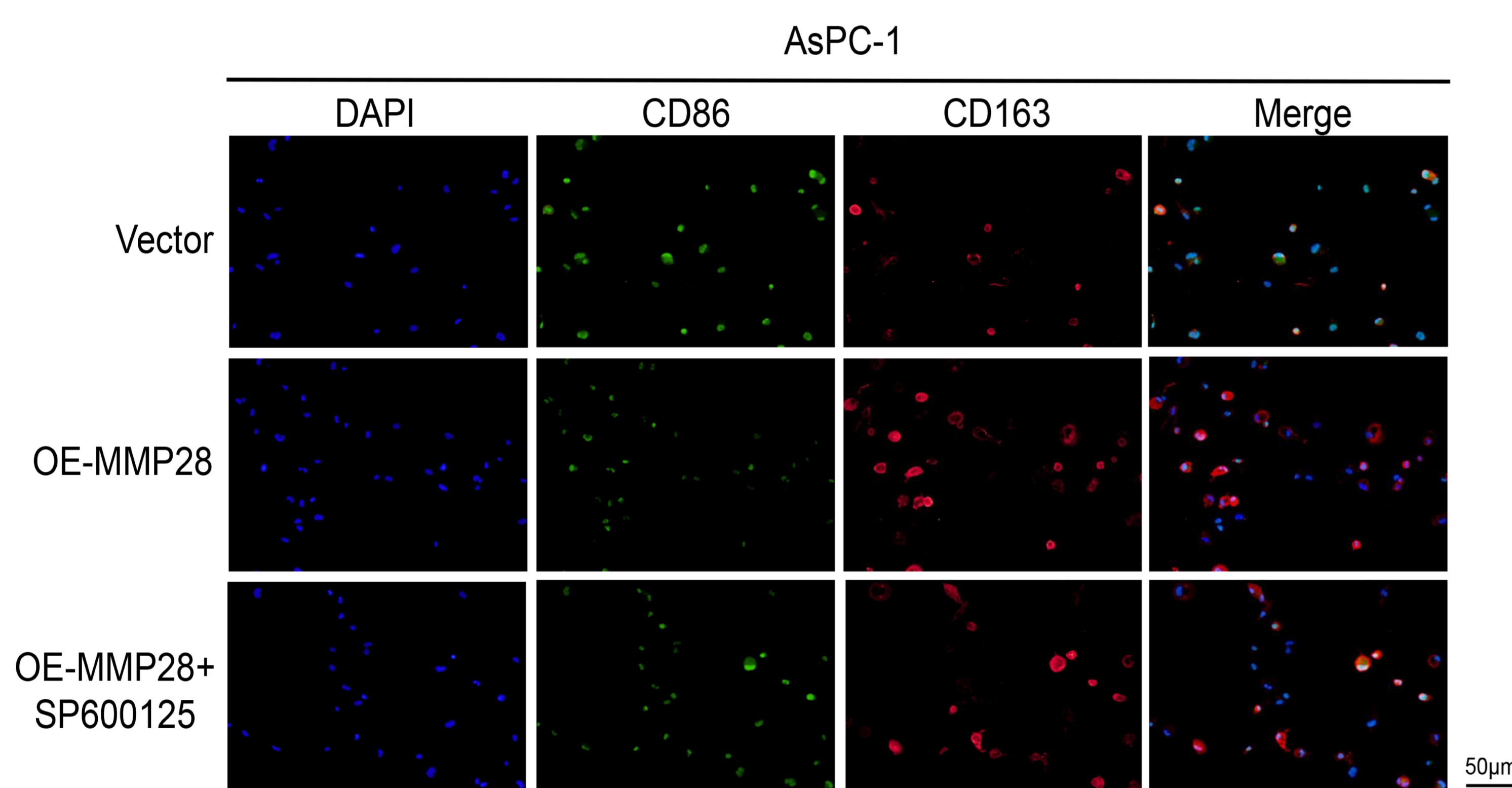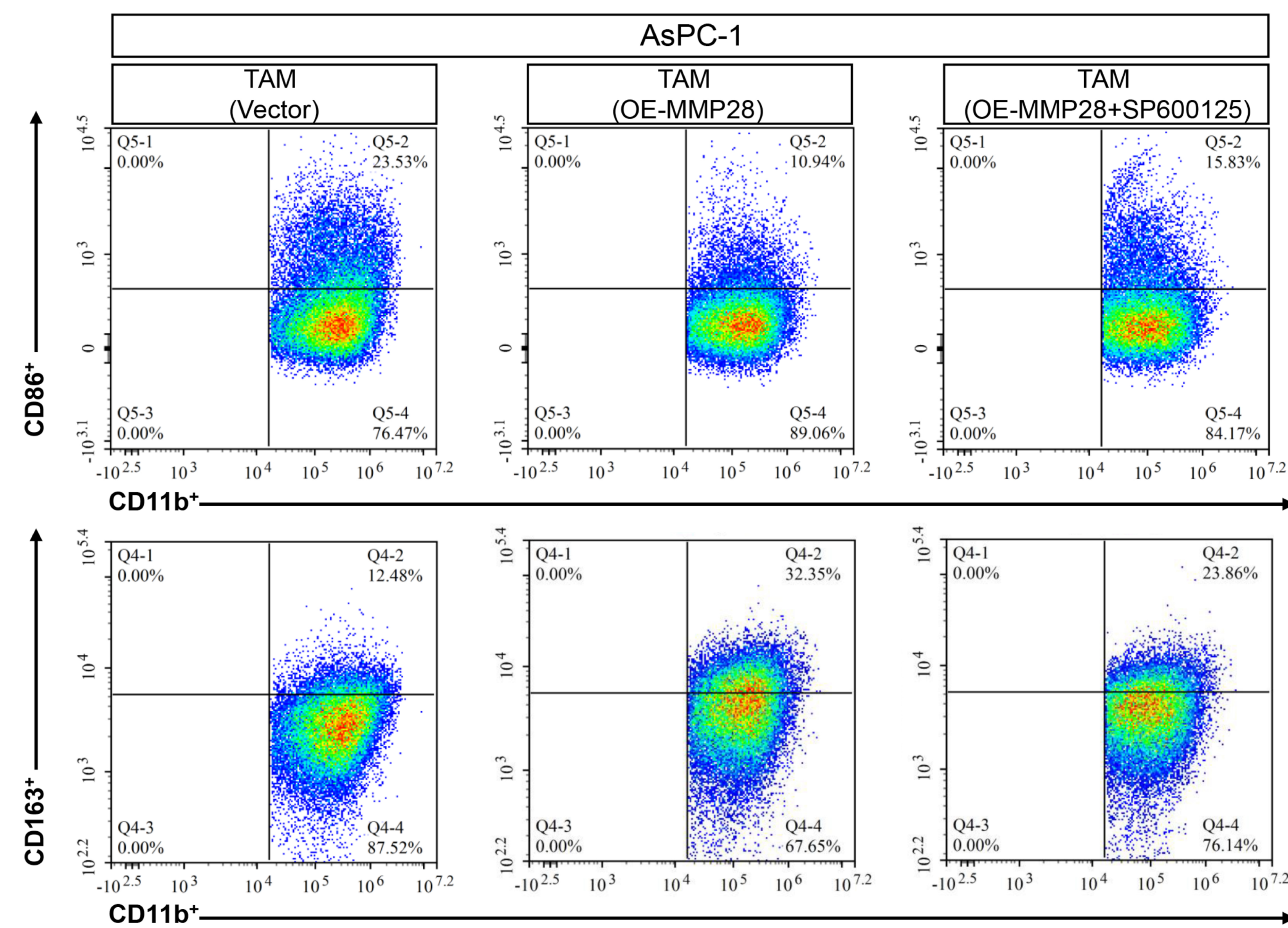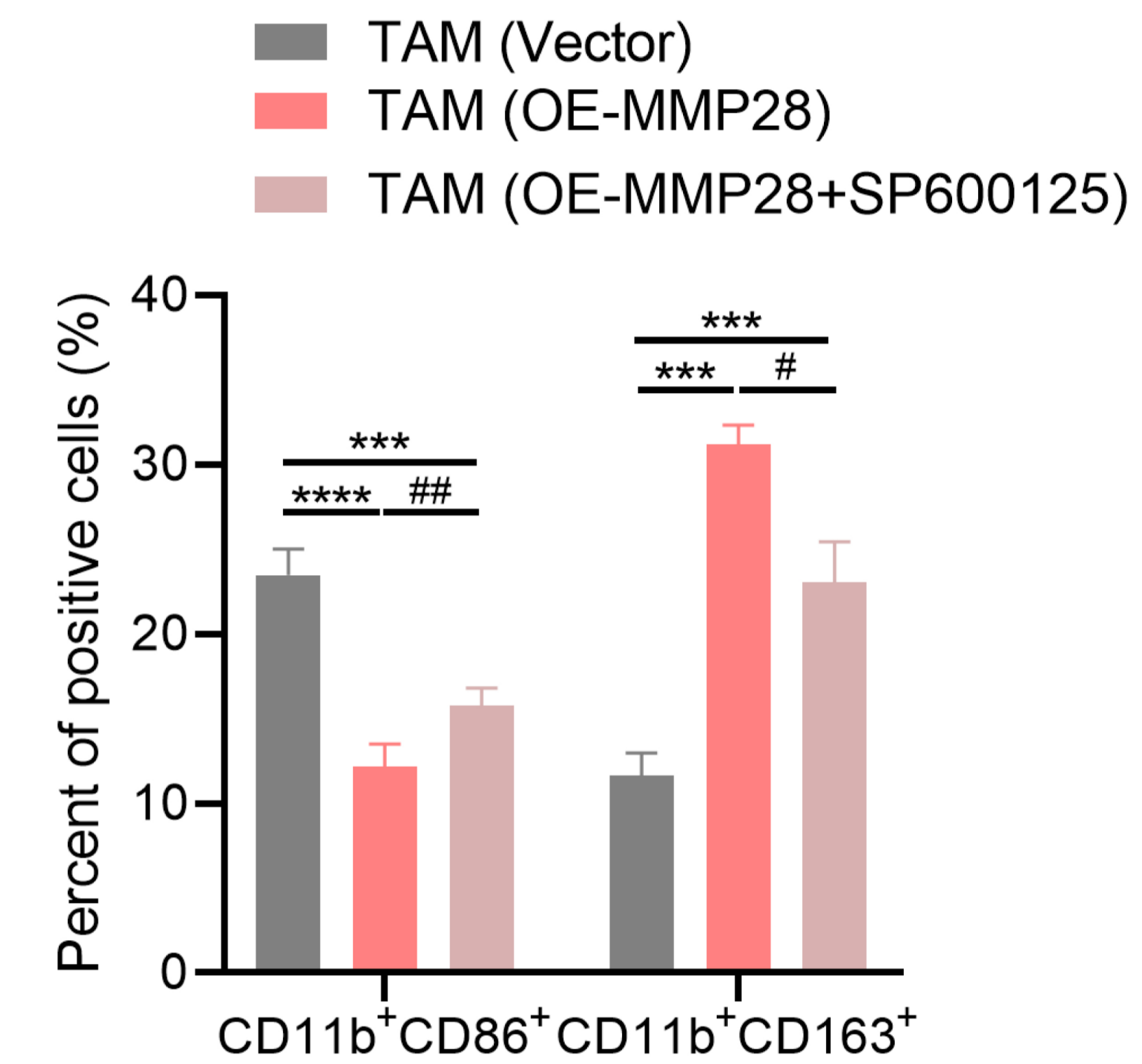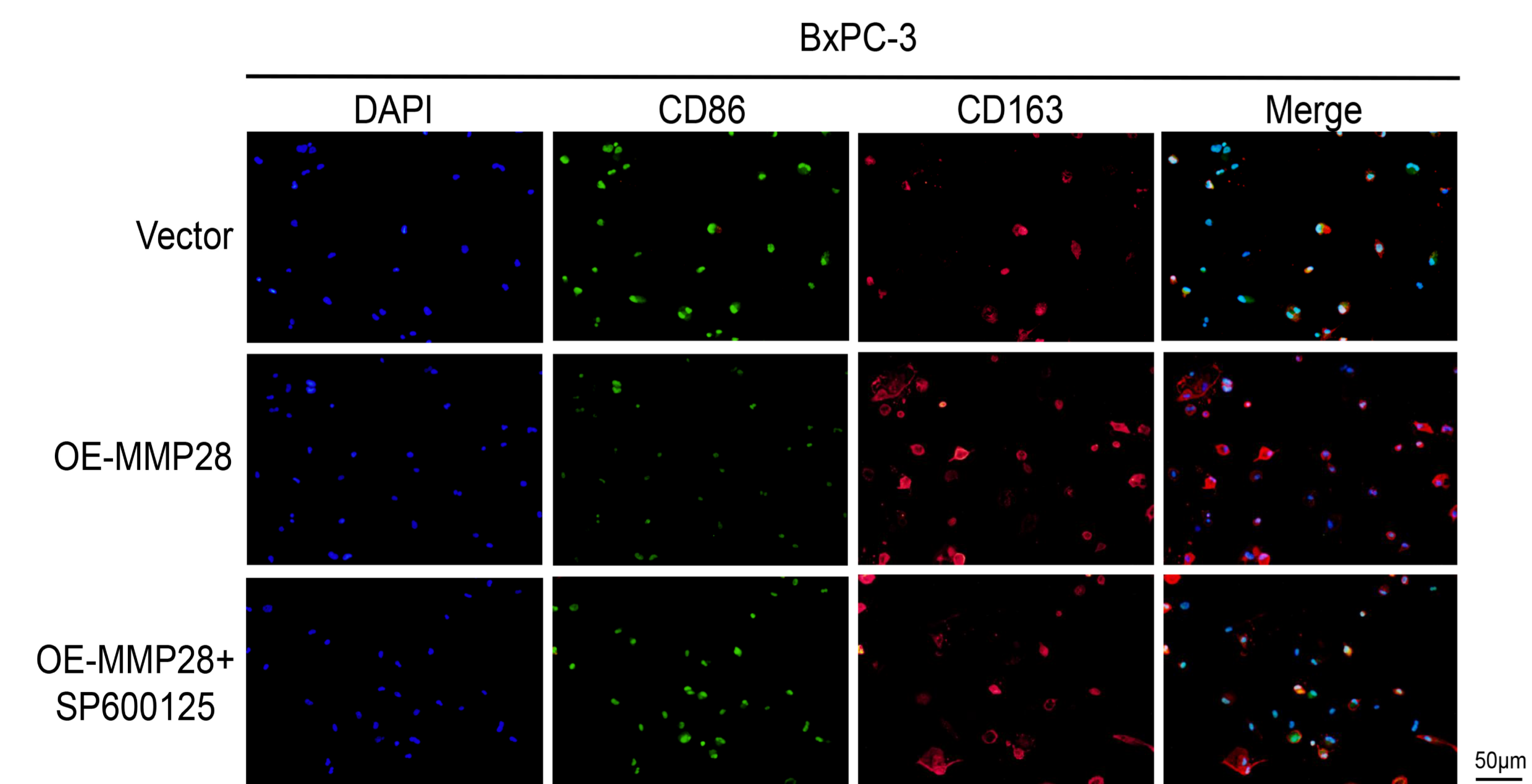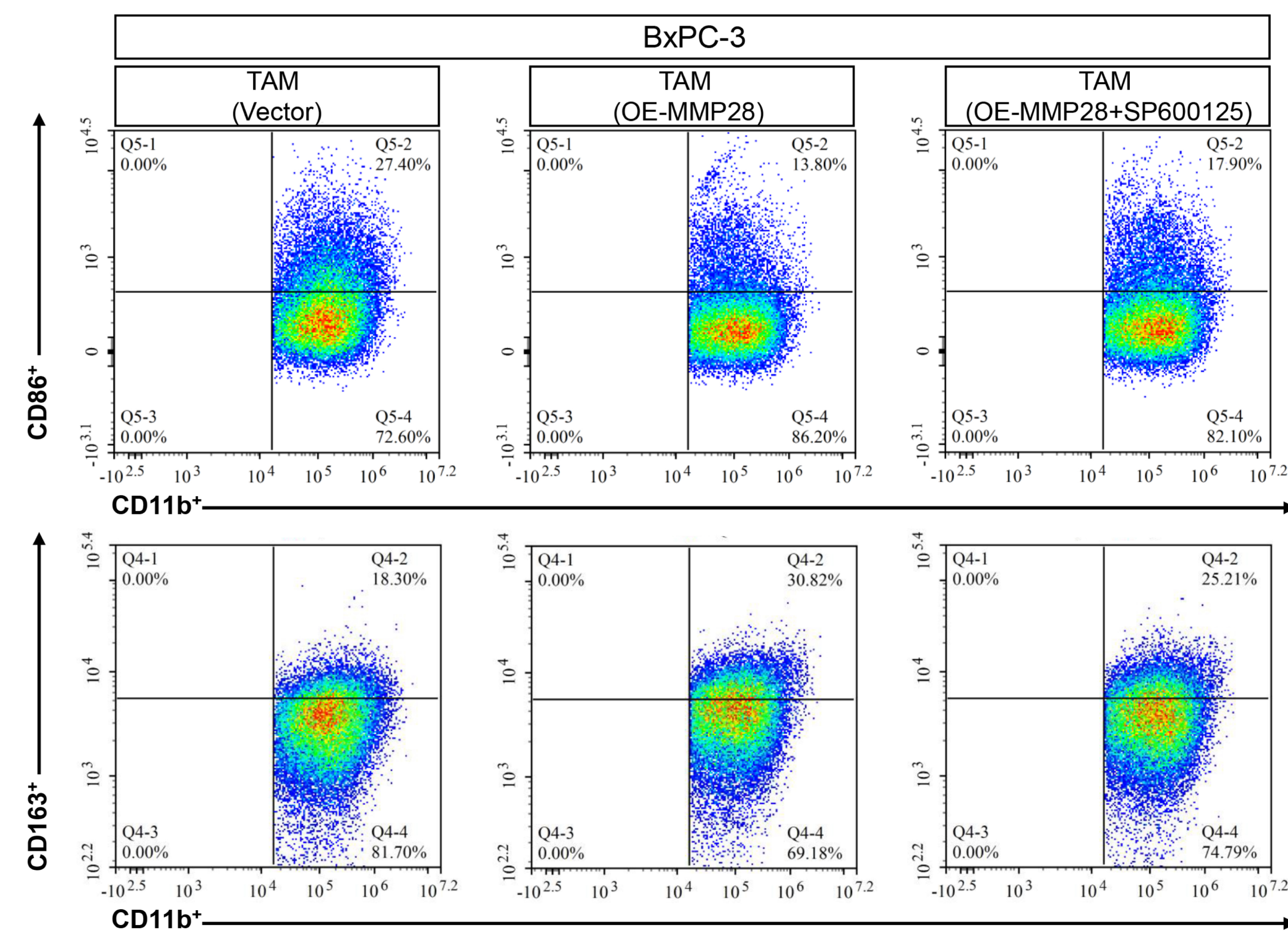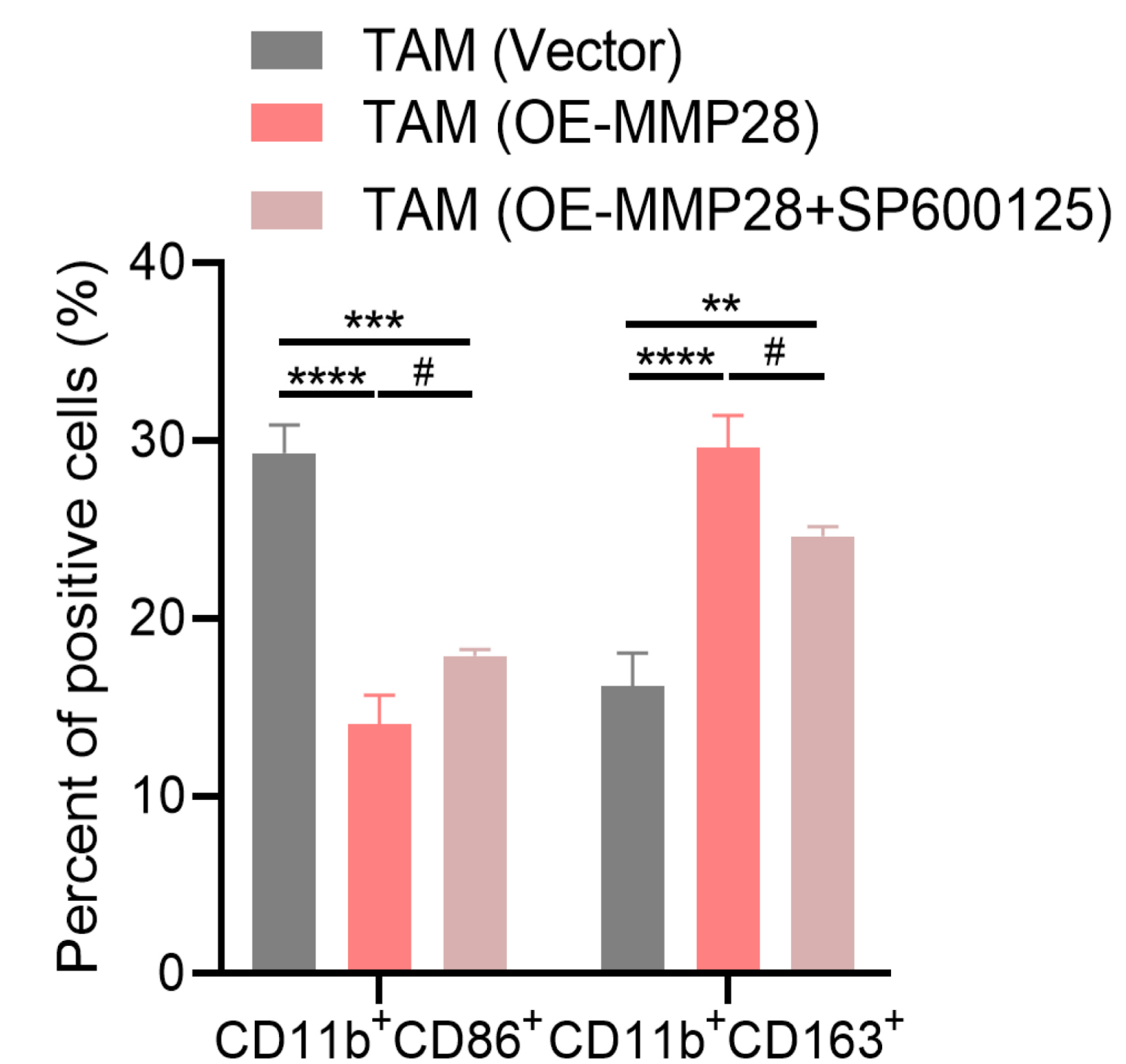

Supplement: Supplementary file 3 — Supplementary Material 3. Figure S3. MMP28 overexpression promotes the migration of TAMs and polarization of the M2 TAMs. (A) The ability of CM from cancer cells in the Vector group and the OE-MMP28 group to attract TAMs was assessed in 8 μm Transwell chambers. Compared with CM from the OE-MMP28 group, the effects of the addition of the JNK inhibitor SP600125 on the ability of cancer cells to promote TAM migration were determined. Scale bar, 100 μm. (B) qRT-PCR was used to determine the expression levels of CD86 and CD163 TAM markers, in different coculture groups. (C) The expression levels of the TAM markers CD86 and CD163 in different coculture groups were determined by immunofluorescence staining. Scale bar, 50 μm. (D) The proportions of CD11b+CD86+ TAMs and CD11b+CD163+ TAMs in different coculture groups were analysed by flow cytometry. *P<0.05, **P<0.01,***P<0.001, and ****P<0.0001. #P<0.05, ##P<0.01, and ####P<0.0001. [file 13046_2025_3321_MOESM3_ESM.pdf]

A

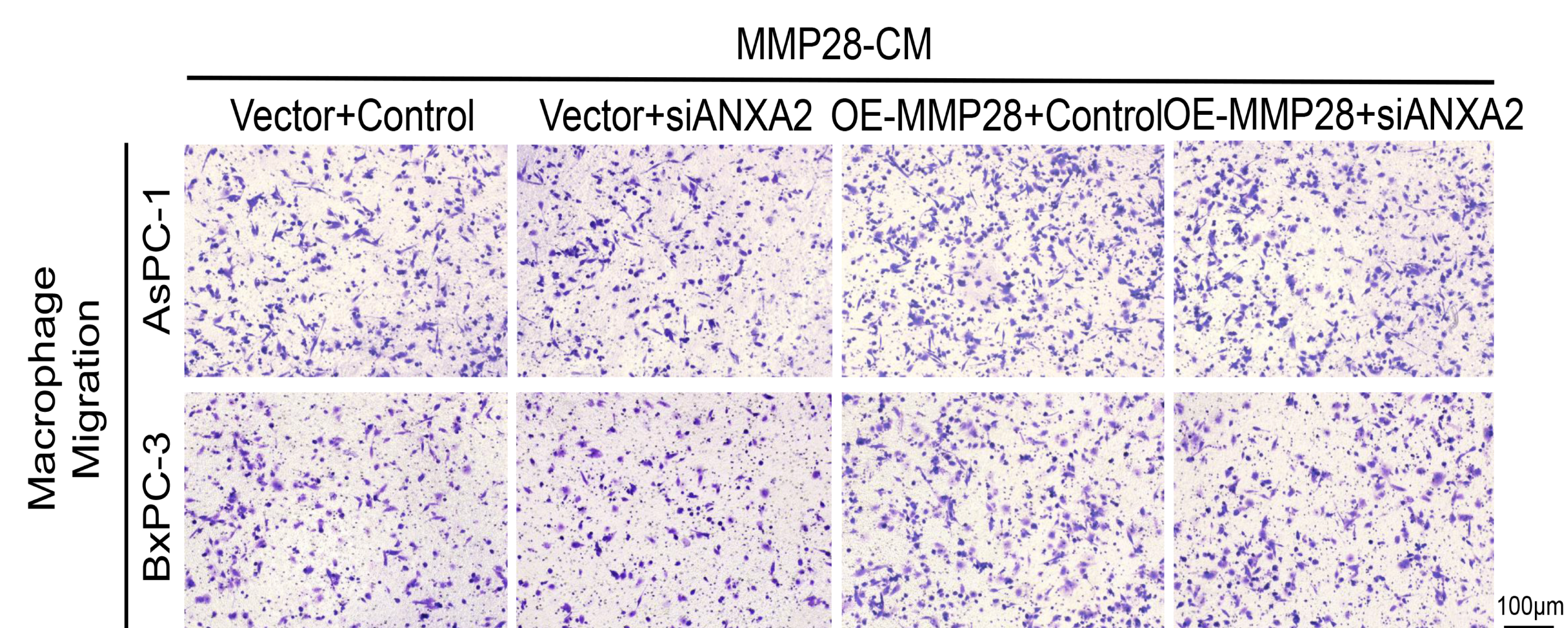

B

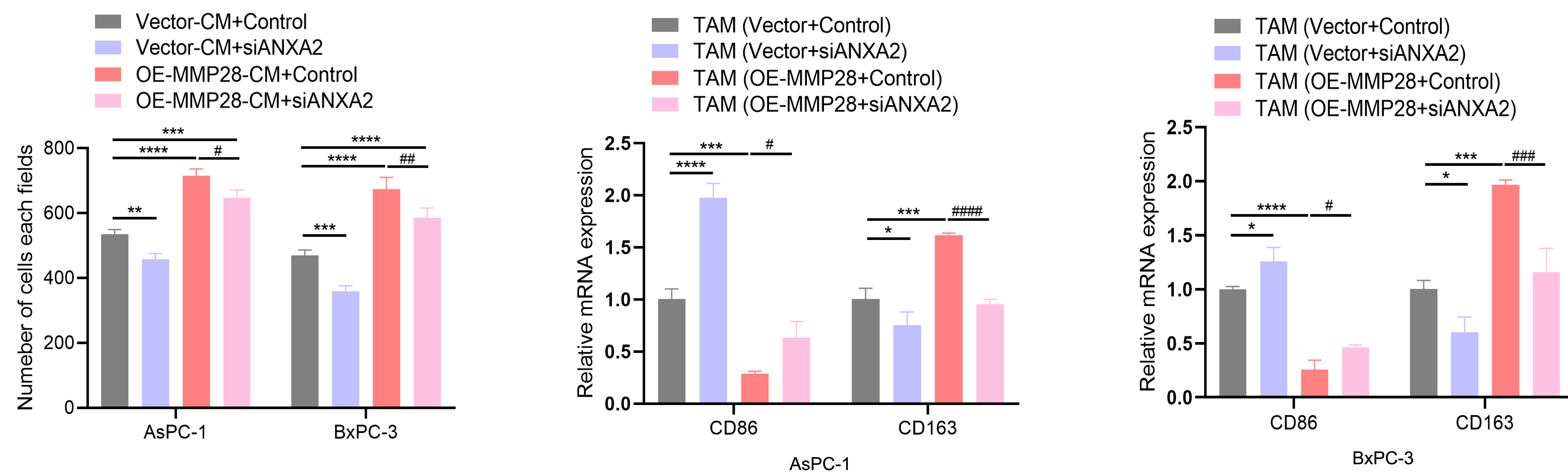

C

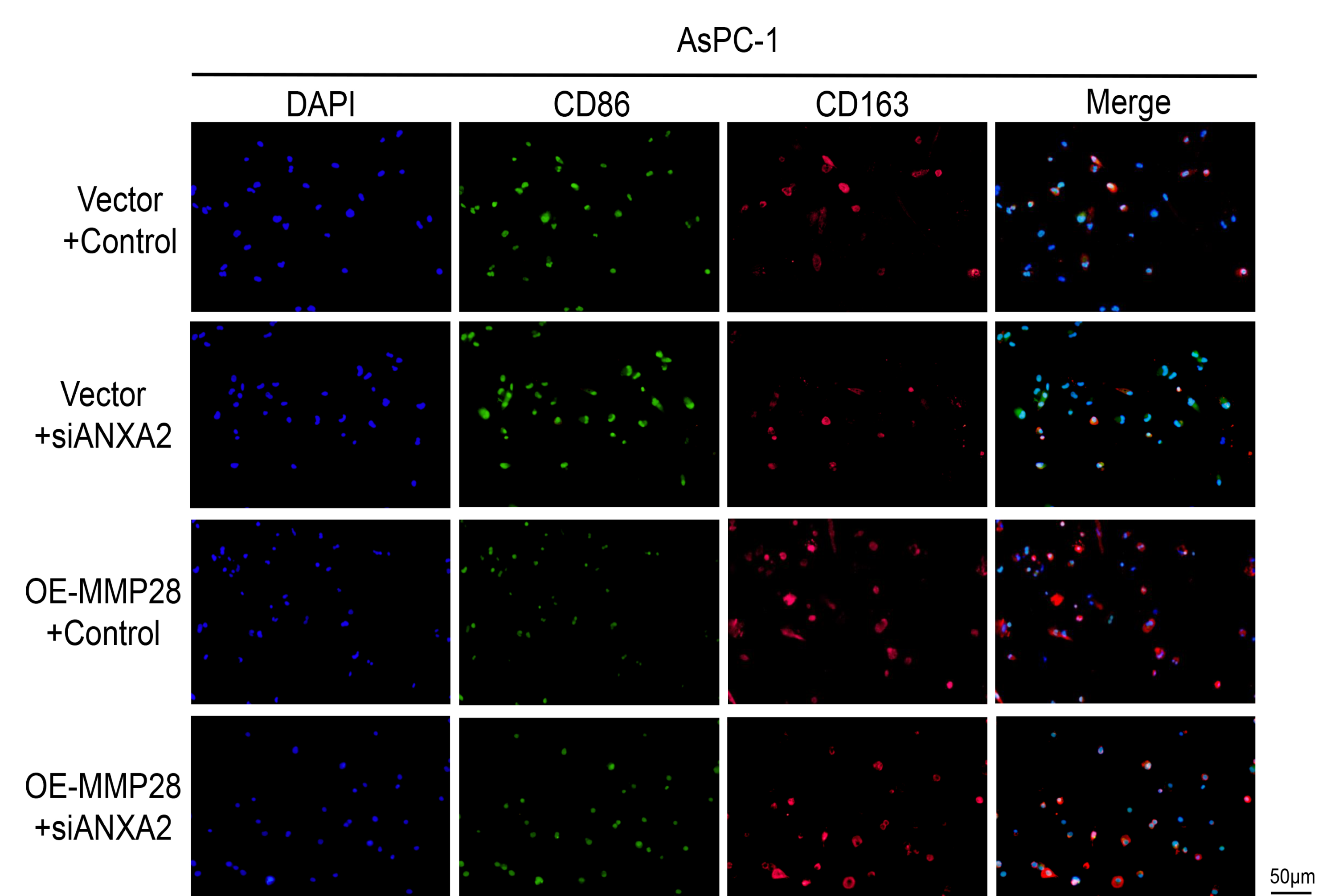

D

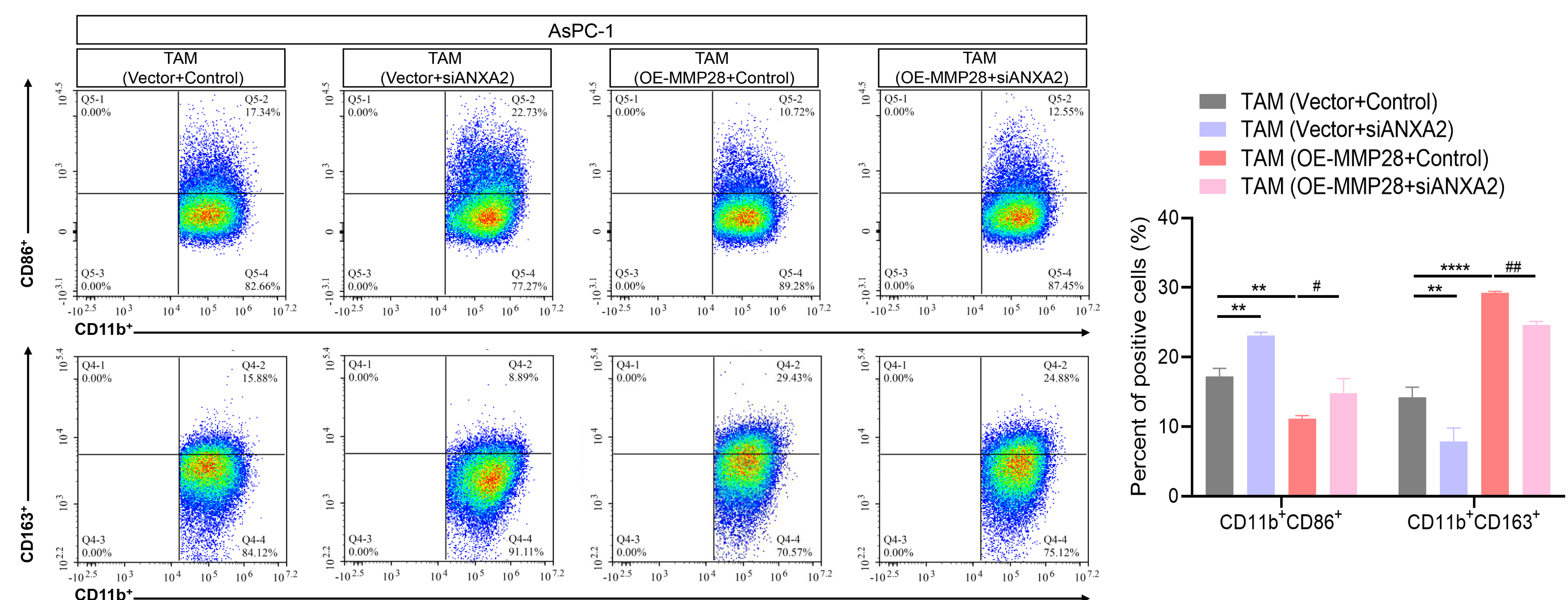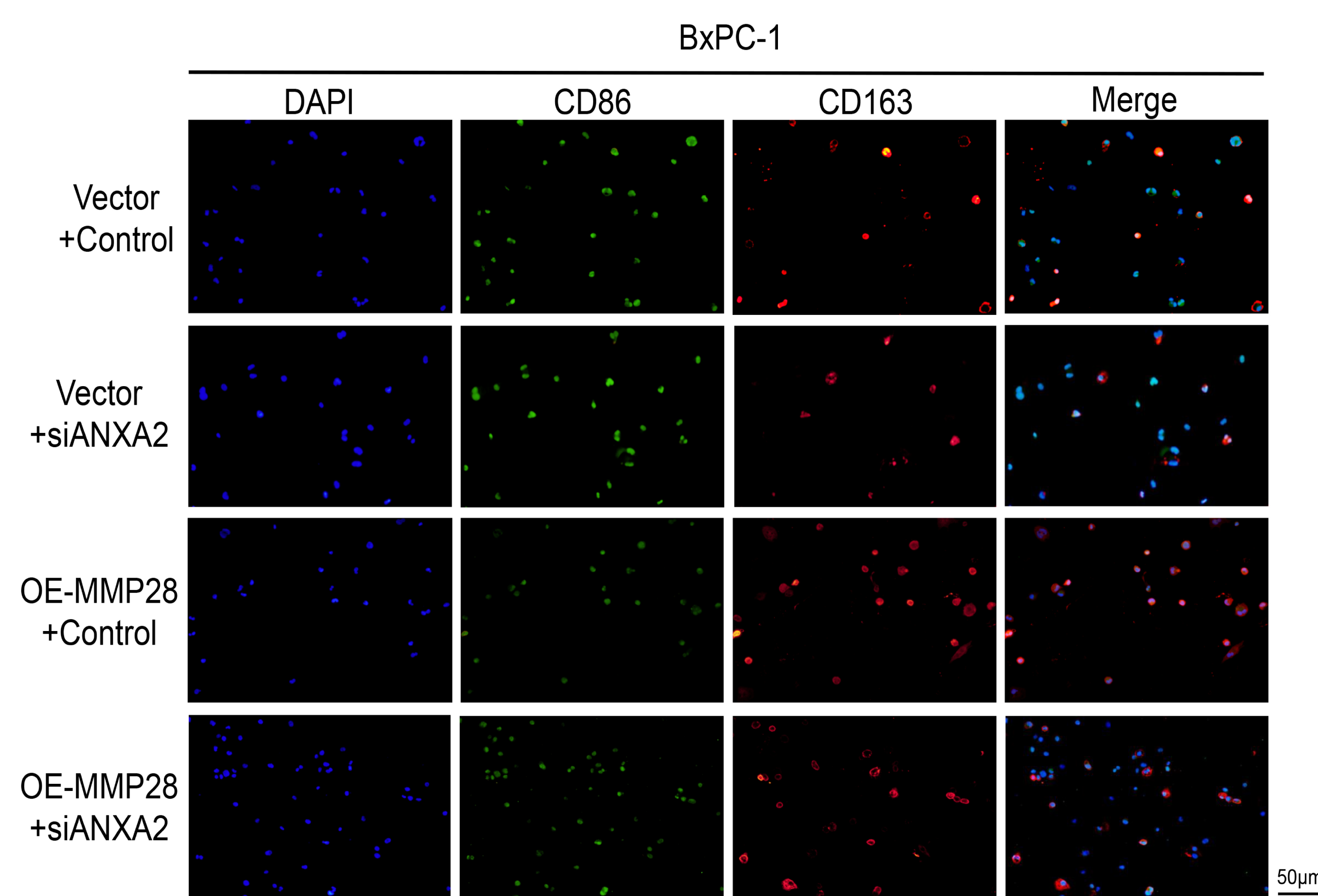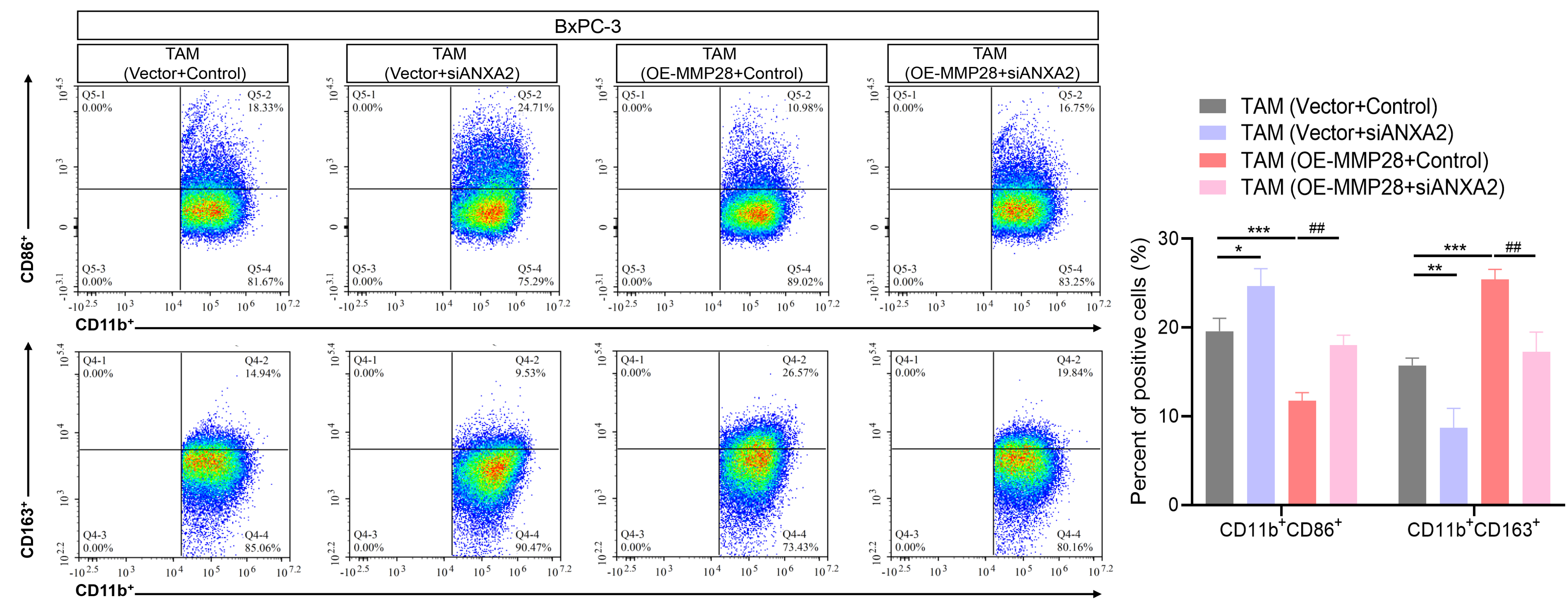

Supplement: Supplementary file 4 — Supplementary Material 4. Figure S4. ANXA2 enhances the ability of MMP28 to promote the migration of TAMs and the polarization of M2 TAMs. (A) The ability of CM from cancer cells to attract TAMs in the Vector+Control group, siANXA2 group, OE-MMP28+Control group and OE-MMP28+siANXA2 group was determined with 8 μm Transwell chambers. Plasmids containing small interfering RNAs specific for ANXA2 and empty plasmids were added to Vector group and OE-MMP28 group, respectively. Scale bar, 100 μm. (B) The expression levels of the TAM markers CD86 and CD163 in cancer cells cocultured with the Vector+Control group, siANXA2 group, OE-MMP28+Control group, and OE-MMP28+siANXA2 group were determined by qRT-PCR. (C) The expression levels of TAMs markers CD86 and CD163 in different coculture groups were determined by immunofluorescence staining. Scale bar, 50 μm. (D) The proportions of CD11b+CD86+ TAMs and CD11b+CD163+ TAM in the above co-culture group were analyzed by flow cytometry. *P<0.05, **P<0.01, ***P<0.001, and ****P<0.0001. and #P< 0.05, ##P<0.01, ###P<0.001. [file 13046_2025_3321_MOESM4_ESM.pdf]

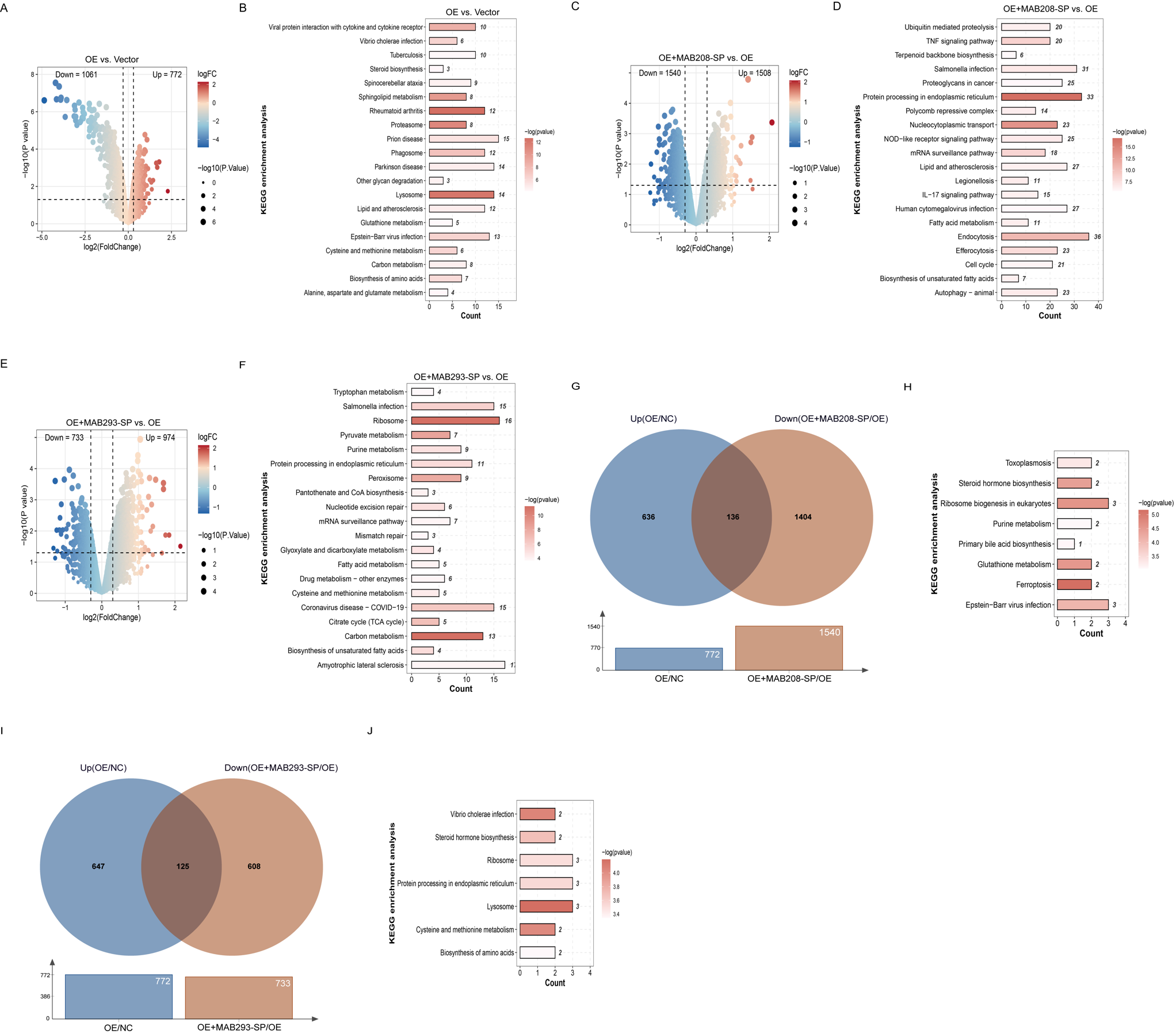

Supplement: Supplementary file 5 — Supplementary Material 5. Figure S5. MMP28 induces M2 TAM polarization by promoting amino acid metabolism in TAMs. (A-B) RNA sequencing was performed on TAMs cocultured with control (Vector) and MMP28-overexpressing (OE-MMP28) AsPC-1 cells. Volcano mapping and KEGG pathway enrichment analysis were subsequently performed for differentially expressed genes in TAMs. (C-D) RNA sequencing was performed on TAMs cocultured with AsPC-1 cells of MMP28-overexpressing group (OE-MMP28) and MMP28-overexpressing+MAB208-SP group (OE-MMP28+MAB208-SP), and the differentially expressed genes in TAMs were mapped by volcano plot and analysed by KEGG pathway enrichment. (E-F) RNA sequencing was performed on TAMs cocultured with AsPC-1 cells of the MMP28-overexpressing group (OE-MMP28) and the MMP28-overexpressing+MAB293-SP group (OE-MMP28+MAB293-SP), and the differentially expressed genes were mapped by a volcano plot and analysed by KEGG pathway enrichment. (G-H) The overlap of upregulated differentially expressed in TAMs cocultured with OE-MMP28 cells with downregulated differentially expressed genes in TAMs cocultured with OE-MMP28+MAB208-SP cells identified by RNA sequencing was determined and analysed for KEGG pathway enrichment. (I-J) The overlap of upregulated differentially expressed genes in TAMs cocultured with OE-MMP28 cells with downregulated differentially expressed genes in TAMs cocultured with OE-MMP28+MAB293-SP cells identified by RNA sequencing were taken and analyzed for KEGG pathway enrichment. [file 13046_2025_3321_MOESM5_ESM.pdf]
