# Supplementary material for: MMP28 recruits M2-type tumor-associated macrophages through MAPK/JNK signaling pathway-dependent cytokine secretion to promote the malignant progression of pancreatic cancer
Source: J Exp Clin Cancer Res. 2025 Feb 19;44:60. doi: 10.1186/s13046-025-03321-x (PMC11837641; doi:10.1186/s13046-025-03321-x)
Supplement: Supplementary file 6 — Supplementary Material 6. [file 13046_2025_3321_MOESM6_ESM.docx]

Supplementary Table 1. List of shRNA and siRNA sequences

| **Targeted Genes** | **Sequence (5′-3′)** |
| --- | --- |
| sh-MMP28-NC | TTCTCCGAACGTGTCACGT |
| sh-MMP28-1 | GCATTCCTAGAGAAGTACGGA |
| sh-MMP28-2 | GCGCAAGGAGGCGGAGGCATT |
| si-ANXA2-Control (sence) | UUCUCCGAACGUGUCACGUTT |
| si-ANXA2-Control (anti-sence) | ACGUGACACGUUCGGAGAATT |
| si-ANXA2 (sence) | UUGCUGAUCGGCUGUAUGATT |
| si-ANXA2 (anti-sence) | UCAUACAGCCGAUCAGCAATT |
