# Supplementary material for: MMP28 recruits M2-type tumor-associated macrophages through MAPK/JNK signaling pathway-dependent cytokine secretion to promote the malignant progression of pancreatic cancer
Source: J Exp Clin Cancer Res. 2025 Feb 19;44:60. doi: 10.1186/s13046-025-03321-x (PMC11837641; doi:10.1186/s13046-025-03321-x)
Supplement: Supplementary file 7 — Supplementary Material 7. [file 13046_2025_3321_MOESM7_ESM.docx]

Supplementary Table 2. List of primer sequences used for the qRT-PCR analysis

| **Name** | **(5′-3′)** | **Primer sequence (5′-3′)** |
| --- | --- | --- |
| MMP28 | Forword | CAATGAACAGGTCCCCAAAG |
|  | Reverse | GGCCGCATAACTGTTGGTAT |
| GAPDH | Forword | AAGGTGAAGGTCGGAGTCAAC |
|  | Reverse | GGGGTCATTGATGGCAACAATA |
| CD86 | Forword | TGCTCATCTATACACGGTTACC |
|  | Reverse | TGCATAACACCATCATACTCGA |
| CD163 | Forword | ATCAACCCTGCATCTTTAGACA |
|  | Reverse | CTTGTTGTCACATGTGATCCAG |
| IL-2 | Forword | ACCTCAACTCCTGCCACAATGTAC |
|  | Reverse | ATGCTCCAGTTGTAGCTGTGTTTTC |
| IL-5 | Forword | AGCCAATGAGACTCTGAGGATTCC |
|  | Reverse | TTGACTCTCCAGTGTGCCTATTCC |
| IL-8 | Forword | CCAAGAATCAGTGAAGATGCCAGTG |
|  | Reverse | ACCCTACAACAGACCCACACAATAC |
| MCP-1 | Forword | CTTCTGTGCCTGCTGCTCAT |
|  | Reverse | CGGAGTTTGGGTTTGCTTGTC |
| VEGFA | Forword | TTCGCTTACTCTCACCTGCTTCTG |
|  | Reverse | GGGCTGCTTCTTCCAACAATGTG |
| ANXA2 | Forword | GAGCGGGATGCTTTGAACATT |
|  | Reverse | TAGGCGAAGGCAATATCCTGT |
